# Chemoenzymatic Asymmetric Synthesis of Pyridine‐Based α‐Fluorinated Secondary Alcohols

**DOI:** 10.1002/cbic.202100392

**Published:** 2021-10-07

**Authors:** Timo Broese, Peter Ehlers, Peter Langer, Jan von Langermann

**Affiliations:** ^1^ Institute of Chemistry Biocatalytic Synthesis Group University of Rostock Albert-Einstein-Str. 3 A 18059 Rostock Germany; ^2^ Graforce GmbH Johann-Hittorf-Str. 8 12489 Berlin Germany; ^3^ Institute of Chemistry Organic Chemistry University of Rostock Albert-Einstein-Str. 3 A 18059 Rostock Germany

**Keywords:** alcohol, enzymes, enantioselectivity, fluorine, heteroaromatic

## Abstract

Fluoro‐substituted and heteroaromatic compounds are valuable intermediates for a variety of applications in pharma‐ and agrochemistry and synthetic chemistry. This study investigates the chemoenzymatic preparation of chiral alcohols bearing a heteroaromatic ring with an increasing degree of fluorination in α‐position. Starting from readily available picoline derivatives prochiral α‐halogenated acyl moieties were introduced with excellent selectivity and 64–95 % yield. The formed carbonyl group was subsequently reduced to the corresponding alcohols using the alcohol dehydrogenase from *Lactobacillus kefir*, yielding an enantiomeric excess of 95–>99 % and up to 98 % yield.

## Graphical abstract

**Two for one**: A straightforward chemoenzymatic synthesis pathway towards valuable chiral alcohols with α‐fluorination in close proximity to the chiral center and a pyridine ring is presented. High conversions with excellent enantioselectivities were found, showing its high synthetic potential.

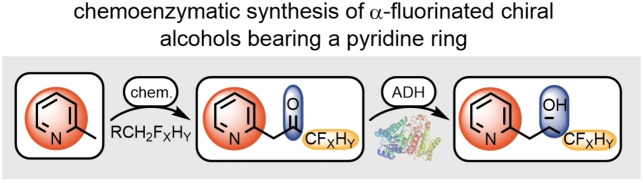

## Introduction

Enantiopure heteroaromatic alcohols are valuable compounds for a series of applications in pharma‐ and agrochemistry. Especially compounds with interesting structural features, e. g. a chiral secondary alcohol function in combination with a pyridine side chain, are important for many pharmacological relevant compounds^[1]^ and ligands for catalysts of asymmetric synthesis.^[2]^ Major examples include the use as glucocorticoid mimetics for the treatment of allergic, immune or inflammatory disorders, rheumatic diseases or help to overcome organ transplant rejection.^[3]^ Nevertheless, besides their desired anti‐inflammatory effects glucocorticoids suffer from harmful side effects like alterations in electrolyte or fluid balance, edema till development of diabetes mellitus or osteoporosis.^[4]^ The research aims to reduce these adverse effects by introducing new mimetics with equal potential concerning the anti‐inflammatory effects combined with a reduced rate of adverse effects. A special option is the use of fluorinated structural motifs, as these enable beneficial biological properties with a growing interest for pharmaceutically‐ and agrochemical relevant compounds.^[5]^ For example, the CHF_2_ moiety is a valuable target as it is a bioisostere of hydroxy, thiol and amide groups.^[6]^

To synthesize such chiral alcohols typically prochiral ketones are converted via an asymmetric reduction reaction to the desired chiral alcohol. Frequently used catalysts for this kind of transformation are homogeneous catalysts such as transition metal complexes.^[7]^ In contrast, biotransformations became a powerful alternative and such versatile biological catalysts frequently outperform classical chemical ketone reduction reactions under specifically mild reaction conditions and avoid the involvement of potentially toxic metals in the synthesis of the desired drug.^[8]^ Aside kinetic resolutions with hydrolases, alcohol dehydrogenases (ADH) or keto reductases (KRED) have been reported, which similarly catalyze highly regioselective the enantioselective reduction reaction and are available with both (*R*)‐ and (*S*)‐enantioselectivity.^[9]^ However, within the focus on α‐halogenation typically only substrates with classical benzylic derivates were reported,[^10^, ^11^] whereas the multi‐step synthesis of molecules bearing heteroaromatic structural motives are very rarely investigated.

The aim of this study is to investigate a straight‐forward synthetic approach to close this gap towards chiral secondary alcohols bearing a pyridine ring and different grades of α‐halogenation in close proximity to the chiral center. This was achieved through a straight‐forward two‐step chemoenzymatic approach (Table 1). The proposed synthetic route starts from readily available picoline derivatives **1** forming the prochiral α‐halogenated ketones **2**. The subsequent enantioselective reduction of the formed carbonyl group to the chiral alcohol **3** was investigated exemplarily with the alcohol dehydrogenase from *Lactobacillus kefir*.

**Table 1:** Chemoenzymatic conversion of picolines and derivatives thereof **1** to α‐halogen substituted chiral alcohols bearing a pyridine group **3**.

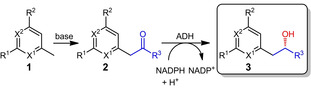											

**1**–**3**	R^1^	R^2^	R^3^	X^1^	X^2^	**1**–**3**	R^1^	R^2^	R^3^	X^1^	X^2^
**a**	H	H	CH_3_	CH	N	**h**	H	H	CF_3_	N	CH
**b**	H	H	CH_2_F	CH	N	**i**	H	H	CClF_2_	N	CH
**c**	H	H	CHF_2_	CH	N	**j**	CH_3_	H	CH_3_	N	CH
**d**	H	H	CF_3_	CH	N	**k**	CH_3_	H	CF_3_	N	CH
**e**	H	H	CH_3_	N	CH	**l**	CH_3_	CH_3_	CH_3_	N	CH
**f**	H	H	CH_2_F	N	CH	**m**	CH_3_	CH_3_	CF_3_	N	CH
**g**	H	H	CHF_2_	N	CH

## Results

### Chemical synthesis of heteroaromatic prochiral ketones

In the first step the *ortho*‐ or *para*‐positioned methyl group of picoline derivatives were converted to the corresponding ketones by the use of the bases *n*‐butyllithium (*n*BuLi), lithium diisopropyl amide (LDA) or pyridine to form the exocyclic deprotonated intermediate. The different α‐halogenated acyl moieties were introduced through the use of different esters or dimethylacetamide for simple methyl ketones (DMA) (Table 2). The straight‐forward one pot synthesis was followed by a workup consisting of an aqueous extraction, while in some examples an additional column chromatography was required.

**Table 2:** Base‐induced conversion of picolines **1** **a**–**m** to the corresponding prochiral ketones.

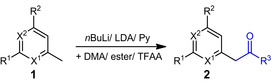							

**2**	Base	Ester/DMA	Yield [%]	**2**	Base	Ester/DMA	Yield [%]
**a**	LDA	DMA	**65**	**h**	Pyridine	TFAA	**64** ^[a]^
							
**b**	LDA	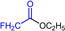	**95**	**i**	*n*BuLi	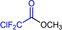	**84**
							
**c**	LDA	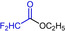	**84**	**j**	*n*BuLi	DMA	**90**
							
**d**	LDA	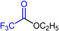	**81**	**k**	*n*BuLi	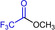	**90**
							
**e**	*n*BuLi	DMA	**84**	**l**	*n*BuLi	DMA	**82**
							
**f**	*n*BuLi	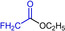	**95**	**m**	*n*BuLi	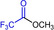	**91**
							
**g**	*n*BuLi	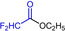	**72**

LDA=lithium diisopropyl amide; *n*BuLi=*n*‐butyllithium; DMA=dimethylacetamide; TFAA=trifluoroacetic anhydride. [a] Synthesized according to Kawase et al.^[12]^

All ketones could be synthesized in satisfying isolated yields from 64 % up to 95 %. This shows that the applied procedure can in generally be used to form any grade of α‐halogenation in methyl ketones with such a heteroaromatic side chain. During all conversions no issues in terms of regioselectivity or nucleophilic attack of the butylanion or a second lithiated picoline moiety were noticed. In addition, the obta–ined prochiral ketones can easily be converted to the racemic secondary alcohols *rac* **3** **a**–**3** **m** by an excess of sodium borohydride in methanol. The respective yields are between 65 % and 99 % after aqueous workup with an optional subsequent column chromatography (see supporting document).

### Enzymatic conversion to enantiopure secondary alcohols

The subsequent enzymatic conversion was performed in a monophasic reaction system consisting of phosphate buffer pH 7.0 containing 0.1 mM MgCl_2_ and the dissolved substrate. In case the substrate was not soluble in the buffer system a two‐phase system of additional 50 % (v/v) MTBE was used as a two‐phase system.^[13]^ As a biocatalyst, the ADH from *Lactobacillus kefir* was chosen, as it exhibits exceptionally high enantioselectivity for classical methyl ketones^[14]^ aside the well‐studied ADHs from *Lactobacillus brevis* and *Rhodococcus ruber*.^[15]^ The final reaction system, including the substrate coupled cofactor regeneration of NADP^+^ via isopropanol, is shown in Table 3. The reaction system was heated to 30 °C within 15 min and then the reaction time of 48 h was started by an addition of the enzyme. The crude product mixture was purified by aqueous workup followed by column chromatography. The obtained results show that almost half of the investigated substrates were successfully transformed into the respective chiral alcohols.

**Table 3:** Enzymatic reduction of the picoline‐based ketones to the corresponding chiral alcohols.

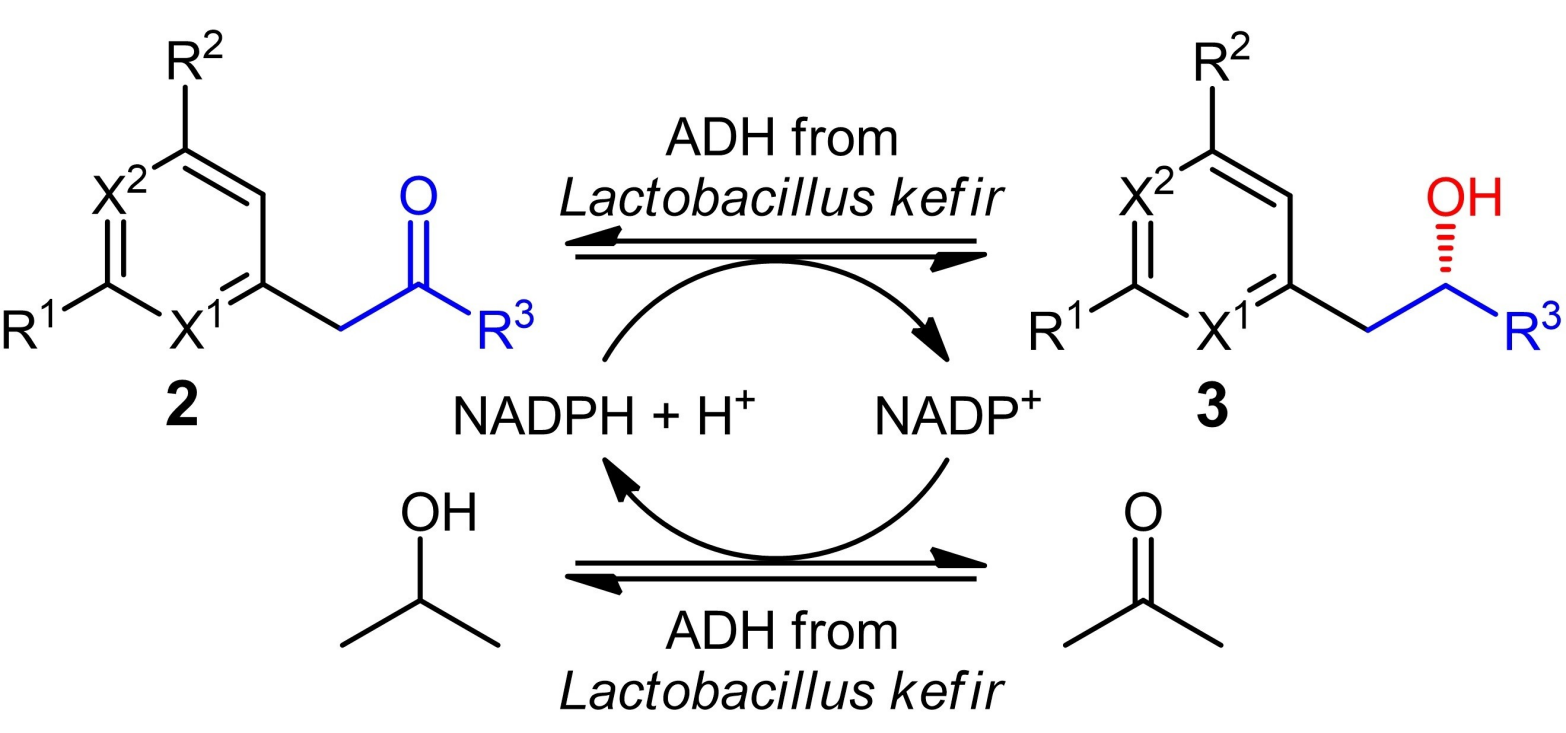							

**3**	Yield [%]	e.e.^[a]^ [%]	[α]_598_^[b]^	**3**	Yield [%]	e.e.^[a]^ [%]	[α]_598_^[b]^
**a**	93	>99(*R*)	−15.84(28)	**h**	0	–	–
**b**	98	n.d.	+23.69(26)	**i**	0	–	–
**c**	95	>99(*S*)	+1.04(27)	**j**	0	–	–
**d**	0	–	–	**k**	0	–	–
**e**	36	>99(*R*)	−37.73(27)	**l**	0	–	–
**f**	70	95(*S*)	−17.17(28)	**m**	0	–	–
**g**	60	n.d.	+13.21(22)^[c]^

[a] Absolute configuration in parenthesis based on comparison of GC spectra with the corresponding racemic alcohol. [b] 1.0 M in chloroform, temperature (°C) in parenthesis. [c] 1.0 M in dichloromethane.

The simple methyl ketones **3** **a** and **3** **e** were converted to the enantiopure (*R*)‐alcohol with 93 and 36 % yield, respectively. With an increasing degree of fluorination in α‐position, from CH_2_F to CHF_2_, successful conversions were obtained with 36‐98 % yield and high enantiomeric excess. It is also visible that 2‐picoline derivatives **3** **a**–**c** allowed higher conversions compared to their 4‐substituted analogues **3** **e**–**g**, which highlights the preference of the applied ADH. The reason for this behavior seems to be based on the chelating effect of the β‐enaminketones due to their one electron donor ability. This might also deactivate the enzyme by chelating the central magnesium ion. In addition, a trifluoromethyl group in α‐position to the carbonyl group was not accepted for both isomers **3** **d** and **3** **h**. Such a behavior was expected as lower activities were reported for trifluoromethyl acetophenone with the closely related ADH from *Lactobacillus brevis*.^[10]^ An identical result was found for the even larger difluoromonochloro group (**3** **i**), which was not converted by the applied ADH. In contrast, unexpectedly the introduction of further methyl substituents at the heterocycle (**3** **j**–**m**) caused a full loss of enzymatic activity, which was not found in general for substituted acetophenone derivatives with such ADHs. This is probably based on size restrictions within the active site due to the additionally methyl group and the corresponding size increase of the heterocyclic domain. Finally, all obtained chiral alcohols show no decomposition or racemization over time when stored below 0 °C.

## Conclusion

The aim of this study was to examine the design of a chemoenzymatic synthesis route towards α‐halogenated ketones bearing a pyridine‐based heteroaromatic structural motive. The initial introduction of α‐halogenated acyl moieties onto 2‐ and 4‐picolines is based on a straight‐forward protocol yielding very good yields up to 95 %. The subsequent enzymatic reduction by the highly (*R*)‐selective alcohol dehydrogenase from *Lactobacillus kefir* facilitated several enantiopure alcohols with different levels of halogenation. This study highlights the synthetic potential and limits of this chemoenzymatic pathway, while substitutions at the heterocycle were not accepted as a substrate by the applied alcohol dehydrogenase. The results expand the product library with valuable heterocyclic alcohols as well as investigating the limits of the α‐fluorination in this conversion.

In summary, it was successfully shown that α‐halogenation can easily be used to convert even complex heterocyclic substrates with high yield to the corresponding enantiopure compounds.

## Experimental Section

### Chemicals

All required chemicals were obtained from commercial sources and used as received. Dry solvents were bought from Acros and used as received. The alcohol dehydrogenase from *Lactobacillus kefir* (lyophilized lysate) was obtained from Evocatal, Düsseldorf, Germany (now evoxx technologies GmbH).

### GC‐analysis

The enantiomeric excess of corresponding alcohols was analyzed by comparison of the racemic mixture with the enantioenriched alcohol. For analysis a HP 1100 with Chiralyser, DAD and RI Detector were used. As columns a Lipodex E, G or Chialdex β‐Ph within a temperature range between 120 and 180 °C were used.

### Enzyme assay

Enzymatic activity was determined photometrically with a UV/Vis spectrometer SPECORD 50 (Analytik Jena, Jena, Germany) by monitoring the decrease in intensity at 340 nm from the consumption of NADPH. Therein, 970 μL acetophenone (10 mM) in 50 mM TEA‐buffer with 1 mM MgCl_2_ (pH 7.0) and 10 μL enzyme solution in Tris/HCl‐buffer (100 mM, pH 7.2) with 1 mM MgCl_2_ were combined in a quartz cuvette, which was tempered at 25 °C. The measurement started by the addition of 20 μL NADPH in Tris/HCl‐buffer (1 mM). One unit (1 U) of enzyme activity was defined as the reduction of 1 mmol acetophenone per minute under standard assay conditions.

### Representative synthesis of α‐halogenated methyl ketones (2)

In a dry Schlenk flask with septum the corresponding picoline 1 (1 eq.) was dissolved in dry THF (1.4 ml/mmol) under argon atmosphere. The solution was cooled to −78 °C and *n*‐butyllithium solution (2.5 M, 1.1 eq.) or lithium diisopropylamide (1.1 eq) was added into the mixture. The resulting yellow to deep red solution was stirred for 1 h at −78 °C. To the resulting suspension dry dimethylformamide (DMA, 1.5 eq.) or the desired ester (1.5 eq.) was added drop wise and the mixture stirred for 30 min. After the suspension cleared up it was allowed to warm to room temperature (ca. 2 h). The reaction was subsequently quenched by a carful addition of aqueous 5 % HCl solution. Afterwards the solution was neutralized with aqueous 10 % NaHCO_3_ and extracted three times with 15 ml ethyl acetate. The combined organic phases were dried with anhydrous Na_2_SO_4_ and evaporated in vacuum. The crude product was further purified by flash chromatography.

### Representative synthesis of α‐halogenated chiral alcohols (3)

The enzymatic synthesis of the α‐halogenated chiral alcohols was performed in 33 mM phosphate buffer or an aqueous two‐phase system (ATPS) consisting of methyl *tert*‐butyl ether (MTBE) and 33 mM phosphate buffer pH 7.0 with 1 mM MgCl_2_) at 30 °C in a ratio of 1 : 1. After the addition of the substrate (0.15 M) and the enzyme (78 U, 1.0 mgml^−1^), NADP^+^ (0.5 mM) as cofactor and isopropanol (2.25 M)) for cofactor regeneration were added. The reaction mixture was heavily stirred over 48 h and monitored by TLC. For the monophasic reaction the reaction products were extracted after 48 h into MTBE. The respective organic layer was washed two times with 10 ml of water, dried with Na_2_SO_4_ and eventually evaporated in vacuo. The crude product was further purified by flash chromatography.

## Conflict of interest

The authors declare no conflict of interest.

## Supporting information

As a service to our authors and readers, this journal provides supporting information supplied by the authors. Such materials are peer reviewed and may be re‐organized for online delivery, but are not copy‐edited or typeset. Technical support issues arising from supporting information (other than missing files) should be addressed to the authors.

Supporting Information
