# Supplementary material for: Chemoenzymatic Asymmetric Synthesis of Pyridine‐Based α‐Fluorinated Secondary Alcohols
Source: Chembiochem. 2021 Oct 7;22(23):3314–8. doi: 10.1002/cbic.202100392 (PMC9293303; doi:10.1002/cbic.202100392)
Supplement: Supplementary file 1 — Supporting Information [file CBIC-22-3314-s001.pdf]

ChemBioChem

Supporting Information

Chemoenzymatic Asymmetric Synthesis of Pyridine-Based α-Fluorinated Secondary Alcohols

Timo Broese, Peter Ehlers, Peter Langer,* and Jan von Langermann*

CONTENT

1. General Information 2
2. Prochiral ketones (2a-m)...2
3. Racemic alcohols (rac-2a-2c and rac-2e-2g)...8
4. Enantioenriched alcohols (3a-2c and 2e-2g) 11
6. NMR-spectra for racemic alcohols (rac-3) 27
7. NMR Spectra for enantioenriched alcohols (3) 33
8. Gas chromatograms of chiral alcohols (3) 39

1. General Information

^{1}H, ^{13}C and ^{19}F NMR-Spektra were recorded on a Bruker AVANCE 250, 300 or 500 spectrometer. IR spectra were recorded on a Nicolet 6700 FT-IR-Spectrometer with Smart Orbit ATR-unit. Wave numbers are given in cm^{-1}. Melting points were recorded on an EZ-Melt, Stanford Research Systems or Micro-Hot-Stage GalenTM III (Cambridge Instruments). The heating rate was 20 °C/min (clear points are given) and all melting points are uncorrected. Mass spectra (MS) were recorded on a Finnigan MAT 95 XP; high resolution spectras are given with an accuracy of the ion peak with ±2 mDa. The specific rotation was recorded on a Gyromat-HP at 598 nm with a concentration of 10 $mgml^{-1}$ in the indicated solvent at the temperature in parenthesis. The ee value is calculated by comparison of the GC spectra with one of an authentic sample. The spectras were recorded on a HP 1100 (Hewlett Packard) with DAD (diode array detector), Chiralyser and RI-detector (refractive index detector). All dry solvents were bought from Acros Organics and used as received.

2. Prochiral ketones (2a-m)

1-(Pyridin-4-yl)propan-2-one (2a)

4-Picoline (**1a**) (10.1 mmol, 1.00 ml), lithium diisopropylamide (11.1 mmol, 11.1 ml, 1.0 M in THF) and dimethylacetamide (9.5 mmol, 0.90 ml) resulted in 1-(pyridin-4-yl)propan-2-on (**2a**) and was purified via flash chromatography resulting in a red oil (1.462 g, 65%).

1H NMR (250 MHz, CDCl3) δ = 2.21 (s, 3H, CH_3), 3.73 (s, 2H, CH_2), 7.15 (d, ^{3}J(H,H) = 6.0 Hz, 2H, Hetar), 8.56 (d, ^{3}J(H,H) = 6.0 Hz, 2H, Hetar); 13C NMR (63 MHz, $CDCl_3$) δ = 29.7 (1C, CH_3), 49.6 (1C, CH_2), 124.6 (2C, CH_{Hetar}), 142.7 (1C, C_{Hetar}), 149.9 (2C, CH_{Hetar}), 203.9 (1C, C=O); IR (ATR, cm^{-1}): $\tilde{\nu}$ = 3407, 3083, 3041, 3004, 2962, 2937, 2852, 1945, 1707, 1592, 1412, 1349, 591, 565, 546; MS (GC-MS): m/z (%) = 135 ($[M]^+$, 5), 94 (11), 93 (100), 92 (20), 65 (13), 43 (50), 39 (14); HRMS (EI): calcd. for C_8H_9NO ($[M]^+$) 135.06787, found: 135.06766.

3-Fluoro-1-(pyridin-4-yl)prop-1-en-2-ol (2b)

4-Picoline (**1a**) (5.0 mmol, 0.50 ml), lithium diisopropylamide (5.5 mmol, 5.5 ml, 1.0 M in THF) and ethyl 2-fluoroacetate (5.5 mmol, 0.54 ml) resulted in 3-fluoro-1-(pyridin-4-yl)prop-1-en-2-ol (**2b**) and was purified via flash chromatography resulting in a red oil (0.726 g, 95%).

1H NMR (300 MHz, $CDCl_3$) δ = 3.83 (d, ^{4}J(H,H) = 1.7 Hz, 2H, CH_2), 4.81 (d, ^{2}J(H,F) = 47.6 Hz, 2H, CH_2F), 7.10 (d, ^{3}J(H,H) = 4.7 Hz, 2H, Hetar), 8.51 (d, ^{3}J(H,H) = 4.3 Hz, 2H. Hetar); 13C NMR (63 MHz, $CDCl_3$) δ = 44.5 (1C, CH_2), 84.7 (d, ^{1}J(C,F) = 186.3 Hz, 1C, CFH_2), 124.9 (2C, CH_{Hetar}), 141.2 (1C, C_{Hetar}), 145.0 (2C, CH_{Hetar}), 202.8 (d, ^{2}J(C,F) = 21.5 Hz, 1C, C=O); IR (ATR, cm^{-1}): $\tilde{\nu}$ = 3045, 2929, 1941, 1735, 1601, 1417, 1022, 568; MS (GC-MS): m/z (%) = 153 ($[M]^+$, 43), 120 (33), 93 (30), 92 (100), 65 (40), 64 (5), 63 (10), 61 (13), 52 (5), 51 (7), 50 (5), 39 (23), 33 (7) HRMS (EI): calcd. for C_8H_8FNO ($[M+H]^+$) 154.06627, found: 153.06623.

3,3-Difluor-1-(pyridin-4-yl)prop-1-en-2-ol (2c)

4-Picoline (**1a**) (5.0 mmol, 0.50 ml), lithium diisopropylamide (5.5 mmol, 5.5 ml, 1.0 M in THF) and ethyl 2,2-difluoroacetate (5.5 mmol, 0.50 ml) resulted in 3,3-difluor-1-(pyridin-4-yl)prop-1-en-2-ol (**2c**) and was purified via flash chromatography resulting in a light orange solid (0.719 g, 84%); mp: 115-116 °C.

1H NMR (500 MHz, Methanol-d4) δ = 3.35 (s, 1H, C*H*=C(OH)), 5.58 (t, ^2J(H,F) = 54.9 Hz, 1H, CF_2H), 7.38 (d, ^3J(H,H) = 6.0 Hz, 2H, Hetar), 8.40 (d, ^3J(H,H) = 5.7 Hz, 2H, Hetar); 19F NMR (282 MHz, Methanol-d4) δ = -136.5 (d, ^2J(F,F) = 284.1 Hz, 1F, CF_aF_bH), -132.5 (d, ^2J(F,F) = 285.1 Hz, 1F, CF_aF_bH); 13C NMR (126 MHz, Methanol-d4) δ = 50.0 (1C, *C*H=C(OH)), 97.1 (t, ^2J(C,F) = 23.8 Hz, 1C, C(OH)), 115.6 (t, ^1J(C,F) = 250.2 Hz, 1C, CF_2H), 128.1 (2C, CH_{Hetar}), 147.4 (1C, C_{Hetar}), 149.5 (2C, CH_{Hetar}); IR (ATR, cm^{-1}): $\tilde{\nu}$ = 3217, 3105, 3019, 2895, 2523, 2040, 1950, 1648, 1608, 1537, 1474, 1446, 1310, 1153, 1097, 1076, 1025, 988, 940, 824, 788, 764, 620; MS (GC-MS): m/z (%) = 171 ($[M]^+$, 25), 120 (32), 93 (8), 92 (100), 65 (38), 64 (5), (9), 51 (13), 39 (21) HRMS (EI): calcd. for $C_8H_7F_2NO$ ($[M+H]^+$) 172.05685, found: 172.05690.

3,3,3-Trifluoro-1-(pyridin-4-yl)prop-1-en-2-ol (2d)

4-Picoline (**1a**) (5.0 mmol, 0.50 ml), lithium diisopropylamide (5.5 mmol, 5.5 ml, 1.0 M in THF) and ethyl trifluoroacetate (5.5 mmol, 0.55 ml) resulted in 3,3,3-trifluoro-1-(pyridin-4-yl)prop-1-en-2-ol (**2d**) and was purified via flash chromatography resulting in a light yellow solid (0.765 g, 81%); decomposition at 168 °C.

1H NMR (250 MHz, DMSO-d6) δ = 5.40 (s, 1H, C*H*=C(OH)), 7.40 - 8.12 (m, 4H, Hetar), 12.69 (s, 1H, OH).
19F NMR (268 MHz, DMSO-d6) δ = -74.20 (s, 3F, CF_3); 13C NMR (75 MHz, DMSO-d6) δ = 88.3 (1C, *C*H=C(OH)), 116.5 (s, 2C, CH_{Hetar}), 118.8 (q, ^1J(C,F) = 292.7 Hz, 1C, CF_3), 137.0 (2C, CH_{Hetar}), 155.1 (1C, C_{Hetar}), 169.1 (q, ^2J(C,F) = 27.0 Hz, 1C, CH=*C*(OH)); IR (ATR, cm^{-1}): $\tilde{\nu}$ = 3228, 3026, 2902, 2624, 2041, 1950, 1876, 1776, 1647, 1438, 1476, 1454, 1403, 1270, 1229, 1159, 1104, 1088, 1050, 939, 879, 823, 719, 595, 536 MS (GC-MS): m/z (%) = 189 ($[M]^+$, 43), 120 (17), 93 (7), 92 (100), 69 (9), 65 (43), 64 (6), 63 (10), 52 (5), 51 (6), 50 (6), 39 (21); HRMS (EI): calcd. for $C_8H_6F_3NO$ ($[M+H]^+$) 190.04742, found: 190.04778; elemental analysis: calcd (%) for $C_8H_6F_3NO$ (189.13): C, 50.80; H, 3.20; N, 7.41. Found: C, 50.65; H, 3.07; N, 7.33.

1-(Pyridin-2-yl)propan-2-one (2e)

2-Picoline (**1b**) (20.0 mmol, 1.98 ml), *n*-butyllithium solution (22.0 mmol, 8.88 ml, 2.5 M) and dimethylacetamide (19.0 mmol, 1.25 ml) resulted in 1-(pyridin-2-yl)propan-2-on (**2e**) and was purified via flash chromatography resulting in a yellow oil (2.375 g, 88%, keto/enol 90/10 in $CHCl_3$).

^{1}H NMR (300 MHz, $CDCl_3$) ketone δ = 2.22 (s, 3H, CH_3), 3.91 (s, 2H, CH_2), 7.18 (dd, ^{3}J(H,H) = 7.7, ^{4}J(H,H) = 4.9 Hz, 1H, Hetar), 7.20 (d, ^{3}J(H,H) = 7.7 Hz, 1H, Hetar), 7.64 (ddd, ^{3}J(H,H) = 7.7(H,H), ^{3}J(H,H) = 7.7(H,H), ^{4}J(H,H) = 1.8 Hz, 1H, Hetar), 8.55 (d, ^{3}J(H,H) = 4.7 Hz, 1H, Hetar); enol δ = 2.01 (s, 3H, CH_3), 5.29 (s, 1H, C*H*=C(OH)), 6.85 (dd, ^{3}J(H,H) = 7.1, ^{4}J(H,H) = 5.5 Hz, 1H, Hetar), 6.88 (d, ^{3}J(H,H) = 7.5 Hz, 1H, Hetar), 7.52 (ddd, ^{3}J(H,H) = 7.8, ^{3}J(H,H) = 7.8, ^{4}J(H,H) = 1.7 Hz, 1H, Hetar), 8.18 (d, ^{3}J(H,H) = 5.1 Hz, 1H, Hetar), 14.8 (s, 1H, O*H*); 13C NMR (75 MHz, $CDCl_3$) ketone δ = 29.9 (1C, CH_3), 53.1 (1C, CH_2), 121.9, 124.1, 136.6, 149.5 (1C, CH_{Hetar}), 154.7 (1C, C_{Hetar}), 205.3 (1C, *C*=O); enole δ = 24.3 (1C, CH_3), 95.4 (1C, *C*H=C(OH)), 120.6, 123.2, 136.2, 148.9 (1C, CH_{Hetar}), 158.2 (1C, C_{Hetar}), 170.6 (1C, *C*H(OH)); IR (ATR, cm^{-1}): $\tilde{\nu}$ = 3407, 3051, 3008, 2955, 2918, 1711, 1589, 1434, 751, 540; MS (GC-MS): m/z (%) = 136 ($[M+H]^+$, 2), 135 ($[M]^+$, 2), 94 (7), 93 (100), 92 (20), 67 (6), 66 (7), 65 (19), 64 (7), 63 (15), 62 (8), 52 (7), 51 (13), 50 (12), 43 (73), 42 (9), 39 (33), 38 (11);
HRMS (EI): calcd. for C_8H_9NO ($[M]^+$) 135.06787, found: 135.06797.

1-Fluoro-3-(pyridin-2-yl)propan-2-one (2f)

2-Picoline (**1b**) (2.0 mmol, 1.98 ml), *n*-butyllithium solution (2.2 mmol, 0.88 ml, 2.5 M) and ethyl 2-fluoroacetat (1.9 mmol, 0.20 ml) resulted in 1-fluoro-3-(pyridin-2-yl)propan-2-one (**2f**) and was purified via purification via flash chromatography resulting in a yellow oil (0.242 g, 95%, keto/enol 40/60).

^{1}H NMR (300 MHz, $CDCl_3$) ketone δ = 4.03 (d, ^{4}J(H,F) = 3.0 Hz, 2H, CH_2), 4.87 (d, ^{2}J(H,F) = 47.0 Hz, 2H, CH_2F), 7.20 (dd, ^{3}J(H,H) = 7.6, ^{3}J(H,H) = 5.3 Hz, 1H, Hetar), 7.24 (d, ^{3}J(H,H) = 7.9 Hz, 1H, Hetar), 7.59 (ddd, ^{3}J(H,H) = 8.1, ^{3}J(H,H) = 7.4, ^{2}J(H,H) = 1.7 Hz, 1H, Hetar), 8.54 (d, ^{3}J(H,H) = 4.9 Hz, 1H, Hetar); enol δ = 4.98 (d, ^{2}J(H,F) = 47.4 Hz, 2H, CH_2F), 5.61 (s, 1H, C*H*=C(OH)), 6.94 (ddd, ^{3}J(H,H) = 7.4, ^{3}J(H,H) = 5.7, ^{4}J(H,H) = 1.3 Hz, 1H, Hetar), 6.97 (d, ^{3}J(H,H) = 8.3 Hz, 1H, Hetar), 7.67 (ddd, ^{3}J(H,H) = 7.7, ^{3}J(H,H) =7.7, ^{4}J(H,H) = 1.8 Hz, 1H, Hetar), 8.12 (d, ^{3}J(H,H) = 5.3 Hz, 1H, Hetar); 13C NMR (75 MHz, $CDCl_3$) ketone δ = 47.9 (1C, CH_2), 84.8 (d, ^{1}J(C,F) = 185.4 Hz, 1C, CH_2F), 122.2, 124.2, 136.8, 149.5 (1C, CH_{Hetar}), 157.3 (1C, C_{Hetar}), 202.9 (d, ^{2}J(C,F) = 19.3 Hz, 1C, *C*=O); enol δ = 82.0 (d, ^{1}J(C,F) = 173.3 Hz, 1C, CH_2F), 93.1 (d, ^{3}J(C,F) = 7.7 Hz, 1C, *C*H=C(OH)), 118.0, 121.3, 137.6, 142.1 (1C, CH_{Hetar}), 153.4 (d, ^{4}J(C,F) = 1.1 Hz, 1C, C_{Hetar}), 167.5 (d, ^{2}J(C,F) = 17.6 Hz, 1C, CH=*C*(OH)); IR (ATR, cm^{-1}): $\tilde{\nu}$ = 3056, 3010, 2934, 1735, 1589, 1567, 1434, 1240, 1151, 1030, 922, 743; MS (GC-MS): m/z (%) = 153 ($[M]^+$, 24), 121 (5), 120 (55), 93 (13), 92 (100), 66 (7), 65 (47), 64 (8), 63 (12), 62 (5), 52 (9), 51 (10), 39 (16), 33 (7); HRMS (EI): calcd. for C_8H_8FNO ($[M]^+$) 153.05844, found: 153.05882.

3,3-Difluoro-1-(pyridin-2-yl)prop-1-en-2-ol (2g)

2-Picoline (**1b**) (2.0 mmol, 1.98 ml), *n*-butyllithium solution (2.2 mmol, 0.88 ml, 2.5 M) and ethyl 2,2-difluoroacetat (1.9 mmol, 0.20 ml) resulted in 3,3-difluoro-1-(pyridin-2-yl)prop-1-en-2-ol (**2g**) and was purified via flash chromatography resulting in a yellow solid (0.242 g,72%); mp: 50-51 °C.

1H NMR (300 MHz, $CDCl_3$) δ = 5.79 (s, 1H, C*H*=C(OH)), 6.05 (t, ^2J(H,F) = 55.0 Hz, 1H, CF_2H), 7.08 (d, ^3J(H,H) = 8.3 Hz, 1H, Hetar), 7.06 (dd, ^3J(H,H) = 7.7, ^3J(H,H) = 6.0 Hz, 1H, Hetar), 7.68 (ddd, ^3J(H,H) = 8.2, ^3J(H,H) = 7.5, ^4J(H,H) = 1.7 Hz, 1H, Hetar), 8.20 (d, ^3J(H,H) = 5.5 Hz, 1H, Hetar), 14.91 (s, 1H, OH); 19F NMR (282 MHz, $CDCl_3$) δ = -123.87 (s, 2F, CF_2H);13C NMR (75 MHz, $CDCl_3$) δ = 93.5 (t, ^3J(C,F) = 5.5 Hz, 1C, C*H*=C(OH)), 111.1 (t, ^1J(C,F) = 243.2 Hz, 1C, CF_2H), 119.0, 122.2, 138.2, 142.1 (1C, CH_{Hetar}), 156.7 (1C, C_{Hetar}), 162.8 (t, ^2J(C,F) = 22.6 Hz, 1C, *C*(OH)); IR (ATR, cm^{-1}): $\tilde{\nu}$ = 3107, 3060, 2967, 2917, 2849, 1985, 1900, 1856, 1578, 1432, 1318, 1152, 1069, 1037, 995, 864, 762, 725, 641, 536; MS (GC-MS): m/z (%) = 171 ($[M]^+$, 25), 124 (8), 121 (7), 120 (73), 93 (8), 92 (100), 78 (8), 65 (39), 64 (8), 63 (10), 62 (5), 52 (6), 51 (18), 50 (5), 39 (15), 38 (6); HRMS (ESI-TOF): calcd. for $C_8H_7F_2NO$ ($[M+H]^+$) 172.05685, found: 172.05682.

3,3,3-Trifluoro-1-(pyridine-2-yl)prop-1-en-2-ol (2h)

2-Picoline (**1b**) (3.0 mmol, 0.30 ml), pyridine (15.0 mmol, 1.21 ml) and trifluoroacetanhydrid (9.0 mmol, 1.27 ml) resulted in 3,3,3-trifluoro-1-(pyridine-2-yl)prop-1-en-2-ol (**2h**) and was purified via flash chromatography resulting in a yellow solid (0.346 g, 64%); mp: 108-109 °C.

^{1}H NMR (300 MHz, DMSO-*d6*) δ = 5.75 (s, 1H, C*H*=C(OH)), 7.11 (ddd, ^3J (H,H)= 7.0, ^3J(H,H) = 6.2, ^4J(H,H) = 0.9 Hz, 1H, Hetar), 7.35 (d, ^3J(H,H) = 7.2 Hz, 1H, Hetar), 7.89 (ddd, ^3J(H,H) = 8.6, ^3J(H,H) = 7.1, ^4J(H,H) = 1.6 Hz, 1H, Hetar), 8.24 (d, ^3J(H,H) = 6.0 Hz, 1H, Hetar), 15.36 (br. s, 1H, O*H*). ^{19}F NMR (282 MHz, DMSO-*d6*) δ = -74.10 (s, 3F, CF_3). ^{13}C NMR (63 MHz, DMSO-*d6*) δ = 85.03 (1C, *C*H=C(OH)), 116.90 (1C, CH_{Hetar}), 118.80 (q, ^1J(C,F) = 286.1 Hz, 1C, CF_3), 122.19, 137.42, 140.90 (1C, CH_{Hetar}), 154.35 (1C, C_{Hetar}), 165.94 (q, ^2J(C,F) = 31.1 Hz, 1C, *C*(OH)CF_3). MS (GC-MS): m/z (%) = 189 ($[M]^+$, 26), 121 (7), 120 (82), 92 (100), 91 (5), 65 (37), 64 (7), 63 (9), 51 (6), 39 (10). HRMS (EI): calc. for $C_8H_6F_3NO$ ($[M]^+$) 189.03960, found: $C_8H_6F_3NO$ ($[M]^+$) 189.03958. CH-Analysis: Calc. for $C_8H_6F_3NO$ (189.13): C, 50.80; H, 3.20; N, 7.41, found: C, 50.85; H, 3.13; N, 7.40.

3-Chloro-3,3-difluor-1-(pyridin-2-yl)prop-1-en-2-ol (2i)

2-Picoline (**1b**) (2.0 mmol, 1.98 ml), *n*-butyllithium solution (2.2 mmol, 0.88 ml, 2.5 M) and methyl 2-chloro-2,2-difluoroacetate (1.9 mmol 0.20 ml) resulted in 3-chloro-3,3-difluor-1-(pyridin-2-yl)prop-1-en-2-ol (**2i**) and was purified via flash chromatography resulting in a yellow solid (0.344 g, 84%); mp: 101-102 °C.

1H NMR (250 MHz, $CDCl_3$) δ = 5.78 (s, 1H, C*H*=C(OH)), 6.99 (ddd, ^3J(H,H) = 7.3, ^3J(H,H) = 6.0, ^3J(H,H) = 1.1 Hz, 1H, Hetar), 7.08 (d, ^3J(H,H) = 8.5 Hz, 1H, Hetar), 7.69 (ddd, ^3J(H,H) = 8.6, ^3J(H,H) = 7.2, ^4J(H,H) = 1.6 Hz, 1H, Hetar), 8.05 (d, ^3J(H,H) = 6.0 Hz, 1H, Hetar), 15.80 (s, 1H, O*H*); ^{19}F NMR (235 MHz, $CDCl_3$) δ = -61.97 (s, 2F, CF_2Cl); 13C NMR (75 MHz, $CDCl_3$) δ = 86.9 (t, ^3J(C,F) = 3.6 Hz, 1C, *C*H=C(OH)), 117.2 (1C, CH_{Hetar}), 122.5 (t, ^1J(C,F) = 296.0 Hz, 1C, CF_2Cl), 122.7 (1C, CH_{Hetar}), 137.9, 139.3 (1C, CH_{Hetar}), 155.9 (1C, C_{Hetar}), 168.0 (t, ^3J(C,F) = 27.5 Hz, 1C, C(OH)); IR (ATR, cm^{-1}): $\tilde{\nu}$ = 3109,

3063, 1638, 1587, 1429, 1078, 943, 768, 699, 622; MS (GC-MS): m/z (%) = 207 ([M ^{37}Cl]$^+$ 4), 205 ([M ^{35}Cl]$^+$ 11), 170 (14), 142 (10),121 (9), 120 (79), 93 (13), 92 (100), 65 (37), 64 (8), 63 (8), 52 (10), 51 (9), 39 (8), 38 (5); HRMS (EI): calcd. for $C_8H_6{}^{35}ClF_2NO$ ([M]$^+$) 205.01005, found: 205.00990. calcd. for $C_8H_6{}^{37}ClF_2NO$ ([M]$^+$) 207.00710, found: 207.00728.

1-(6-Methylpyridin-2-yl)propan-2-one (2j)

2,6-Lutidine (**1c**) (20.0 mmol, 2.33 ml), *n*-butyllithium solution (22.0 mmol, 8.88 ml, 2.5 M) and dimethylacetamide (19.0 mmol, 1.25 ml) resulted in 1-(6-methylpyridin-2-yl)propan-2-on (**2j**) and was purified via flash chromatography resulting in a red oil (2.628 g, 90%, keto/enol 90/10).

1H NMR (300 MHz, $CDCl_3$) ketone δ = 2.21 (s, 3H, CH_3), 2.53 (s, 3H, CH_3), 3.88 (s, 2H, CH_2), 7.00 (d, ^3J(H,H) = 7.7 Hz, 1H, Hetar), 7.03 (d, ^3J(H,H) = 7.7 Hz, 1H, Hetar), 7.5 (dd, ^3J(H,H) = 7.6, ^3J(H,H) = 7.6 Hz, 1H, Hetar); enol δ = 2.02 (s, 3H, CH_3), 2.45 (s, 3H, CH_3), 5.28 (s, 1H, C*H*=C(OH)), 6.65 (d, ^3J(H,H) = 8.1 Hz, 1H, Hetar), 6.70 (d, ^3J(H,H) = 7.6 Hz, 1H, Hetar), 7.41 (dd, ^3J(H,H) = 7.7, ^3J(H,H) = 7.7 Hz, 1H, Hetar), 15.3 (s, 1H, O*H*); 13C NMR (75 MHz, $CDCl_3$) keto-Form δ = 24.4, 29.8 (1C, *C*H_3), 53.2 (1C, *C*H_2), 120.9, 121.5, 136.8 (1C, CH_{Hetar}), 153.9, 158.3 (1C, C_{Hetar}), 205.5 (1C, *C*=O); enol δ = 22.9, 23.0 (1C, *C*H_3), 94.6 (1C, *C*H=C(OH)), 116.8, 117.1, 137.3 (1C, CH_{Hetar}), 152.3, 157.8 (1C, C_{Hetar}), 169.1 (1C, *C*(OH)); IR (ATR, cm^{-1}): $\tilde{\nu}$ = 3399, 3062, 3000, 2957, 2920, 1712, 1454, 1157, 777, 531; MS (GC-MS): m/z (%) = 149 ([M]$^+$), 108 (8), 107 (100), 106 (16), 79 (6), 77 (8), 66 (6), 43 (17), 39 (6); HRMS (EI): calcd. for $C_9H_{11}NO$ ([M]$^+$) 149.08352, found: $C_9H_{11}NO$ ([M]$^+$) 149.08353.

3,3,3-Trifluor-1-(6-methylpyridin-2-yl)prop-1-en-2-ol (2k)

2,6-Lutidine (**1c**) (10.0 mmol, 0.35 ml), *n*-butyllithium solution (11.0 mmol, 4.40 ml, 2.5 M) and methyl trifluoroacetat (15.0 mmol, 1.50 ml) resulted in 3,3,3-trifluor-1-(6-methylpyridin-2-yl)prop-1-en-2-ol (**2k**) and was purified via flash chromatography resulting in a yellow solid (0.182 g, 90%).

1H NMR (300 MHz, $CDCl_3$) δ = 2.54 (s, 3H, CH_3), 5.77 (s, 1H. C*H*=C(OH)), 6.82 (d, ^3J(H,H) = 7.4 Hz, 1H, Hetar), 6.90 (d, ^3J(H,H) = 8.3 Hz, 1H, Hetar), 7.62 (dd, ^3J(H,H) = 7.9, ^3J(H,H) = 7.9 Hz, 1H, Hetar), 16.10 (s, 1H, O*H*); 19F NMR (282 MHz, $CDCl_3$) δ = -75.27 (s, 3F, CF_3); 13C NMR (63 MHz, $CDCl_3$) δ = 20.9 (1C, *C*H_3), 88.2 (q, ^3J(C,F) = 2.9 Hz, 1C, *C*H=C(OH)), 117.2 (1C, CH_{Hetar}), 119.0 (q, ^1J(C,F) = 283.4 Hz, 1C, *C*F_3), 119.2 (1C, CH_{Hetar}), 139.9 (1C, CH_{Hetar}), 148.0, 155.7 (1C, C_{Hetar}), 165.2 (q, ^2J(C,F) = 33.4 Hz, 1C, *C*(OH)); IR (ATR, cm^{-1}): $\tilde{\nu}$ = 3389, 3256, 1671, 1459, 1187, 1128, 842, 799, 723; MS (GC-MS): m/z (%) = 203 ([M]$^+$, 37), 156 (7), 135 (7), 134 (89), 107 (9), 106 (100), 104 (9), 79 (30), 78 (12), 77 (27), 69 (11), 63 (12), 62 (5), 53 (5), 52 (8), 51 (5), 50 (5), 39 (11), 38 (6); HRMS (EI): calcd. for $C_9H_8F_3NO$ ([M]$^+$) 203.05525, found: 203.05542; Elemental analysis: calcd. for $C_9H_8F_3NO$ (203.16): C, 53.21; H, 3.97; N, 6.89, found: C, 52.74; H, 3.87; N, 6.79.

1-(4,6-Dimethylpyridin-2-yl)propan-2-one (2l)

2,4,6-Trimethylpyridine (**1d**) (10.0 mmol, 1.32 ml), *n*-butyllithium solution (11.0 mmol, 4.40 ml, 2.5 M) and dimethylacetamide (15.0 mmol 1.00 ml) resulted in 1-(4,6-dimethylpyridin-2-yl)propan-2-on(**2l**) and was purified via flash chromatography resulting in an orange oil (1.330 g, 82%, keto/enol 87/13).

1H NMR (300 MHz, $CDCl_3$) ketone δ = 2.21 (s, 3H, CH_3), 2.28 (s, 3H, CH_3), 2.49 (s, 3H, CH_3), 3.84 (s, 2H, CH_2), 6.83 (s, 1H Hetar), 6.87 (s, 1H Hetar); enol δ = 2.01 (s, 3H, CH_3), 2.22 (s, 3H, CH_3), 2.40 (s, 3H, CH_3), 5.20 (s, 1H, C*H*=C(OH)), 6.46 (s, 1H, Hetar), 6.52 (s, 1H, Hetar), 15.56 (s, 1H, O*H*); 13C NMR (75 MHz, $CDCl_3$) ketone δ = 20.8, 24.1, 29.9 (1C, CH_3), 53.1 (1C, CH_2), 122.0, 122.6 (1C, CH_{Hetar}), 148.0, 153.6, 157.9 (1C, C_{Hetar}), 205.7 (1C, *C*=O); enol δ = 21.1, 22.5, 23.3 (1C, CH_3), 93.9 (1C, *C*H=C(OH)), 117.3, 117.9 (1C, CH_{Hetar}), 148.6, 151.2, 157.4 (1C, C_{Hetar}), 170.5 (1C, *C*(OH)); IR (ATR, cm^{-1}): $\tilde{\nu}$ = 3393, 2954, 2920, 2857, 1712, 1607, 1354, 1156, 848, 528; MS (GC-MS): m/z (%) = 163 ($[M]^+$, 5), 162 (6), 148 (7), 134 (5), 122 (16), 121 (100), 120 (21), 119 (12), 118 (14), 117 (7), 105 (7), 194 (17), 103 (6), 102 (5), 91 (12), 80 (7), 79 (14), 78 (26), 77 (48), 66 (10), 64 (7), 53 (12), 52 (6), 51 (6), 50 (7), 49 (6), 43 (78), 42 (31), 41 (15), 40 (28), 39 (23); HRMS (EI): calcd. for $C_{10}H_{13}NO$ ($[M]^+$) 163.09917, found: 163.09938.

3,3,3-Trifluor-1-(4,6-dimethylpyridin-2-yl)prop-1-en-2-ol (2m)

2,4,6-Trimethylpyridine (**1d**) (10.0 mmol, 1.32 ml), *n*-butyllithium solution (11.0 mmol, 4.40 ml, 2.5 M) and methyl trifluoroacetat (15.0 mmol, 1.50 ml) resulted in 3,3,3-trifluor-1-(4,6-dimethylpyridin-2-yl)prop-1-en-2-ol (**2m**) and was purified by recrystallization from *n*-heptane/dichloromethane resulting in a yellow solid (1.986 g, 91%); mp: 96-97 °C.

1H NMR (300 MHz, $CDCl_3$) δ = 2.33 (s, 3H, CH_3), 2.48 (s, 3H, CH_3), 5.64 (s, 1H, C*H*=C(OH)), 6.62 (s, 1H, Hetar), 6.68 (s, 1H, Hetar), 16.16 (s, 1H, O*H*); 19F NMR (282 MHz, $CDCl_3$) δ = -75.46 (s, 3F, CF_3); 13C NMR (63 MHz, $CDCl_3$) δ = 20.3 (1C, CH_3), 21.6 (1C, CH_3), 86.6 (q, ^3J(C,F) = 3.1 Hz, 1C, *C*H=C(OH)), 118.4, 118.8 (1C, CH_{Hetar}), 119.0 (q, ^1J(C,F) = 282.9 Hz, 1C, CF_3), 146.3, 152.4, 155.3 (1C, C_{Hetar}), 166.6 (q, ^2J(C,F) = 33.0 Hz, 1C, *C*(OH)); IR (ATR, cm^{-1}): $\tilde{\nu}$ = 3269, 3103, 3086, 3037, 3019, 1626, 1588, 1243, 1164, 1104, 874, 799, 719, 712, 554, 547; MS (GC-MS): m/z (%) = 217 ($[M]^+$, 55), 149 (10), 148 (96), 121 (9), 120 (100), 118 (7), 104 (7), 93 (20), 91 (13), 79 (6), 78 (6), 77 (23), 69 (5), 53 (5), 51 (6), 39 (6); HRMS (EI): calcd. for $C_{10}H_{10}F_3NO$ ($[M]^+$) 217.07090, found: 217.07071.

3. Racemic alcohols (rac-2a-2c and rac-2e-2g)

Representative procedure for *rac*emic reduction of pro-chiral ketones

In a Schlenk flask (under argon) the corresponding ketone (1 eq.) was dissolved in dry methanol (3 M^{-1}). At room temperature sodium borohydride was added in portions (beware of strong gas formation) and monitored by TLC. If full conversion was not achieved within one hour another half equivalent of sodium borohydride was added in portions. Afterwards the crude reaction mixture was carefully mixed with aqueous 5% HCl-solution, extracted three times with 15 ml of ethylacetate and the combined organic phase dried with sodium sulfate overnight. The crude product was obtained after evaporation of all volatile compounds and further purified by column chromatography.

(*rac*)-1-(Pyridin-4-yl)propan-2-ol (rac-3a)

1-(Pyridin-4-yl)propan-2-one (**2a**) (1.0 mmol, 135 mg) and sodium borohydride (1.0 mmol, 37 mg) were dissolved in 3.00 ml dry methanol result in (*rac*)-1-(pyridin-4-yl)propan-2-ol (**3a**) and was purified via flash chromatography resulting in a yellow oil (0.097 g, 71%).
1H NMR (300 MHz, $CDCl_3$) δ = 1.16 (d, ^3J(H,H) = 6.2 Hz, 3H, CH_3), 2.49 - 2.82 (m, 2H, C*H*2CH(OH)), 3.90 - 4.09 (m, 1H, C*H*(OH)CH3), 4.35 (s, 1H, OH), 7.07 (d, ^3J(H,H) = 5.1 Hz, 2H, Hetar), 8.28 (d, ^3J(H,H) = 3.8 Hz, 2H Hetar); 13C NMR (75 MHz, $CDCl_3$) δ= 23.1 (1C, CH_3), 44.8 (1C, CH_2), 67.3 (1C, *C*H(OH)), 124.6 (2C, CH_{Hetar}), 148.6 (1C, C_{Hetar}), 148.7 (2C, CH_{Hetar}); IR (ATR, cm^{-1}): $\tilde{v}$ = 3239, 3076, 2968, 2928, 2928, 1604, 1418, 1117, 1002, 939, 791, 604. MS (GC-MS): m/z (%) = 137 ($[M]^+$, 1), 94 (10), 93 (100), 92 (13), 67 (5), 66 (7), 65 (11), 63 (5), 51 (8), 45 (21), 43 (5), 39 (14); HRMS (EI): calcd. for $C_8H_{11}NO$ ($[M]^+$) 137.08352, found: $C_8H_{11}NO$ ($[M]^+$) 137.08385.

(*rac*)-1-Fluor-3-(pyridin-4-yl)propan-2-ol (rac-3b)

1-Fluor-3-(pyridin-4-yl)propan-2-one (**2b**) (1.0 mmol, 153 mg) and sodium borohydride (1.0 mmol, 37 mg) in 3.00 ml dry methanol result in (*rac*)-1-Fluor-3-(Pyridin-4-yl)propan-2-ol (**3b**) and was purified via flash chromatography resulting in a yellow oil (0.124 g, 80%).
1H NMR (300 MHz, $CDCl_3$) δ = 2.85 (dd, ^2J(H,H) = 13.8, ^3J(H,H) = 5.1 Hz, 1H, CH^aH^b), 2.78 (dd, ^2J(H,H) = 13.8, ^3J(H,H) = 8.1 Hz, 1H, CH^aH^b), 3.94 (s, 1H, OH), 4.05 - 4.20 (m, 1H, C*H*(OH)), 4.38 (ddd, ^2J(H,F) = 47.0, ^2J(H,H) = 20.4, ^3J(H,H) = 9.4 Hz, 1H, CH^aH^bF), 4.40 (ddd, ^2J(H,F) = 47.0, ^2J(H,H) = 18.7, ^3J(H,H) = 9.4 Hz, 1H, CH^aH^bF), 7.19 (d, ^3J(H,H) = 5.1 Hz, 2H, Hetar), 8.40 (s, 2H, Hetar); 13C NMR (75 MHz, $CDCl_3$) δ = 38.3 (d, ^3J(C,F) = 6.1 Hz, 1C, CH_2), 70.1 (d, ^2J(C,F) = 19.8 Hz, 1C, *C*H(OH)), 85.8 (d, ^1J(C,F) = 170.6 Hz, 1C, CFH_2), 124.9 (2C, CH_{Hetar}), 147.4 (1C, C_{Hetar}), 149.2 (2C, CH_{Hetar}); IR (ATR, cm^{-1}): $\tilde{v}$ = 3163, 3076, 2952, 2895, 1941, 1604, 1417, 1220, 1105, 1069, 1003, 793, 555; MS (GC-MS): m/z (%) = 155 ($[M]^+$, 18), 122 (5), 94 (14), 93 (100), 92 (16), 67 (6), 66 (8), 65 (12), 63 (19), 51 (8), 43 (7), 39 (12); HRMS (EI): calcd. for $C_8H_{10}FNO$ ($[M]^+$) 155.07409, found: $C_8H_{10}FNO$ ($[M]^+$) 155.07390.

(*rac*)-1,1-Difluor-3-(pyridin-4-yl)propan-2-ol (rac-3c)

3,3-Difluor-1-(pyridin-4-yl)propan-2-one (**2c**) (1.0 mmol, 171 mg) and sodium borohydride (1.0 mmol, 37 mg) in 3.00 ml dry methanol result in (*rac*)-1,1-difluor-3-(pyridin-4-yl)propan-2-ol (**3c**) and was purified via flash chromatography resulting in a light yellow oil (0.147 g, 85%).
1H NMR (300 MHz, $CDCl_3$) δ = 2.75 (dd, $^2J(H,H)$ = 14.0, $^3J(H,H)$ = 9.6 Hz, 1H, CH^aH^b), 2.91 (dd, $^2J(H,H)$ = 14.0, $^3J(H,H)$ = 3.0 Hz, 1H, CH^aH^b), 3.82 - 4.07 (m, 1H, CH(OH)), 5.68 (t, $^2J(H,F)$ = 56.6, $^3J(H,H)$ = 4.1 Hz, 1H, CHF_2), 7.17 (d, $^3J(H,H)$ = 4.9 Hz, 2H, Hetar), 8.21 (s, 2H, Hetar). 19F NMR (282 MHz, $CDCl_3$) δ = -127.02 (d, $^2J(F,F)$ = 286.1 Hz, 1F, CF^aF^bH), -124.80 (d, $^2J(F,F)$ = 286.1 Hz, 1F, CF^aF^bH); 13C NMR (75 MHz, $CDCl_3$/Methanol-d4) δ = 35.4 (t, $^3J(C,F)$ = 3.9 Hz, 1C, CH_2), 70.3 (t, $^2J(C,F)$ = 23.3 Hz, 1C, CH(OH)), 115.8 (t, $^1J(C,F)$ = 244.4 Hz, 1C, CF_2H), 125.1 (2C, CH_{Hetar}), 147.7 (1C, C_{Hetar}), 148.5 (2C, CH_{Hetar}); IR (ATR, cm^{-1}): $\tilde{v}$ = 3079, 2972, 2907, 2754, 1942, 1732, 1607, 1561, 1419, 1183, 1092, 1048, 1005, 837, 800, 538; MS (GC-MS): m/z (%) = 174 ($[M+H]^+$, 6), 173 ($[M]^+$, 71), 122 (44), 94 (38), 93 (100), 92 (43), 81 (6), 78 (6), 67 (9), 66 (13), 65 (32), 63 (7), 61 (11), 52 (7), 51 (23), 50 (7), 41 (6), 39 (23), 33 (5); HRMS (EI): calcd. for $C_8H_9F_2NO$ ($[M+H]^+$) 174.0725, found: $C_8H_9F_2NO$ ($[M+H]^+$) 174.07255.

(*rac*)-1-(Pyridin-2-yl)propan-2-ol (rac-3e)

1-(Pyridin-2-yl)propan-2-one (**2e**) (1.0 mmol, 135 mg) and sodium borohydride (1.0 mmol, 37 mg) in 3.00 ml dry methanol result in (*rac*)-1-(pyridin-2-yl)propan-2-ol (**3e**) and was purified via flash chromatography resulting in a light brown oil (0.124 g, 90%).

1H NMR (300 MHz, $CDCl_3$) δ = 1.29 (d, $^3J(H,H)$ = 6.2 Hz, 3H, CH_3), 2.86 (dd, $^2J(H,H)$ = 14.9, $^3J(H,H)$ = 8.1 Hz, 1H, CH^aH^b), 2.93 (dd, $^2J(H,H)$ = 15.0, $^3J(H,H)$ = 3.3 Hz, 1H, CH^aH^b), 4.25 (m, 1H, *CH*(OH)), 4.48 (s, 1H, OH), 7.18 (dd, $^3J(H,H)$ = 5.3, $^3J(H,H)$ = 5.3 Hz, 1H, Hetar), 7.16 (d, $^3J(H,H)$ = 8.1 Hz, 1H, Hetar), 7.65 (ddd, $^3J(H,H)$ = 7.7, $^3J(H,H)$ = 7.7, $^4J(H,H)$ = 1.8 Hz, 1H, Hetar), 8.51 (d, $^3J(H,H)$ = 4.2 Hz, 1H, Hetar); 13C NMR (63 MHz, $CDCl_3$) δ = 23.0 (1C, CH_3), 44.8 (1C, CH_2), 67.1 (1C, CH(OH)), 121.6, 123.7, 136.9, 148.3 (1C, CH_{Hetar}), 160.1 (1C, C_{Hetar}); IR (ATR, cm^{-1}): $\tilde{v}$ = 3281, 3013, 2966, 2927, 2872, 1594, 1435, 1117, 939, 756, 613; MS (GC-MS): m/z (%) = 137 ($[M]^+$ 0.4), 136 (4), 122 (10), 94 (13), 93 (100), 92 (12), 80 (14), 78 (6), 66 (13), 65 (14), 52 (5), 45 (8), 39 (11); HRMS (ESI-TOF): calcd. for $C_8H_{11}NO$ ($[M+H]^+$) 138.09134, found: $C_8H_{11}NO$ ($[M+H]^+$) 138.09316.

(*rac*)-1-Fluor-3-(pyridin-2-yl)propan-2-ol (rac-3f)

1-Fluor-3-(pyridin-2-yl)propan-2-one (**2f**) (1.0 mmol, 135 mg) and sodium borohydride (1.0 mmol, 37 mg) in 3.00 ml dry methanol result in (*rac*)-1-fluor-3-(pyridin-2-yl)propan-2-ol (**3f**) and was purified via flash chromatography resulting in a light brown oil (0.124 g, 81%).

1H NMR (300 MHz, $CDCl_3$) δ = 2.99 (dd, ^2J(H,H) = 14.9, 3J = 7.0 Hz, 1H, C$H^a$$H^b$), 3.04 (dd, ^2J(H,H) = 14.9, ^3J(H,H) = 4.3 Hz, 1H, C$H^a$$H^b$), 4.22 - 4.36 (m, 1H, CH(OH)), 4.42 (ddd, ^2J(H,F) = 46.6, ^2J(H,H) = 18.3, ^3J(H,H) = 9.3 Hz, 1H, C$H^a$$H^b$F), 4.44 (ddd, ^2J(H,F) = 47.2, ^2J(H,H) = 18.7, ^3J(H,H) = 9.3 Hz, 1H, C$H^a$$H^b$F), 4.77 (s, 1H, OH), 7.20 (d, ^3J(H,H) = 7.6 Hz, 1H, Hetar), 7.20 (dd, ^3J(H,H) = 6.9, ^3J(H,H) = 6.9 Hz, 1H, Hetar), 7.66 (ddd, ^3J(H,H) = 7.6, ^3J(H,H) = 7.6, ^4J(H,H) = 1.7 Hz, 1H, Hetar), 8.49 (d, ^3J(H,H) = 4.2 Hz, 1H); 13C NMR (63 MHz, $CDCl_3$) δ = 38.6 (d, ^3J(C,F) = 5.5 Hz, 1C, CH_2), 69.5 (d, ^2J(C,F) = 20.6 Hz, 1C, CH(OH)), 85.7 (d, ^1J(C,F) = 170.7 Hz, 1C, CH_2F), 121.8, 123.9, 137.0, 148.5 (1C, CH_{Hetar}), 159.0 (1C, CHetar); IR (ATR, cm^{-1}): $\tilde{v}$ = 3215, 3012, 2951, 2895, 1594, 1569, 1476, 1436, 1103, 1001, 757, 562, 554; MS (GC-MS): m/z (%) = 155 ($[M]^+$, 0.03), 122 (16), 117 (15), 94 (10), 93 (100), 92 (9), 90 (6), 89 (6), 78 (6), 66 (10), 65 (9), 63 (6), 51 (6), 39 (7); HRMS (ESI-TOF): calcd. for $C_8H_{10}FNO$ ($[M+H]^+$) 156.08192, found: $C_8H_{10}FNO$ ($[M+H]^+$) 156.08192.

1,1-Difluor-3-(pyridin-2-yl)propan-2-ol (rac-3g)

1,1-Difluor-3-(pyridin-2-yl)prop-1-en-2-ol (**2g**) (1.0 mmol, 171 mg) and sodium borohydride (1.0 mmol, 37 mg) in 3.00 ml dry methanol result in (*rac*)-1,1,1-difluor-3-(pyridin-2-yl)propan-2-ol (**3g**) and was purified via flash chromatography resulting in a light brown oil (0.171 g, 99%).

1H NMR (250 MHz, $CDCl_3$) δ = 3.01 (dd, ^2J(H,H) = 15.3, ^3J(H,H) = 8.4 Hz, 1H, C$H^a$$H^b$), 3.09 (dd, ^2J(H,H) = 15.6, ^3J(H,H) = 3.5 Hz, 1H, C$H^a$$H^b$), 4.00 - 4.36 (m, 1H, CH(OH)), 5.26 (s, 1H, OH), 5.75 (td, ^2J(H,F) = 56.0, ^3J(H,H) = 3.9 Hz, 1H, CF_2H), 7.20 (dd, ^3J(H,H) = 7.1 Hz, 1H, Hetar), 7.21 (d, ^3J(H,H) = 6.9 Hz, 2H, Hetar), 7.66 (dd, ^3J(H,H) = 7.4, ^3J(H,H) = 7.4 Hz, 1H, Hetar), 8.46 (s, 1H, Hetar); 19F NMR (235 MHz, $CDCl_3$) δ = -130.96 (d, ^2J(F,F) = 285.4 Hz, 1F, C$F^a$$F^b$H), -128.50 (d, ^2J(F,F) = 285.4 Hz, 1F, C$F^a$$F^b$H); 13C NMR (63 MHz, $CDCl_3$) δ = 35.5 (s, 1C, CH_2), 70.6 (t, ^2J(C,F) = 24.7 Hz, 1C, CH(OH)CF_2H), 115.9 (t, ^1J(C,F) = 244.4 Hz, 1C, CF_2H), 122.0, 124.0, 137.2, 148.4 (1C, CH_{Hetar}), 158.2 (1C, C_{Hetar}); IR (ATR, cm^{-1}): $\tilde{v}$ = 3164, 3018, 2969, 2927, 2858, 1597, 1479, 1438, 1138, 1047, 754; MS (GC-MS): m/z (%) = 173 ($[M]^+$, 0.2), 172 (0.8), 153 (9), 133 (10), 122 (26), 94 (14), 93 (100), 92 (15), 79 (6), 78 (8), 66 (11), 65 (15), 52 (6), 51 (16), 50 (5), 39 (10); HRMS (ESI-TOF): calcd. for $C_8H_9F_2NO$ ($[M+H]^+$) 174.0725, found: $C_8H_9F_2NO$ ($[M+H]^+$) 174.07258.

4. Enantioenriched alcohols (3a-2c and 2e-2g)

(*R*)-1-(Pyridin-4-yl)propan-2-ol (3a) *from enzymatic conversion*

1-(Pyridin-4-yl)propan-2-one (**2a**) (0.3 mmol, 41 mg) is reduced to (*R*)-1-(pyridin-4-yl)propan-2-ol (**3a**) during 48 h and was purified via flash chromatography resulting in a red oil (38 mg, 93%); ee: >99%; $[\alpha]_{598}^{28}$ –15.839 (*c* 1.0, $CHCl_3$).

1H NMR (250 MHz, $CDCl_3$) δ = 1.23 (d, ^3J(H,H) = 6.1 Hz, 3H, CH_3), 2.68 - 2.75 (m, 2H, CH_2), 3.14 (s, 1H, CH(O*H*)), 3.94 - 4.16 (m, 1H, C*H*(OH)), 7.13 (d, ^3J(H,H) = 6.0 Hz, 2H, CH_{Hetar}), 8.38 (d, ^3J(H,H) = 5.7 Hz, 2H, CH_{Hetar}); 13C NMR (63 MHz, $CDCl_3$) δ = 23.2 (1C, *C*H_3), 44.9 (1C, *C*H_2), 67.7 (1C, *C*H(OH)), 124.9 (2C, CH_{Hetar}), 148.4 (1C, C_{Hetar}), 149.1 (2C, CH_{Hetar}); IR (ATR, cm^{-1}): $\tilde{\nu}$ = 3241, 3074, 2966, 2926, 2857, 1938, 1603, 1417, 1116, 1002, 938, 792, 603; MS (GC-MS): m/z (%) = 137 ($[M]^+$, 0.6), 94 (11), 93 (100), 92 (12), 67 (5), 66 (6), 65 (11), 63 (5), 51 (9), 45 (23), 43 (6), 39 (14); HRMS (EI): calcd. for $C_8H_{11}NO$ ($[M]^+$) 137.08352, found: 137.08383.

(*S*)-1-Fluoro-3-(pyridin-4-yl)propan-2-ol (3b) *from enzymatic conversion*

1-Fluoro-3-(pyridin-4-yl)propan-2-one (**2b**) (0.3 mmol, 47 mg) is reduced to (*S*)-1-fluoro-3-(pyridin-4-yl)propan-2-ol (**3b**) and was purified via flash chromatography resulting in a yellow oil (45 mg, 95%); ee: Aa, $[\alpha]_{598}^{26}$ – 23.687 (*c* 1.0, $CHCl_3$).

1H NMR (300 MHz, $CDCl_3$/Methanol-d_4) δ = 2.85 (dd, ^2J(H,H) = 13.8, ^3J(H,H) = 5.1 Hz, 1H, CH^aH^b), 2.76 (dd, ^2J(H,H) = 13.6, ^3J(H,H) = 7.9 Hz, 1H, CH^aH^b), 3.95 - 4.10 (m, 1H, C*H*(OH)), 4.31 (ddd, ^2J(H,F) = 47.2, ^2J(H,H) = 16.4, ^3J(H,H) = 9.4 Hz, 1H, CH^aH^bF), 4.32 (ddd, ^2J(H,F) = 47.4, ^2J(H,H) = 15.9, ^3J(H,H) = 9.4 Hz, 1H, CH^aH^bF), 7.25 (d, ^3J(H,H) = 5.5 Hz, 2H, Hetar), 8.39 (s, 2H, Hetar); 13C NMR (63 MHz, $CDCl_3$/Methanol-d_4) δ = 37.8 (d, ^3J(C,F) = 6.1 Hz, 1C, *C*H_2), 69.5 (d, ^2J(C,F) = 19.8 Hz, 1C, *C*H(OH)), 85.3 (d, ^1J(C,F) = 167.8 Hz, 1C, *C*FH_2), 125.0 (2C, CH_{Hetar}), 148.3 (1C, C_{Hetar}), 148.3 (2C, C_{Hetar}); IR (ATR, cm^{-1}): $\tilde{\nu}$ = 3162, 3075, 2951, 2894, 1945, 1604, 1560, 1418, 1221, 1105, 1070, 1003, 793, 603, 544; MS (GC-MS): m/z (%) = 156 ($[M+H]^+$, 5), 155 ($[M]^+$, 18), 94 (28), 93 (100), 92 (17), 78 (5), 67 (9), 66 (10), 65 (15), 64 (5), 63 (21), 52 (6), 51 (14), 50 (6), 43 (10), 39 (20), 33 (5); HRMS (EI): calcd. for $C_8H_{10}FNO$ ($[M]^+$) 155.07409, found: 155.07370.

(*S*)-1,1-Difluoro-3-(pyridin-4-yl)propan-2-ol (3c) *from enzymatic conversion*

1,1-Difluor-3-(pyridin-4-yl)propan-2-one (**2c**) (0.3 mmol, 51 mg) is reduced to (*S*)-1,1-difluor-3-(pyridin-4-yl)propan-2-ol (**3c**) and was purified via flash chromatography resulting in a yellow oil (49 mg, 95%); ee: >99%, $[\alpha]_{598}^{27}$ 1.04 (*c* 1.0, $CHCl_3$).

1H NMR (300 MHz, Acetone-d_6) δ = 2.78 (dd, ^2J(H,H) = 14.0, ^3J(H,H) = 9.8 Hz, 1H, CH^aH^b), 2.97 (dd, ^2J(H,H) = 14.0, ^3J(H,H) = 3.2 Hz, 1H, CH^aH^b), 3.55 - 4.65 (m, 1H, C*H*(OH)), 4.11 (s, 1H, O*H*), 5.86 (td,

^2J(H,F) = 55.9, ^3J(H,H) = 4.2 Hz, 1H, CF_2H) 7.32 (d, ^3J(H,H) = 5.3 Hz, 2H, Hetar), 8.45 (pd., 2H, Hetar); 19F NMR (282 MHz, Acetone-d_6) δ = -130.89 (d, ^2J(H,H) = 284.1 Hz, 1F, CF^aF^bH), -129.68 (d, ^2J(H,H) = 285.1 Hz, 1F, CF^aF^bH); 13C NMR (63 MHz, Acetone-d_6) δ = 36.8 (t, ^3J(C,F) = 3.9 Hz, 1C, CH_2), 71.8 (t, ^2J(C,F) = 24.3 Hz, 1C, *C*H(OH)), 117.9 (t, ^1J(C,F) = 242.6 Hz, 1C, CF_2H), 126.2 (2C, CH_{Hetar}), 148.4 (1C, C_{Hetar}), 150.5 (2C, C_{Hetar}); IR (ATR, cm^{-1}): $\tilde{\nu}$ = 3080, 2973, 2926, 2853, 2753, 1607, 1561, 1419, 1139, 1092, 1049, 1005, 837, 800, 593, 560, 538; MS (GC-MS): m/z (%) = 174 ($[M+H]^+$, 9), 173 ($[M]^+$, 85), 122 (42), 94 (48), 93 (100), 92 (42), 81 (5), 78 (10), 77 (5), 67 (12), 66 (14), 65 (33), 64 (5), 63 (11), 62 (6), 61 (9), 52 (10), 51 (35), 50 (13), 41 (6), 40 (5), 39 (29), 38 (6), 33 (7), 29 (5); HRMS (EI)**:** calcd. for $C_8H_9F_2NO$ ($[M]^+$) 173.06467, found: 173.06449.

(*R*)-1-(Pyridin-2-yl)propan-2-ol (3e) *from enzymatic conversion*

1-(Pyridin-2-yl)propan-2-one (**2e**) (0.3 mmol, 41 mg) is reduced to (*R*)-1-(pyridin-2-yl)propan-2-ol (**3e**) and was purified via flash chromatography resulting in an oil (13 mg, 31%); ee: >99%, $[\alpha]_{598}^{27}$ –37.73 (*c* 1.0, $CHCl_3$).

1H NMR (300 MHz, $CDCl_3$) δ = 1.29 (d, ^3J(H,H) = 6.2 Hz, 3H, CH_3), 2.93 (dd, ^2J(H,H) = 15.3, ^3J(H,H) = 3.6 Hz, 1H. CH^aH^b), 2.86 (dd, ^2J(H,H) = 14.7, ^3J(H,H) = 8.1 Hz, 1H, CH^aH^b), 4.16 - 4.31 (m, 1H, C*H*(OH)), 7.11 - 7.25 (m, 2H, Hetar), 7.65 (ddd, ^3J(H,H) = 7.6, ^3J(H,H) = 7.6, ^4J(H,H) = 1.7 Hz, 1H, Hetar), 8.51 (d, ^3J(H,H) = 4.0 Hz, 1H, Hetar); 13C NMR (63 MHz, $CDCl_3$) δ = 23.0 (1C, CH_3), 44.8 (1C, CH_2), 67.1 (1C, *C*H(OH)), 121.6, 123.7, 136.9, 148.3 (1C, CH_{Hetar}), 160.1 (1C,C_{Hetar}); IR (ATR, cm^{-1}): $\tilde{\nu}$ = 3295, 3085, 3067, 3011, 2965, 2926, 2871, 2854, 1713, 1592, 1569, 1475, 1474, 1435, 1372, 1117, 1002, 938, 755, 613; MS (GC-MS): m/z (%) = 137 ($[M]^+$, 0.55), 136 ($[M-H]^+$, 4), 122 (10), 94 (13), 93 (100), 92 (12), 80 (15), 78 (6), 66 (14), 65 (15), 51 (8), 50 (5), 45 (8), 39 (11); HRMS (EI)**:** calcd. for $C_8H_{11}NO$ ($[M+H]^+$) 138.09134, found: 138.09162.

(*S*)-1-Fluoro-3-(pyridin-2-yl)propan-2-ol (3f) *from enzymatic conversion*

1-Fluor-3-(Pyridin-2-yl)propan-2-one (**2f**) (0.3 mmol, 47 mg) is reduced to (*S*)-1-fluor-3-(pyridin-2-yl)propan-2-ol (**3f**) and was purified via flash chromatography resulting in a yellow oil (32 mg, 70%); ee: 95%, $[\alpha]_{598}^{28}$ –17.17 (*c* 1.0, $CHCl_3$).

1H NMR (300 MHz, $CDCl_3$) δ = 3.05 (dd, ^2J(H,H) = 15.3, ^3J(H,H) = 7.2 Hz, 1H, CH^aH^b), 3.10 (dd, ^2J(H,H) = 15.1, ^3J(H,H) = 4.0 Hz, 1H, CH^aH^b), 4.25 - 4.37 (m, 1H, C*H*(OH)), 4.44 (ddd, ^2J(H,H) = 46.8, ^2J(H,H) = 20.8, ^3J(H,H) = 9.1 Hz, 1H, CH^aH^bF), 4.45 (ddd, ^2J(H,H) = 46.8, ^2J(H,H) = 21.0, ^3J(H,H) = 9.3 Hz, 1H, CH^aH^bF), 7.19 - 7.25 (m, 2H, Hetar), 7.68 (ddd, ^3J(H,H) = 7.7, ^3J(H,H) = 7.7, ^4J(H,H) = 1.6 Hz, 1H, Hetar), 8.51 (dd, ^3J(H,H) = 5.4, ^4J(H,H) = 1.8 Hz, 1H, Hetar); 13C NMR (63 MHz, $CDCl_3$) δ = 38.5 (d, ^3J(C,F) = 5.5 Hz, 1C, CH_2), 69.5 (d, ^2J(C,F) = 20.6 Hz, 1C, *C*H(OH)), 85.7 (d, ^1J(C,F) = 170.7 Hz, 1C, CFH_2), 121.9, 124.0, 137.2, 148.3 (1C, CH_{Hetar}), 158.9 (1C, C_{Hetar}); IR (ATR, cm^{-1}): $\tilde{\nu}$ = 3258, 3083, 2953, 2923, 2852, 1632, 1595, 1504, 1436, 1106, 1004, 762, 624, 554; MS (GC-MS): m/z (%) = 138 (2), 136

(2), 122 (15), 94 (14), 93 (100), 92 (14), 78 (8), 67 (6), 66 (10), 65 (9), 63 (7), 52 (5), 51 (7), 39 (9); HRMS (EI): calcd. for $C_8H_{10}FNO$ ($[M+H]^+$) 156.08192, found: 156.08187.

(*S*)-1,1-Difluor-3-(pyridin-2-yl)propan-2-ol (3g) *from enzymatic conversion*

1,1-Difluor-3-(pyridin-2-yl)propan-2-one (**2g**) (0.3 mmol, 51 mg) is reduced to (*S*)-1,1-difluor-3-(pyridin-2-yl)propan-2-ol (**3g**) and was purified via flash chromatography resulting in a yellow oil (30 mg, 60%); $[\alpha]_{598}^{22}$ –13.21 (*c* 1.0, CH_2Cl_2).

1H NMR (300 MHz $CDCl_3$) δ = 3.05 - 3.18 (m, 2H, C*H*$_2$), 4.15 - 4.36 (m, 1H, C*H*(OH)), 4.87 (s, 1H, CH(O*H*)), 5.8 (td, ^2J(H,F) = 56.10, ^3J(H,H) = 3.97 Hz, 1H, C*H*F_2), 7.25 (d, ^3J(H,H) = 6.99 Hz, 2H, Hetar), 7.69 (ddd, ^3J(H,H) = 7.70, ^3J(H,H) = 7.70, ^4J(H,H) = 1.79 Hz, 1H, Hetar), 8.50 (s, 1H, Hetar); 19F NMR (282 MHz, $CDCl_3$) δ = -131.0 (d, $^2J(F,F)$ = 287.10 Hz, 1F, CF^aF^bH), -128.4 (d, $^2J(F,F)$ = 286.10 Hz, 1F, CF^aF^bH); 13C NMR (63 MHz, extern/DMSO-d_6) δ = 39.9 (s, 1C, *C*H_2), 75.3 (t, ^2J(C,F) = 25.2 Hz, 1C, *C*H(OH)), 120.6 (t, ^1J(C,F) = 248.1 Hz, 1C, *C*HF_2), 126.7, 128.7, 142.0, 153.0 (1C, CH_{Hetar}), 163.0 (1C, C_{Hetar}); IR (ATR, cm^{-1}): $\tilde{\nu}$ = 3159 (w), 3018, 2956, 2921, 2852, 1597, 1439, 1138, 1047, 753, 541; MS (GC-MS): m/z (%) = 172 ($[M-1]^+$, 1), 153 (10), 133 (8), 122 (26), 94 (15), 93 (100), 92 (16), 79 (6), 78 (9), 66 (10), 65 (18), 52 (7), 51 (16), 50 (6), 39 (11); HRMS (EI): calcd. for $C_8H_8F_2NO$ ($[M-H]^+$) 172.05685, found: 172.05691.

5. NMR-spectra for ketones (2)

Acquisition Time (sec)	5.2953
Date	05 Nov 2014 12:46:08

Frequency (MHz)	300.13	*Nucleus*	1H	*Number of Transients*	16	*Origin*	spect
Original Points Count	32768	*Owner*	nmrsu	*Points Count*	32768	*Pulse Sequence*	zg30
Receiver Gain	128.00	*SW(cyclical) (Hz)*	6188.12	*Solvent*	CHLOROFORM-d		
Spectrum Offset (Hz)	1850.4254	*Spectrum Type*	STANDARD	*Sweep Width (Hz)*	6187.93	*Temperature (degree C)*	25.160

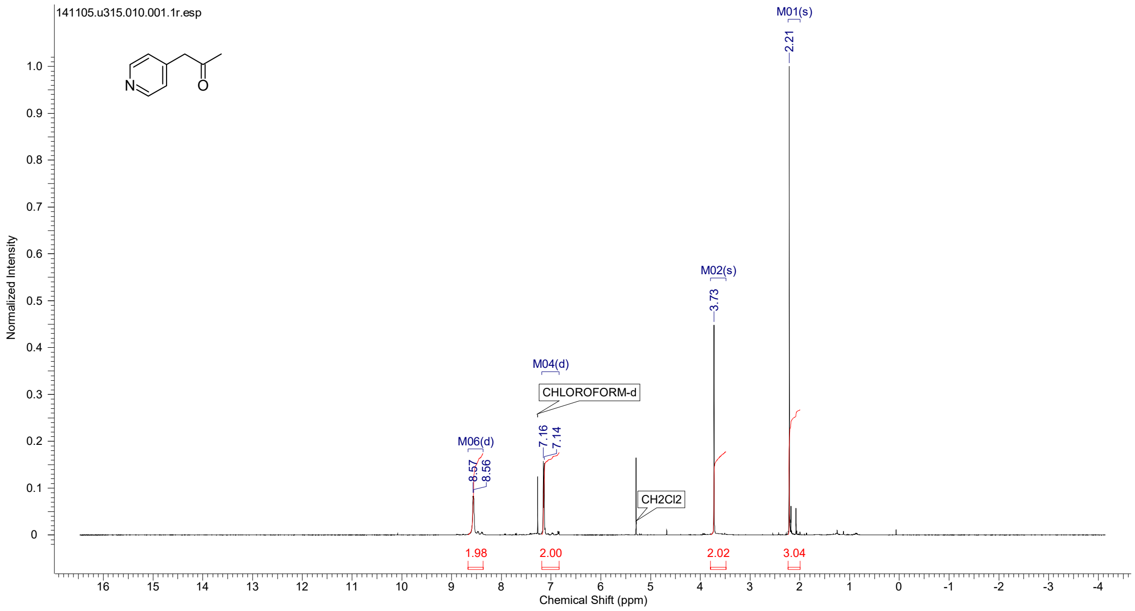

Acquisition Time (sec)	1.8175	*Comment*				*Date*	06 Nov 2014 01:25:36
Date Stamp	06 Nov 2014 01:25:36						
File Name							
Frequency (MHz)	75.47	*Nucleus*	13C	*Number of Transients*	1024	*Origin*	spect
Original Points Count	32768	*Owner*	nmrsu	*Points Count*	32768	*Pulse Sequence*	zgpg30
Receiver Gain	2050.00	*SW(cyclical) (Hz)*	18028.85	*Solvent*	CHLOROFORM-d	*Spectrum Offset (Hz)*	7543.3115
Spectrum Type	STANDARD	*Sweep Width (Hz)*	18028.29	*Temperature (degree C)*	25.160		

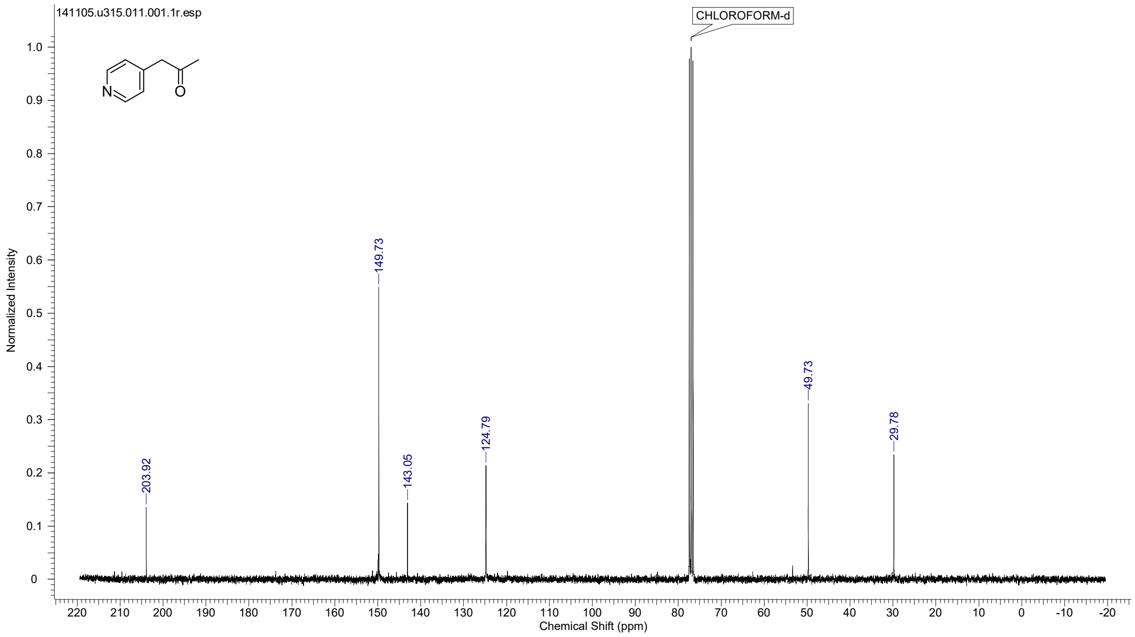

Acquisition Time (sec)	5.2953						
Date Stamp	28 Jul 2015 08:13:20						
Frequency (MHz)	300.13	*Nucleus*	1H	*Number of Transients*	16	*Origin*	spect
Original Points Count	32768	*Owner*	nmrsu	*Points Count*	32768	*Pulse Sequence*	zg30
Receiver Gain	228.00	*SW(cyclical) (Hz)*	6188.12	*Solvent*	CHLOROFORM-d		
Spectrum Offset (Hz)	1850.4255	*Spectrum Type*	STANDARD	*Sweep Width (Hz)*	6187.93	*Temperature (degree C)*	25.160

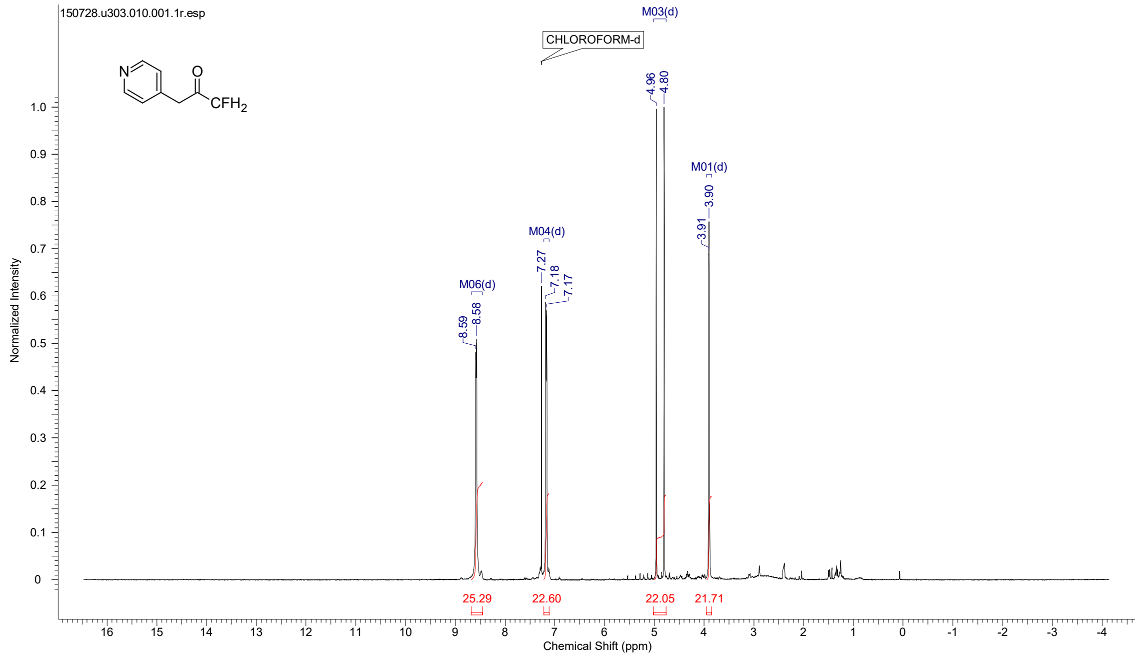

Acquisition Time (sec)	2.1845						
Date Stamp	29 Jul 2015 11:06:08						
Frequency (MHz)	62.90	*Nucleus*	13C	*Number of Transients*	1024	*Origin*	spect
Original Points Count	32768	*Owner*	nmr	*Points Count*	32768	*Pulse Sequence*	zgpg30
Receiver Gain	2050.00	*SW(cyclical) (Hz)*	15000.00	*Solvent*	CHLOROFORM-d		
Spectrum Offset (Hz)	6287.8647	*Spectrum Type*	STANDARD	*Sweep Width (Hz)*	14999.54	*Temperature (degree C)*	25.060

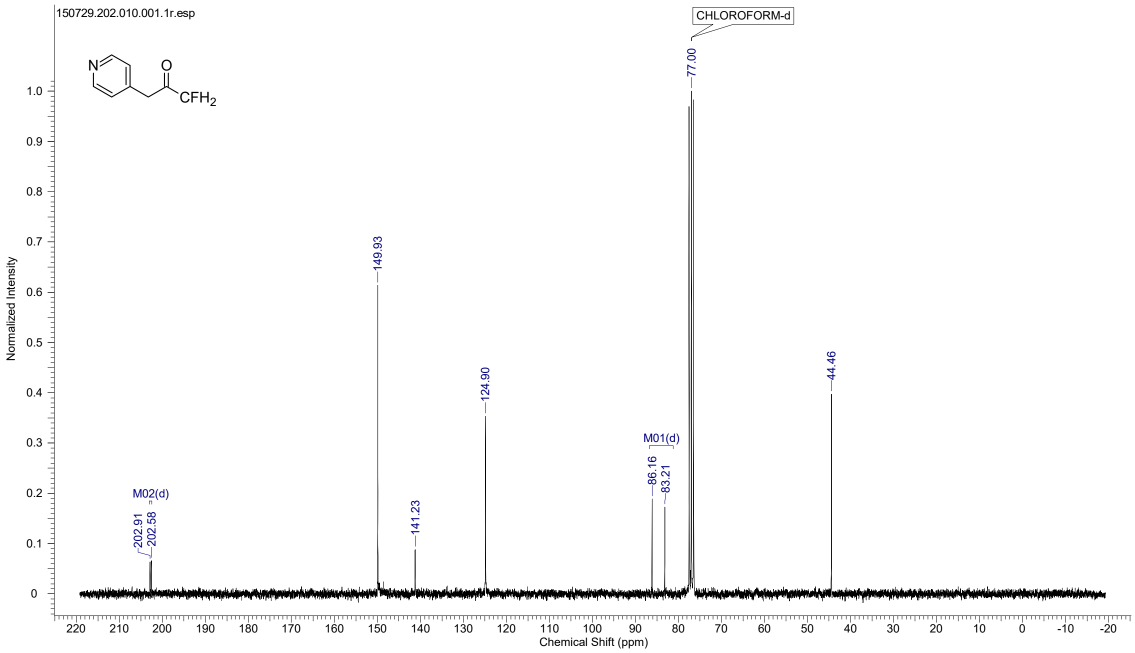

Acquisition Time (sec)	3.1719
Date Stamp	06 Aug 2015 12:18:40

						Frequency (MHz)	500.13
Nucleus	1H	Number of Transients	16	Origin	spect	Original Points Count	32768
Owner	Administrator	Points Count	32768	Pulse Sequence	zg30	Receiver Gain	90.50
SW(cyclical) (Hz)	10330.58	Solvent	METHANOL-d4	Spectrum Offset (Hz)	3077.7449	Spectrum Type	STANDARD
Sweep Width (Hz)	10330.26	Temperature (degree C)	26.951				

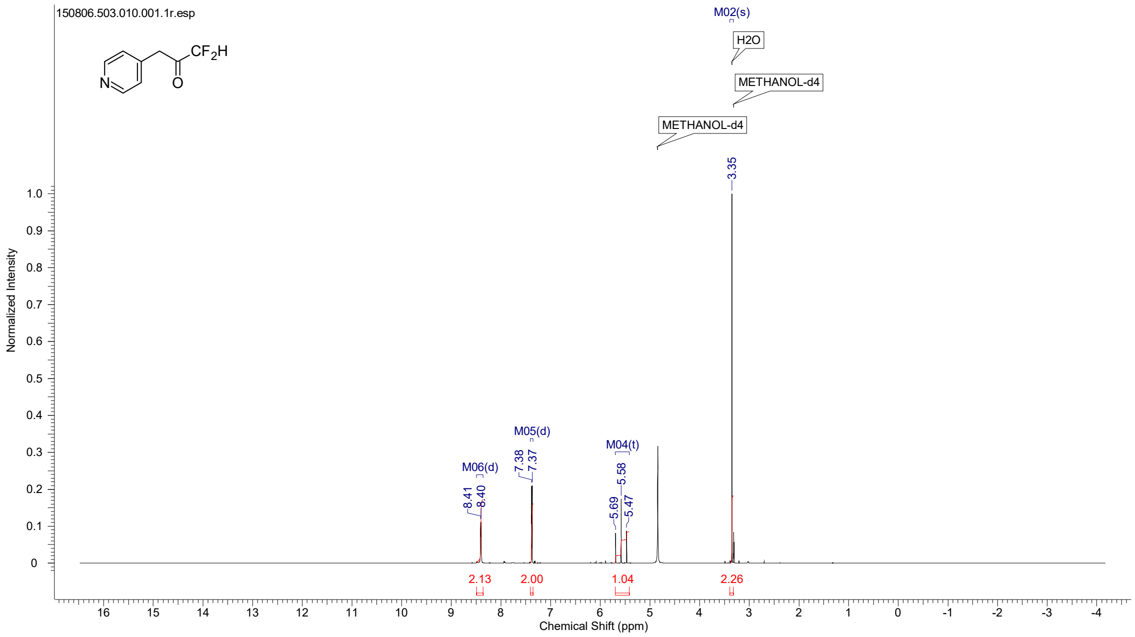

Acquisition Time (sec)	1.0912
Date Stamp	06 Aug 2015 13:16:16

						Frequency (MHz)	125.76
Nucleus	13C	Number of Transients	1024	Origin	spect	Original Points Count	32768
Owner	Administrator	Points Count	32768	Pulse Sequence	zgpg30	Receiver Gain	20642.50
SW(cyclical) (Hz)	30030.03	Solvent	METHANOL-d4	Spectrum Offset (Hz)	12768.8428	Spectrum Type	STANDARD
Sweep Width (Hz)	30029.11	Temperature (degree C)	26.951				

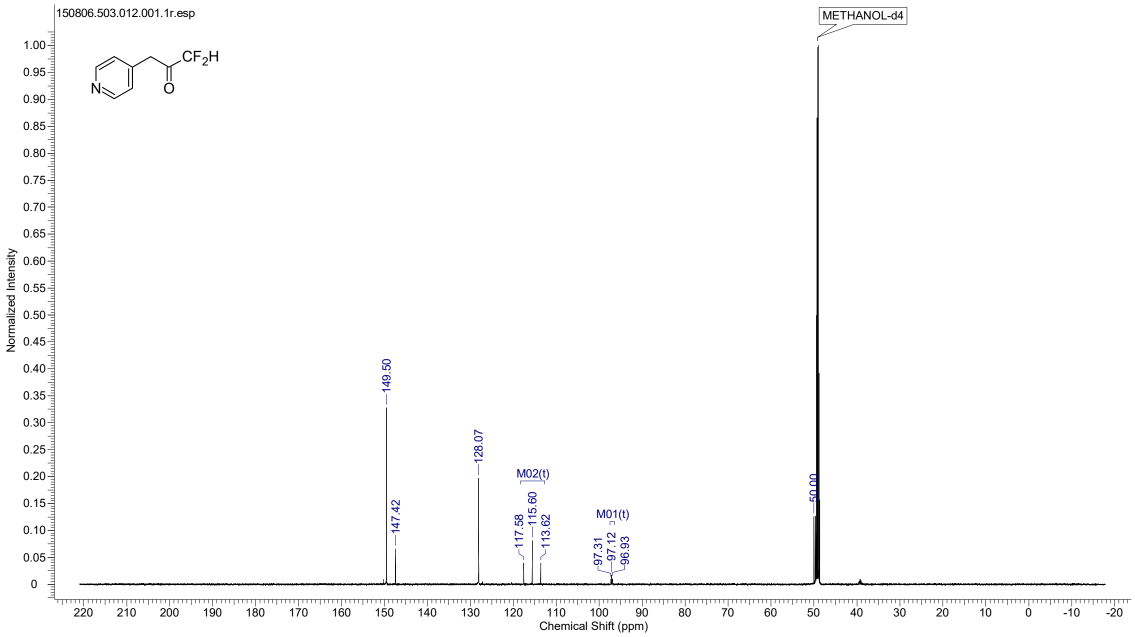

Acquisition Time (sec)	5.2953						
Date Stamp	24 Jul 2015 08:45:20						
Frequency (MHz)	300.13	Nucleus	1H	Number of Transients	16	Origin	spect
Original Points Count	32768	Owner	nmrsu	Points Count	32768	Pulse Sequence	zg30
Receiver Gain	161.00	SW(cyclical) (Hz)	6188.12	Solvent	DMSO-d6	Spectrum Offset (Hz)	1852.1492
Spectrum Type	STANDARD	Sweep Width (Hz)	6187.93	Temperature (degree C)	25.160		

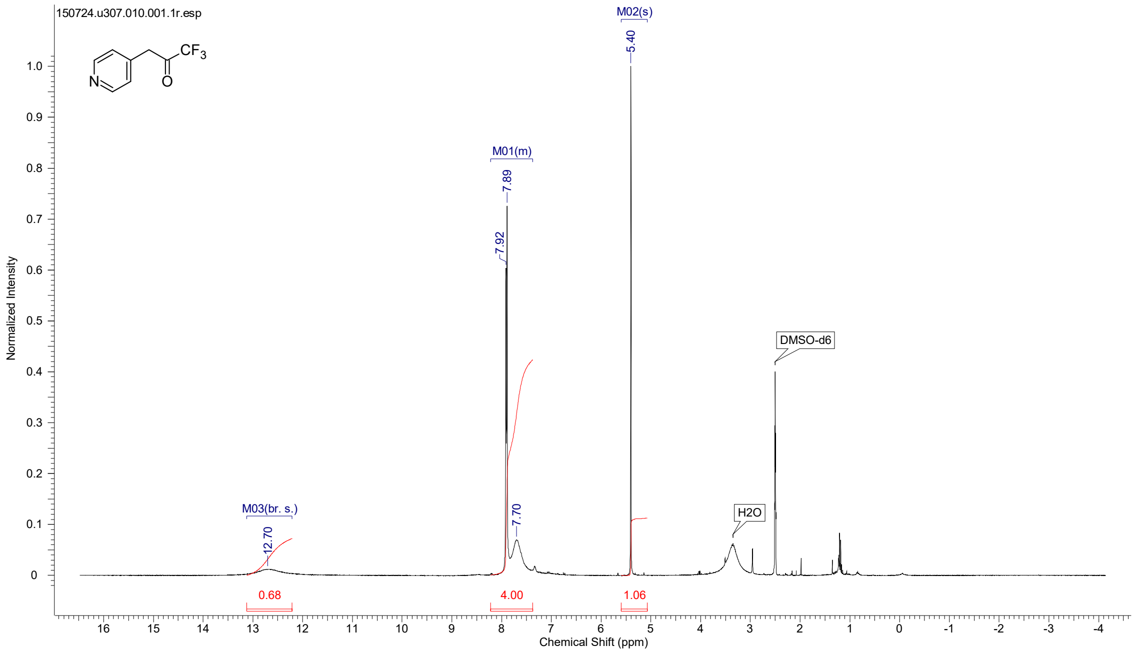

Acquisition Time (sec)	1.8175						
Date Stamp	24 Jul 2015 12:40:00						
Frequency (MHz)	75.47	Nucleus	13C	Number of Transients	1024	Origin	spect
Original Points Count	32768	Owner	nmrsu	Points Count	32768	Pulse Sequence	zgpg30
Receiver Gain	2050.00	SW(cyclical) (Hz)	18028.85	Solvent	DMSO-d6	Spectrum Offset (Hz)	7511.7764
Spectrum Type	STANDARD	Sweep Width (Hz)	18028.29	Temperature (degree C)	26.360		

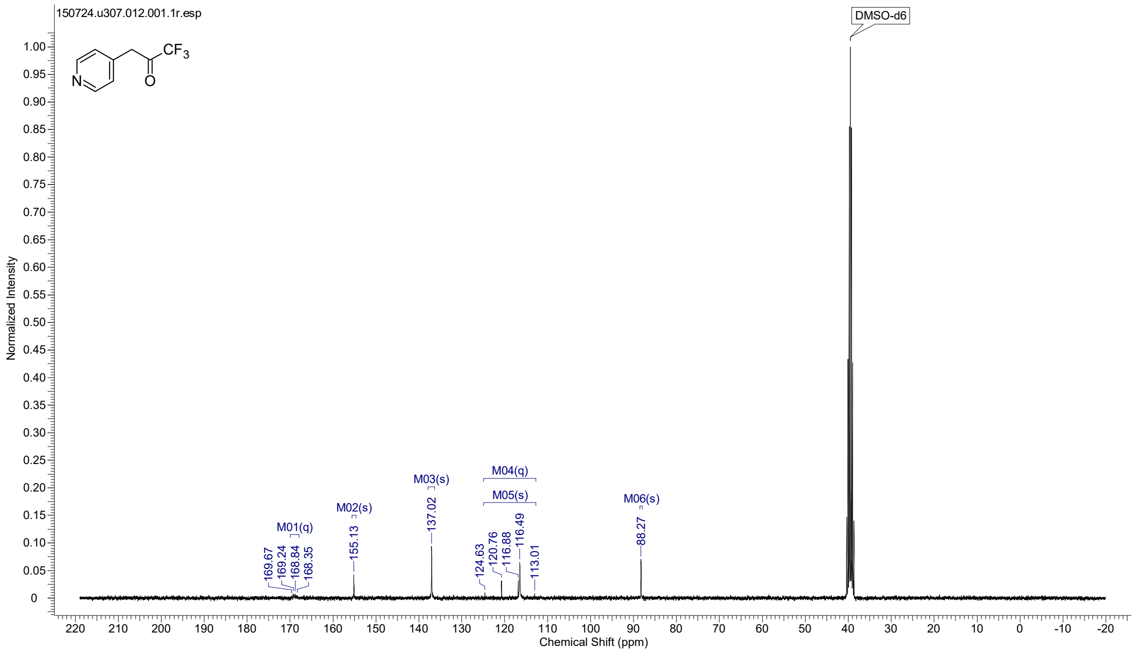

Acquisition Time (sec)	5.2953						
Date Stamp	21 May 2015 10:40:32						
Frequency (MHz)	300.13	*Nucleus*	1H	*Number of Transients*	16	*Origin*	spect
Original Points Count	32768	*Owner*	nmrsu	*Points Count*	32768	*Pulse Sequence*	zg30
Receiver Gain	50.80	*SW(cyclical) (Hz)*	6188.12	*Solvent*	CHLOROFORM-d		
Spectrum Offset (Hz)	1850.4255	*Spectrum Type*	STANDARD	*Sweep Width (Hz)*	6187.93	*Temperature (degree C)*	25.160

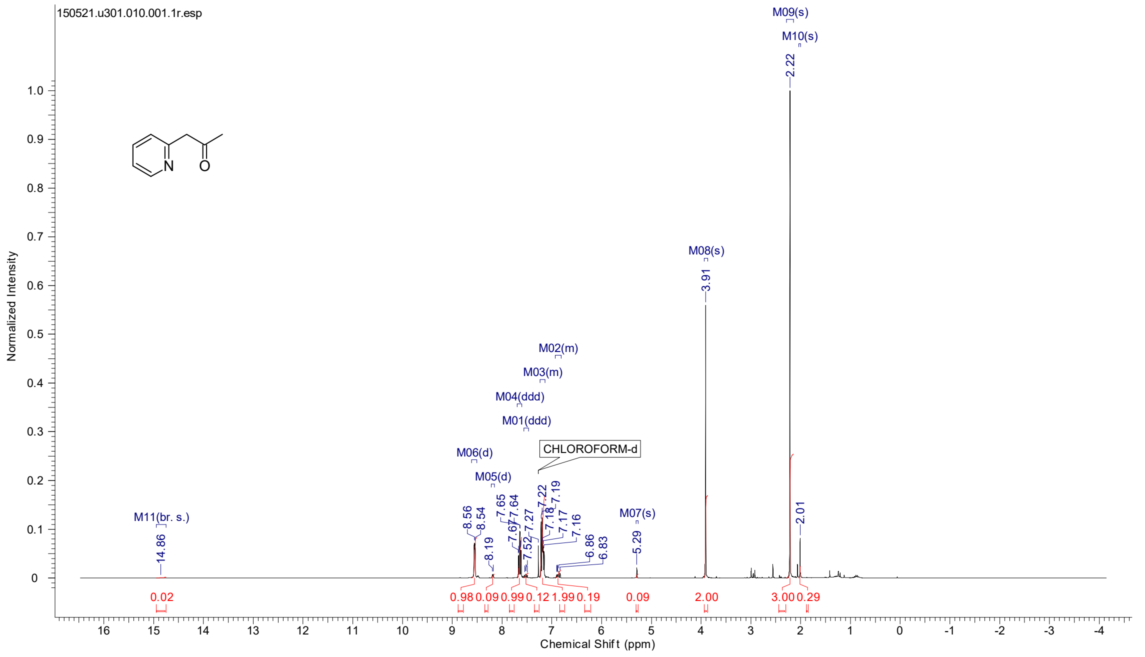

Acquisition Time (sec)	1.8175						
Date Stamp	10 Apr 2015 19:31:44						
Frequency (MHz)	75.47	*Nucleus*	13C	*Number of Transients*	1024	*Origin*	spect
Original Points Count	32768	*Owner*	nmrsu	*Points Count*	32768	*Pulse Sequence*	zgpg30
Receiver Gain	2050.00	*SW(cyclical) (Hz)*	18028.85	*Solvent*	CHLOROFORM-d		
Spectrum Offset (Hz)	7542.2104	*Spectrum Type*	STANDARD	*Sweep Width (Hz)*	18028.29	*Temperature (degree C)*	25.160

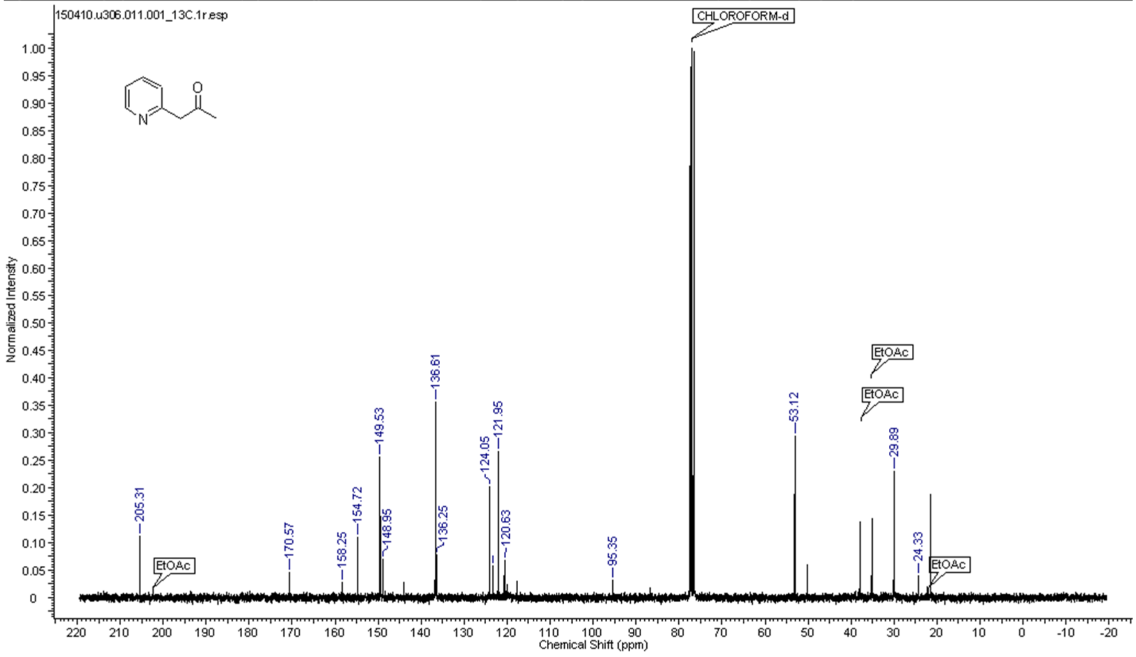

Acquisition Time (sec)	5.2953						
Date Stamp	21 Jul 2015 08:13:20						
Frequency (MHz)	300.13	*Nucleus*	1H	*Number of Transients*	16	*Origin*	spect
Original Points Count	32768	*Owner*	nmrsu	*Points Count*	32768	*Pulse Sequence*	zg30
Receiver Gain	50.80	*SW(cyclical) (Hz)*	6188.12	*Solvent*	CHLOROFORM-d		
Spectrum Offset (Hz)	1850.4255	*Spectrum Type*	STANDARD	*Sweep Width (Hz)*	6187.93	*Temperature (degree C)*	25.160

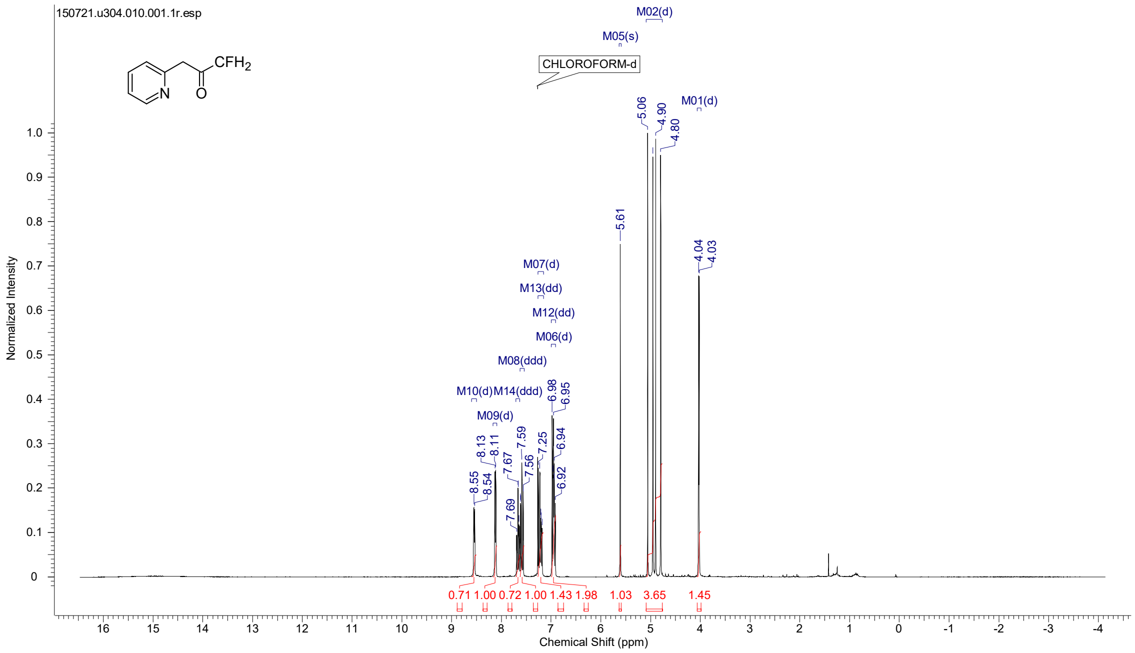

Acquisition Time (sec)	1.8175						
Date Stamp	21 Jul 2015 18:27:44						
Frequency (MHz)	75.47	*Nucleus*	13C	*Number of Transients*	2048	*Origin*	spect
Original Points Count	32768	*Owner*	nmrsu	*Points Count*	32768	*Pulse Sequence*	zgpg30
Receiver Gain	2050.00	*SW(cyclical) (Hz)*	18028.85	*Solvent*	CHLOROFORM-d		
Spectrum Offset (Hz)	7541.6602	*Spectrum Type*	STANDARD	*Sweep Width (Hz)*	18028.29	*Temperature (degree C)*	27.260

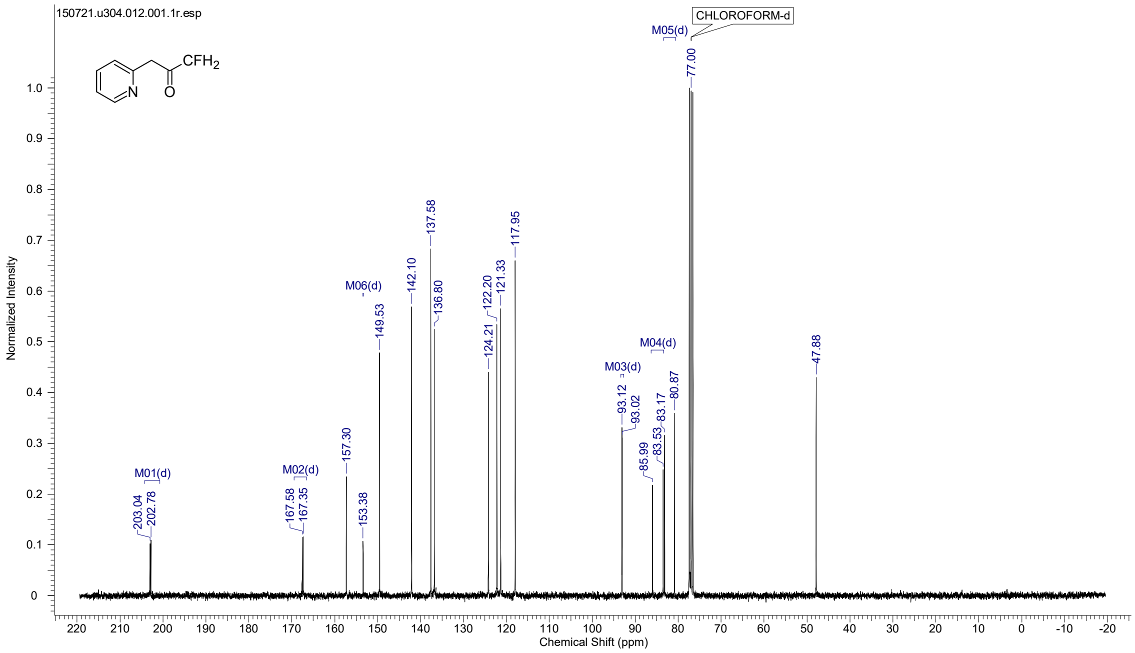

Acquisition Time (sec)	5.2953						
Date Stamp	16 Jun 2015 13:09:52						
Frequency (MHz)	300.13	*Nucleus*	1H	*Number of Transients*	16	*Origin*	spect
Original Points Count	32768	*Owner*	nmrsu	*Points Count*	32768	*Pulse Sequence*	zg30
Receiver Gain	181.00	*SW(cyclical) (Hz)*	6188.12	*Solvent*	CHLOROFORM-d		
Spectrum Offset (Hz)	1850.4255	*Spectrum Type*	STANDARD	*Sweep Width (Hz)*	6187.93	*Temperature (degree C)*	25.160

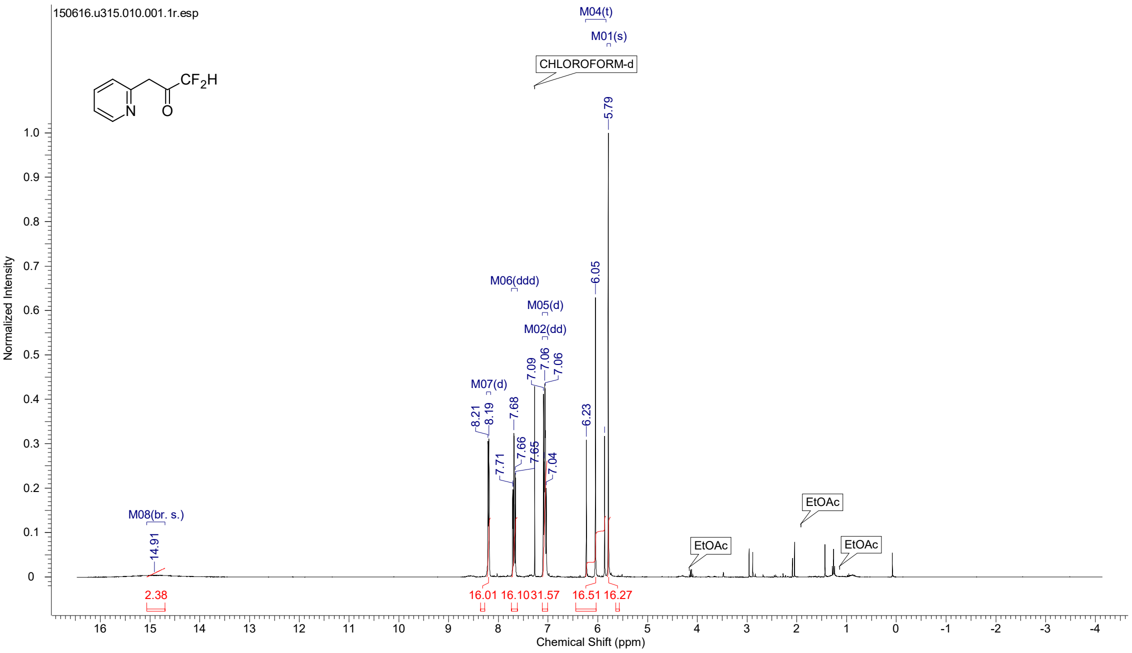

Acquisition Time (sec)	1.8175						
Date Stamp	16 Jun 2015 20:29:20						
Frequency (MHz)	75.47	*Nucleus*	13C	*Number of Transients*	1024	*Origin*	spect
Original Points Count	32768	*Owner*	nmrsu	*Points Count*	32768	*Pulse Sequence*	zgpg30
Receiver Gain	2050.00	*SW(cyclical) (Hz)*	18028.85	*Solvent*	CHLOROFORM-d		
Spectrum Offset (Hz)	7544.4111	*Spectrum Type*	STANDARD	*Sweep Width (Hz)*	18028.29	*Temperature (degree C)*	25.860

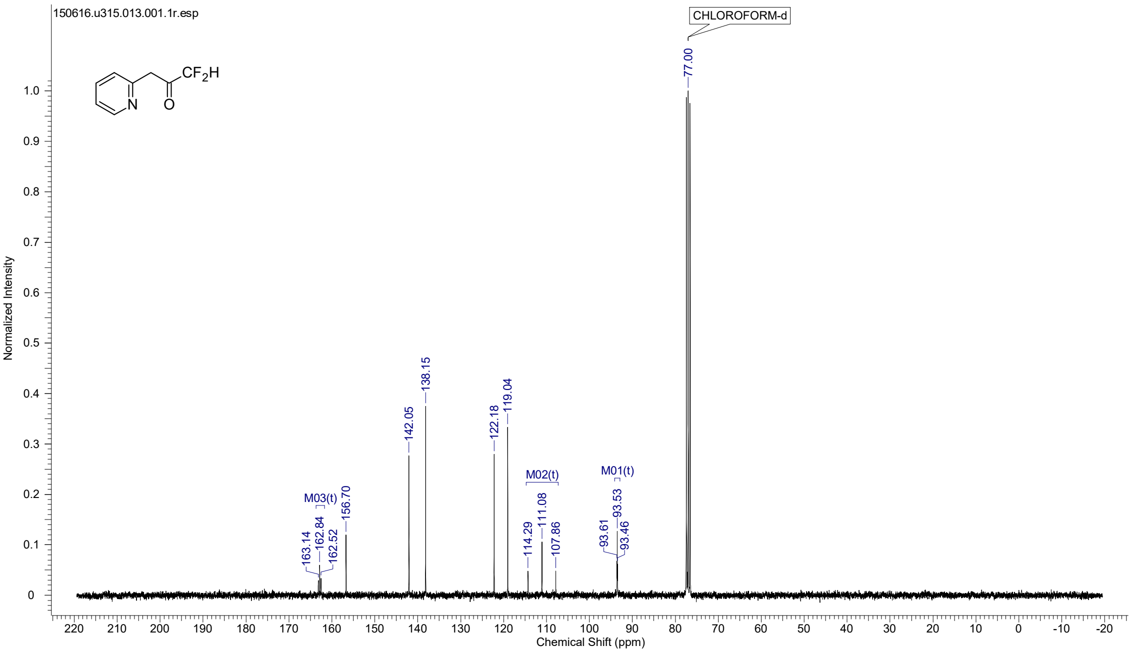

Acquisition Time (sec)	5.2953						
Date Stamp	01 Jun 2015 08:41:04						
Frequency (MHz)	300.13	Nucleus	1H	Number of Transients	16	Origin	spect
Original Points Count	32768	Owner	nmrsu	Points Count	32768	Pulse Sequence	zg30
Receiver Gain	181.00	SW(cyclical) (Hz)	6188.12	Solvent	DMSO-d6	Spectrum Offset (Hz)	1852.3380
Spectrum Type	STANDARD	Sweep Width (Hz)	6187.93	Temperature (degree C)	25.160		

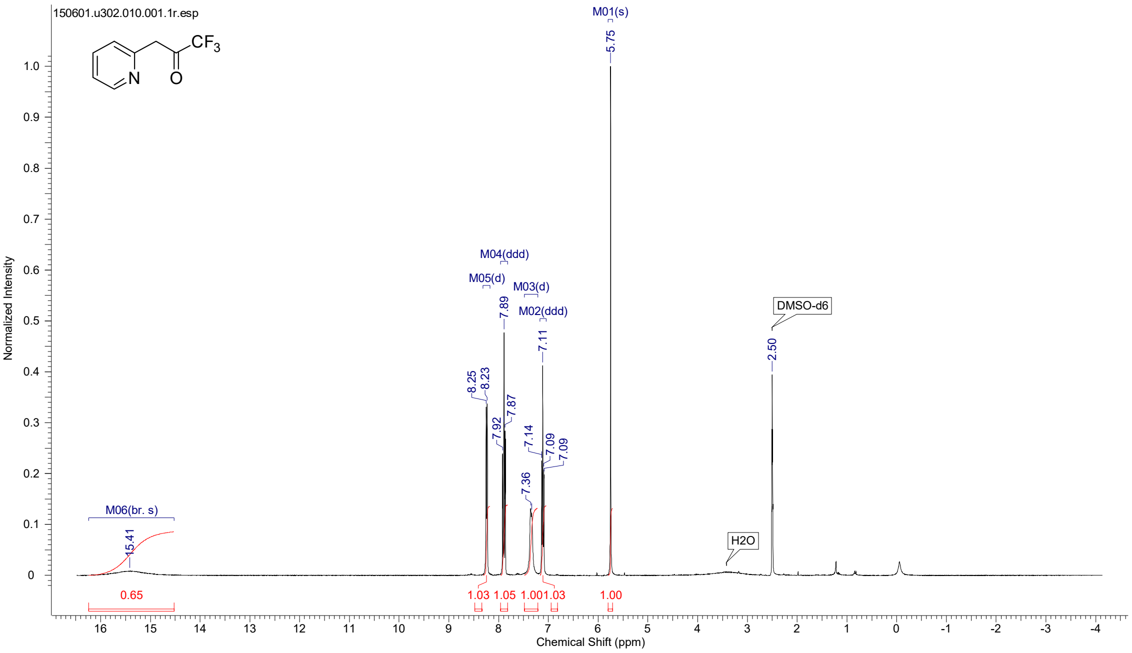

Acquisition Time (sec)	2.1845						
Date Stamp	02 Jun 2015 17:30:08						
Frequency (MHz)	62.90	Nucleus	13C	Number of Transients	1024	Origin	spect
Original Points Count	32768	Owner	nmr	Points Count	32768	Pulse Sequence	zgpg30
Receiver Gain	2050.00	SW(cyclical) (Hz)	15000.00	Solvent	DMSO-d6	Spectrum Offset (Hz)	6261.7710
Spectrum Type	STANDARD	Sweep Width (Hz)	14999.54	Temperature (degree C)	25.060		

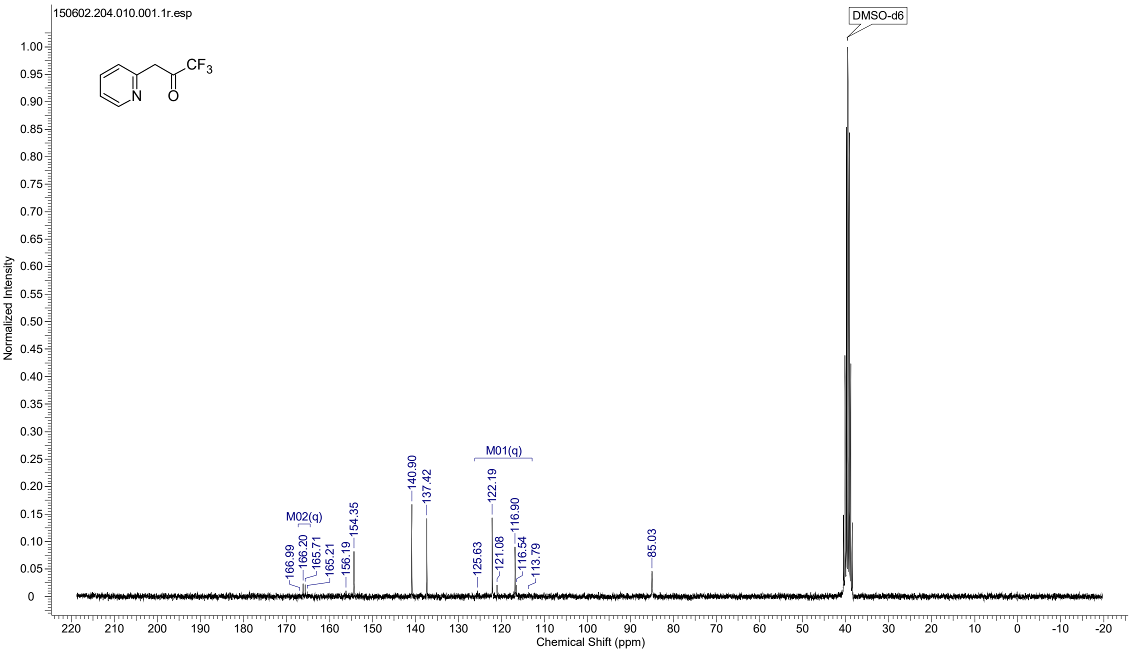

Acquisition Time (sec)	5.2953						
Date Stamp	06 Jul 2015 13:24:48						
Frequency (MHz)	300.13	***Nucleus***	1H	***Number of Transients***	16	***Origin***	spect
Original Points Count	32768	***Owner***	nmrsu	***Points Count***	32768	***Pulse Sequence***	zg30
Receiver Gain	256.00	***SW(cyclical) (Hz)***	6188.12	***Solvent***	CHLOROFORM-d		
Spectrum Offset (Hz)	1850.4255	***Spectrum Type***	STANDARD	***Sweep Width (Hz)***	6187.93	***Temperature (degree C)***	26.760

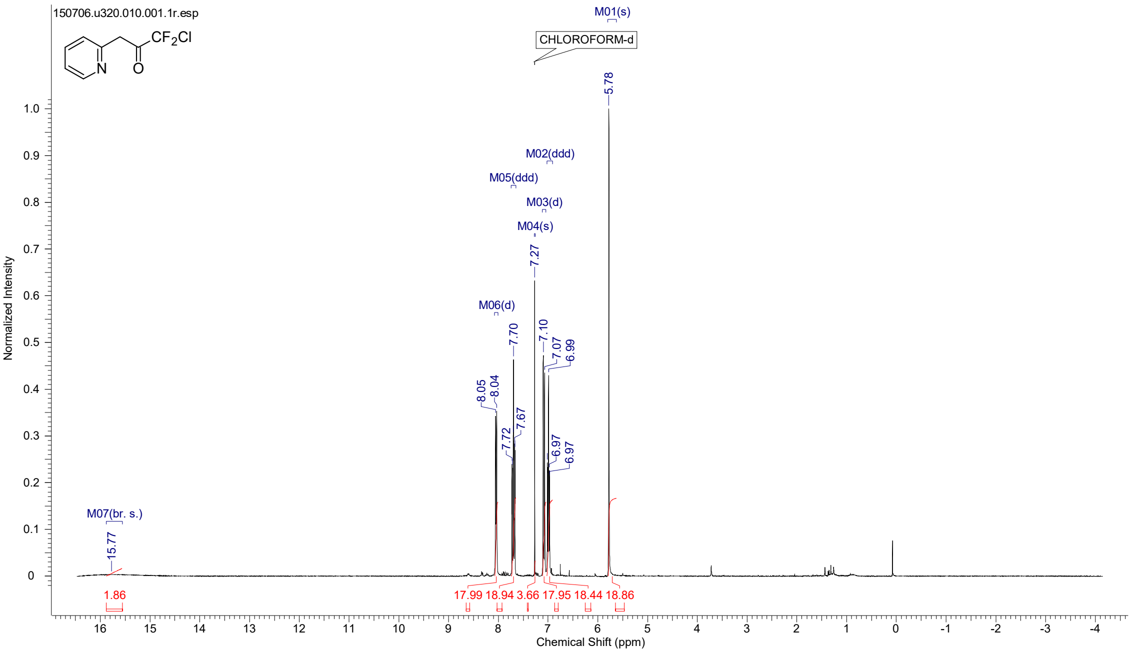

Acquisition Time (sec)	1.8175						
Date Stamp	07 Jul 2015 08:00:32						
Frequency (MHz)	75.47	***Nucleus***	13C	***Number of Transients***	1024	***Origin***	spect
Original Points Count	32768	***Owner***	nmrsu	***Points Count***	32768	***Pulse Sequence***	zgpg30
Receiver Gain	2050.00	***SW(cyclical) (Hz)***	18028.85	***Solvent***	CHLOROFORM-d		
Spectrum Offset (Hz)	7544.4111	***Spectrum Type***	STANDARD	***Sweep Width (Hz)***	18028.29	***Temperature (degree C)***	26.560

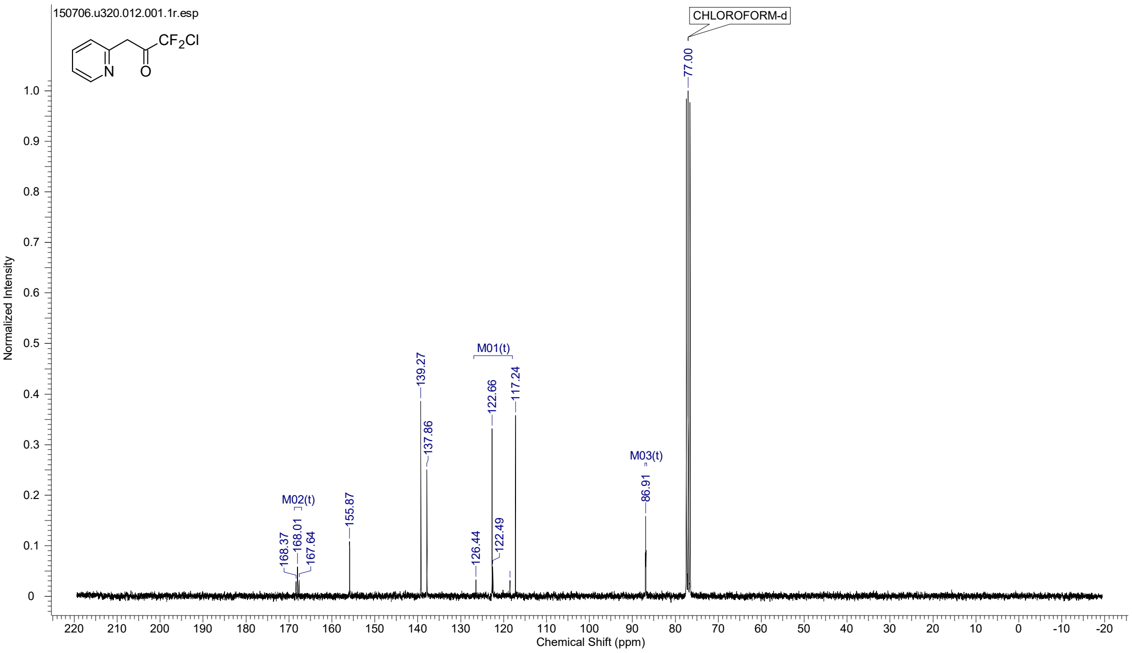

Acquisition Time (sec)	5.2953						
Date Stamp	10 Apr 2015 09:23:44						
Frequency (MHz)	300.13	Nucleus	1H	Number of Transients	16	Origin	spect
Original Points Count	32768	Owner	nmrsu	Points Count	32768	Pulse Sequence	zg30
Receiver Gain	50.80	SW(cyclical) (Hz)	6188.12	Solvent	CHLOROFORM-d		
Spectrum Offset (Hz)	1850.6144	Spectrum Type	STANDARD	Sweep Width (Hz)	6187.93	Temperature (degree C)	25.160

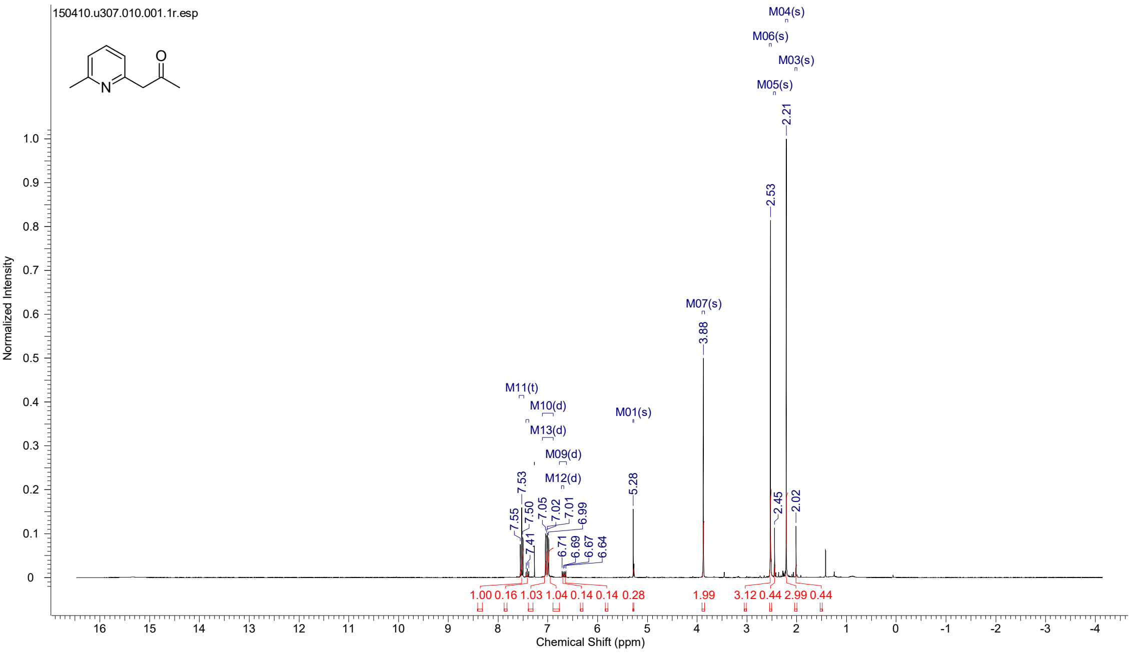

Acquisition Time (sec)	1.8175						
Date Stamp	10 Apr 2015 21:01:20						
Frequency (MHz)	75.47	Nucleus	13C	Number of Transients	1024	Origin	spect
Original Points Count	32768	Owner	nmrsu	Points Count	32768	Pulse Sequence	zgpg30
Receiver Gain	2050.00	SW(cyclical) (Hz)	18028.85	Solvent	CHLOROFORM-d		
Spectrum Offset (Hz)	7542.2104	Spectrum Type	STANDARD	Sweep Width (Hz)	18028.29	Temperature (degree C)	25.160

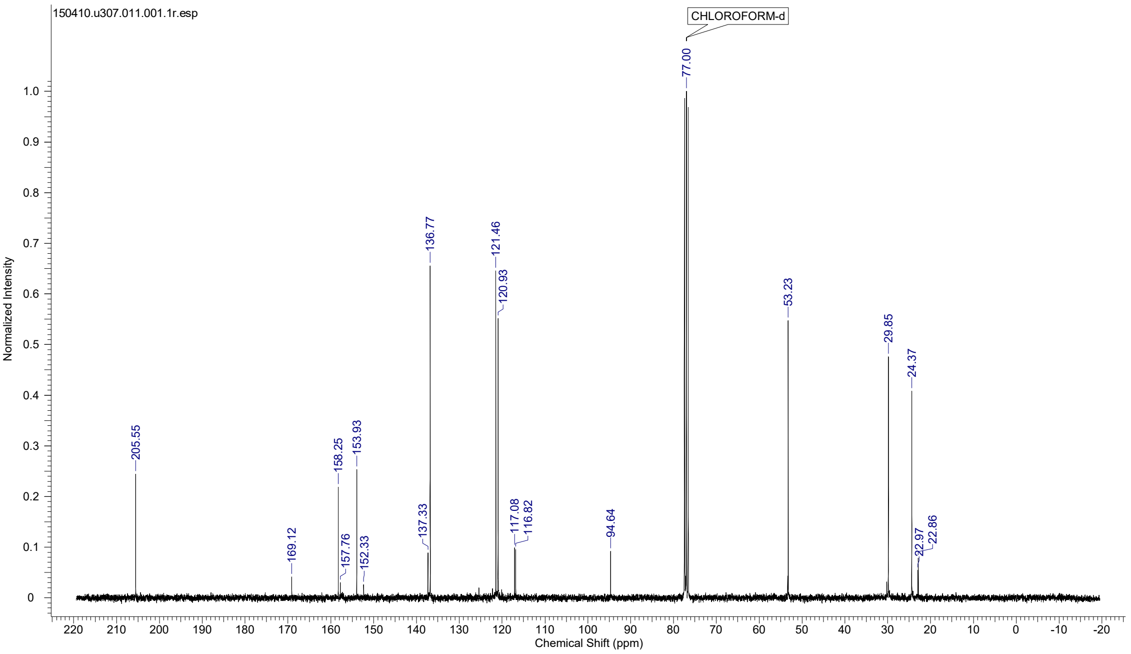

Acquisition Time (sec)	5.2953						
Date Stamp	06 Jul 2015 13:14:08						
Frequency (MHz)	300.13	*Nucleus*	1H	*Number of Transients*	16	*Origin*	spect
Original Points Count	32768	*Owner*	nmrsu	*Points Count*	32768	*Pulse Sequence*	zg30
Receiver Gain	256.00	*SW(cyclical) (Hz)*	6188.12	*Solvent*	CHLOROFORM-d		
Spectrum Offset (Hz)	1850.2366	*Spectrum Type*	STANDARD	*Sweep Width (Hz)*	6187.93	*Temperature (degree C)*	26.760

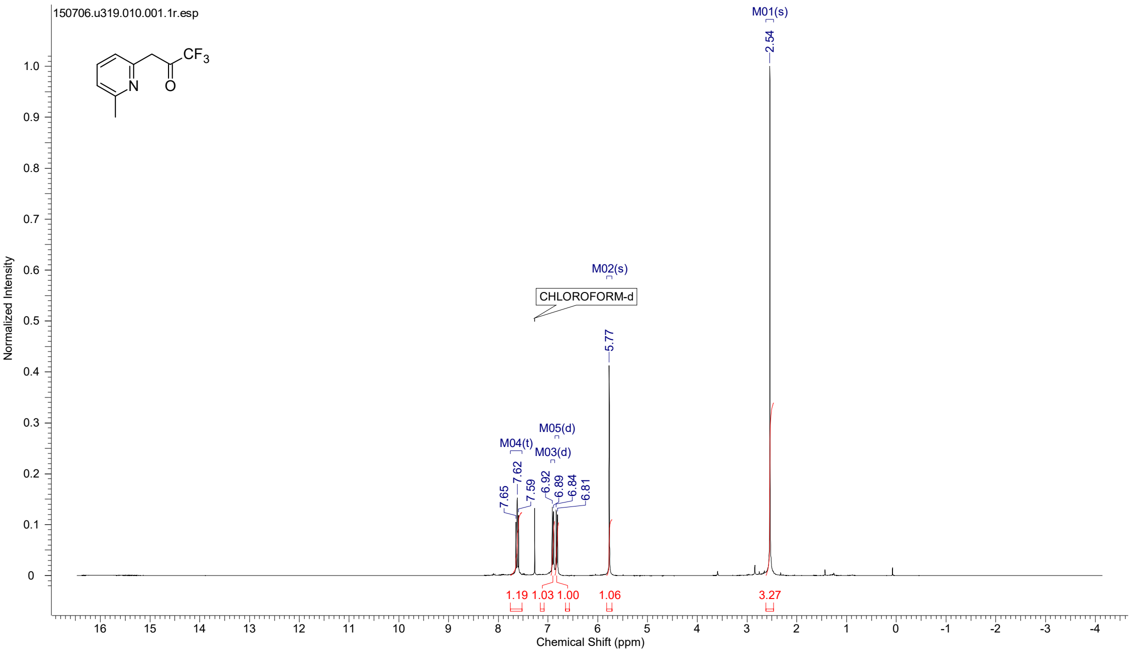

Acquisition Time (sec)	1.8175						
Date Stamp	07 Jul 2015 06:33:04						
Frequency (MHz)	75.47	*Nucleus*	13C	*Number of Transients*	1024	*Origin*	spect
Original Points Count	32768	*Owner*	nmrsu	*Points Count*	32768	*Pulse Sequence*	zgpg30
Receiver Gain	2050.00	*SW(cyclical) (Hz)*	18028.85	*Solvent*	CHLOROFORM-d		
Spectrum Offset (Hz)	7544.9619	*Spectrum Type*	STANDARD	*Sweep Width (Hz)*	18028.29	*Temperature (degree C)*	26.660

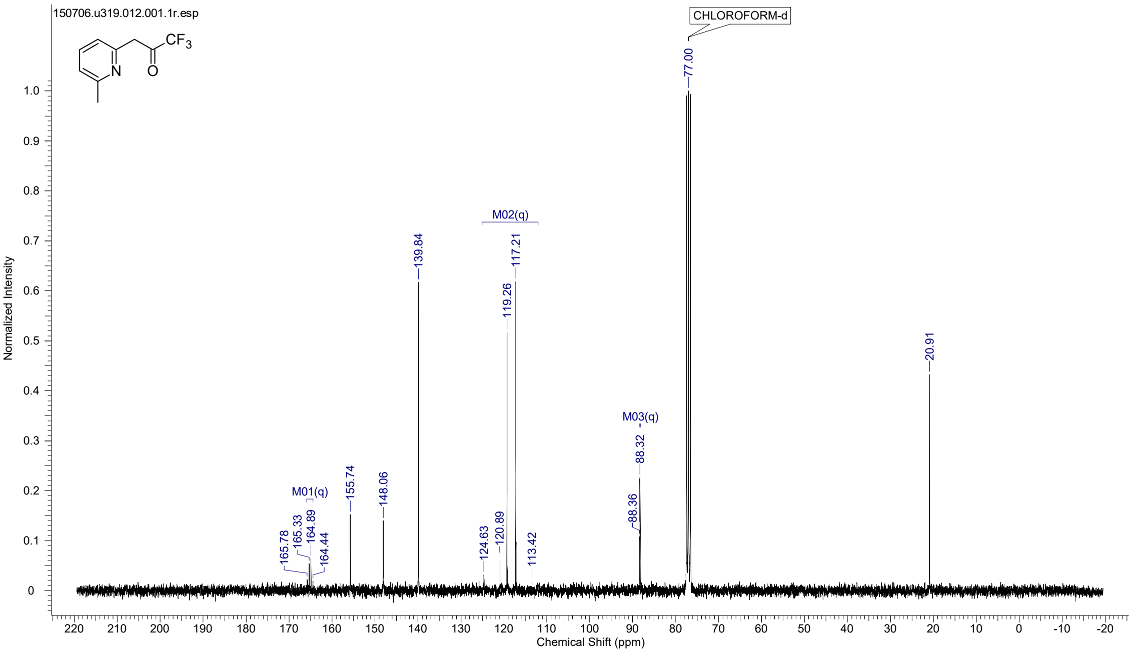

Acquisition Time (sec)	5.2953						
Date Stamp	30 Apr 2015 17:06:40						
Frequency (MHz)	300.13	*Nucleus*	1H	*Number of Transients*	16	*Origin*	spect
Original Points Count	32768	*Owner*	nmrsu	*Points Count*	32768	*Pulse Sequence*	zg30
Receiver Gain	50.80	*SW(cyclical) (Hz)*	6188.12	*Solvent*	CHLOROFORM-d		
Spectrum Offset (Hz)	1850.4254	*Spectrum Type*	STANDARD	*Sweep Width (Hz)*	6187.93	*Temperature (degree C)*	25.160

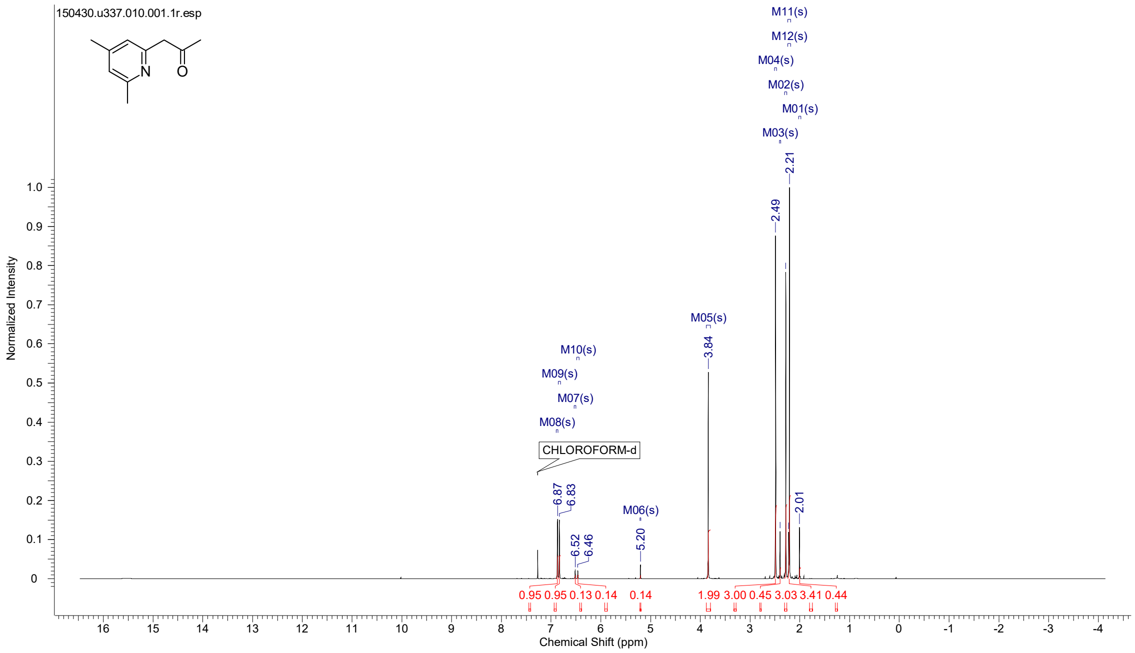

Acquisition Time (sec)	1.8175						
Date Stamp	01 May 2015 10:55:28						
Frequency (MHz)	75.47	*Nucleus*	13C	*Number of Transients*	1024	*Origin*	spect
Original Points Count	32768	*Owner*	nmrsu	*Points Count*	32768	*Pulse Sequence*	zgpg30
Receiver Gain	2050.00	*SW(cyclical) (Hz)*	18028.85	*Solvent*	CHLOROFORM-d		
Spectrum Offset (Hz)	7542.7612	*Spectrum Type*	STANDARD	*Sweep Width (Hz)*	18028.29	*Temperature (degree C)*	25.160

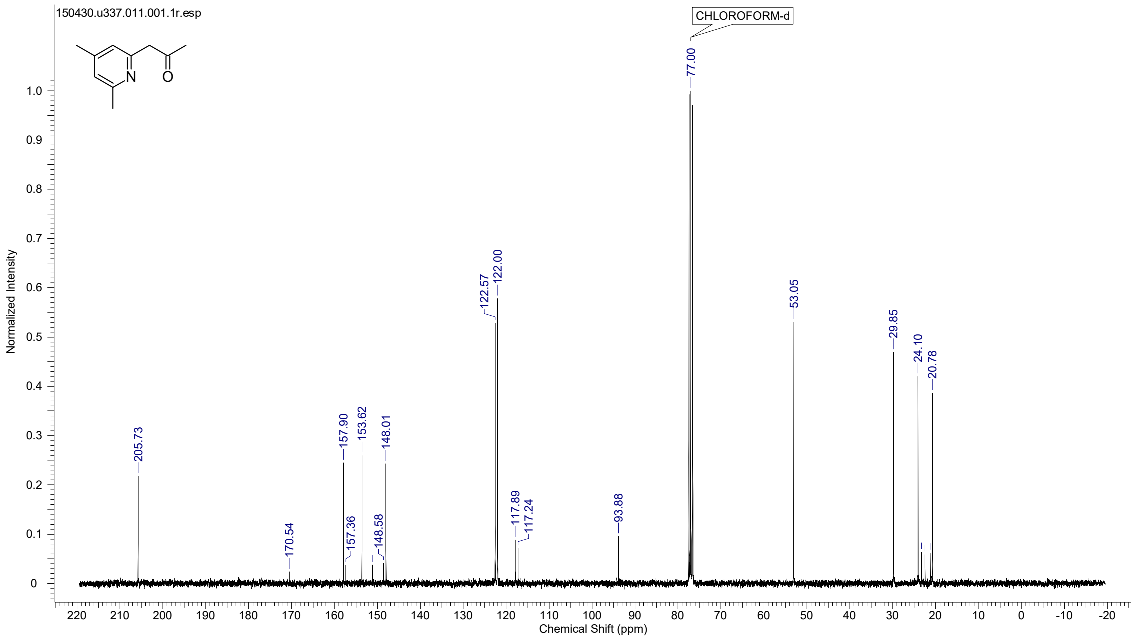

Acquisition Time (sec)	5.2953						
Date Stamp	02 Jun 2015 12:27:12						
Frequency (MHz)	300.13	*Nucleus*	1H	*Number of Transients*	16	*Origin*	spect
Original Points Count	32768	*Owner*	nmrsu	*Points Count*	32768	*Pulse Sequence*	zg30
Receiver Gain	90.50	*SW(cyclical) (Hz)*	6188.12	*Solvent*	CHLOROFORM-d		
Spectrum Offset (Hz)	1850.4254	*Spectrum Type*	STANDARD	*Sweep Width (Hz)*	6187.93	*Temperature (degree C)*	25.160

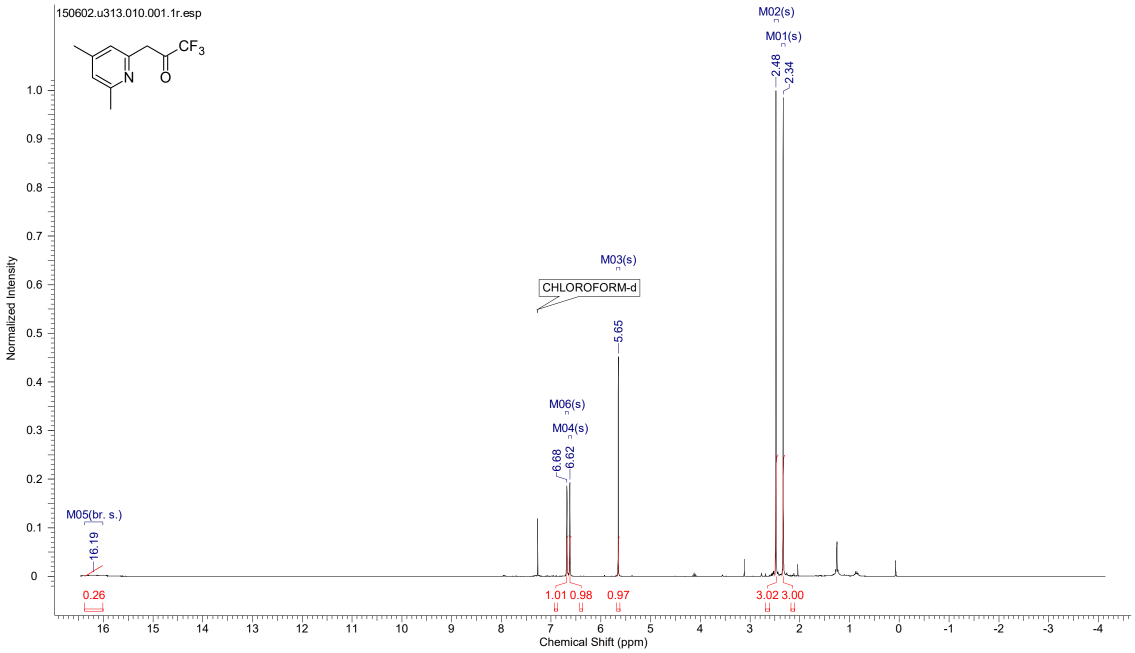

Acquisition Time (sec)	1.8175						
Date Stamp	02 Jun 2015 18:10:40						
Frequency (MHz)	75.47	*Nucleus*	13C	*Number of Transients*	1024	*Origin*	spect
Original Points Count	32768	*Owner*	nmrsu	*Points Count*	32768	*Pulse Sequence*	zgpg30
Receiver Gain	2050.00	*SW(cyclical) (Hz)*	18028.85	*Solvent*	CHLOROFORM-d		
Spectrum Offset (Hz)	7543.8608	*Spectrum Type*	STANDARD	*Sweep Width (Hz)*	18028.29	*Temperature (degree C)*	25.460

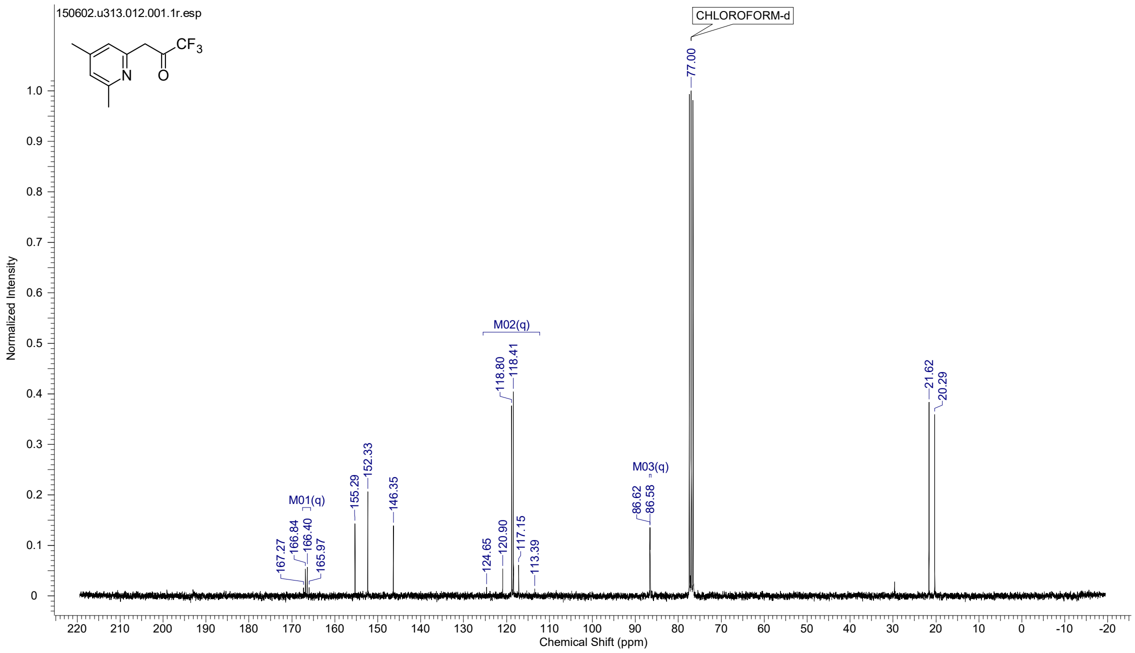

6. NMR-spectra for racemic alcohols (rac-3)

Acquisition Time (sec)	5.2953						
Date Stamp	19 Jun 2015 09:30:08						
Frequency (MHz)	300.13	*Nucleus*	1H	*Number of Transients*	16	*Origin*	spect
Original Points Count	32768	*Owner*	nmrsu	*Points Count*	32768	*Pulse Sequence*	zg30
Receiver Gain	25.40	*SW(cyclical) (Hz)*	6188.12	*Solvent*	CHLOROFORM-d		
Spectrum Offset (Hz)	1850.6140	*Spectrum Type*	STANDARD	*Sweep Width (Hz)*	6187.93	*Temperature (degree C)*	25.160

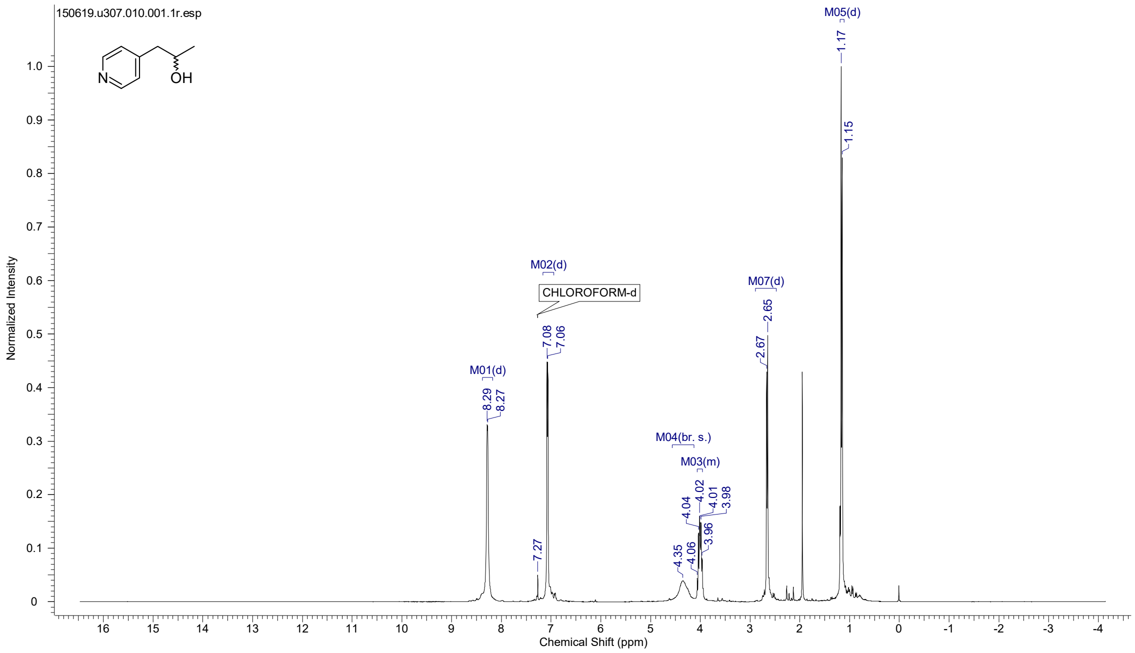

Acquisition Time (sec)	1.8175						
Date Stamp	19 Jun 2015 16:13:20						
Frequency (MHz)	75.47	*Nucleus*	13C	*Number of Transients*	1024	*Origin*	spect
Original Points Count	32768	*Owner*	nmrsu	*Points Count*	32768	*Pulse Sequence*	zgpg30
Receiver Gain	2050.00	*SW(cyclical) (Hz)*	18028.85	*Solvent*	CHLOROFORM-d		
Spectrum Offset (Hz)	7533.9575	*Spectrum Type*	STANDARD	*Sweep Width (Hz)*	18028.29	*Temperature (degree C)*	25.360

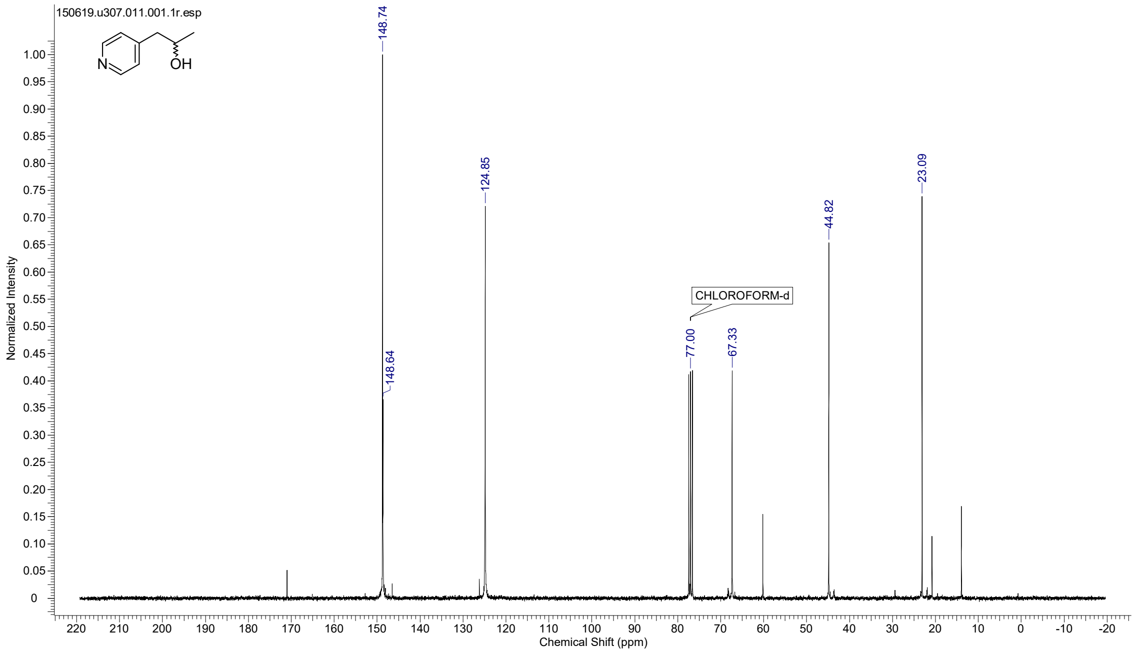

Acquisition Time (sec)	5.2953						
Date Stamp	29 Jul 2015 08:13:20						
Frequency (MHz)	300.13	Nucleus	1H	Number of Transients	16	Origin	spect
Original Points Count	32768	Owner	nmrsu	Points Count	32768	Pulse Sequence	zg30
Receiver Gain	128.00	SW(cyclical) (Hz)	6188.12	Solvent	CHLOROFORM-d		
Spectrum Offset (Hz)	1850.4255	Spectrum Type	STANDARD	Sweep Width (Hz)	6187.93	Temperature (degree C)	25.160

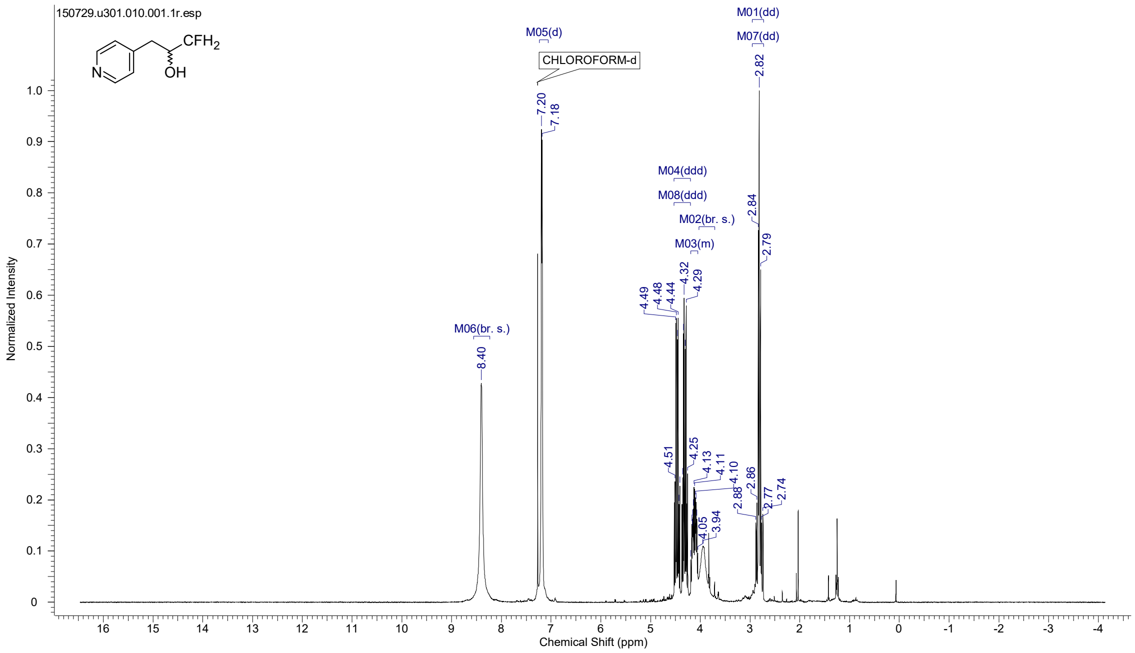

Acquisition Time (sec)	1.8175						
Date Stamp	29 Jul 2015 11:48:48						
Frequency (MHz)	75.47	Nucleus	13C	Number of Transients	1024	Origin	spect
Original Points Count	32768	Owner	nmrsu	Points Count	32768	Pulse Sequence	zgpg30
Receiver Gain	2050.00	SW(cyclical) (Hz)	18028.85	Solvent	CHLOROFORM-d		
Spectrum Offset (Hz)	7543.3115	Spectrum Type	STANDARD	Sweep Width (Hz)	18028.29	Temperature (degree C)	25.660

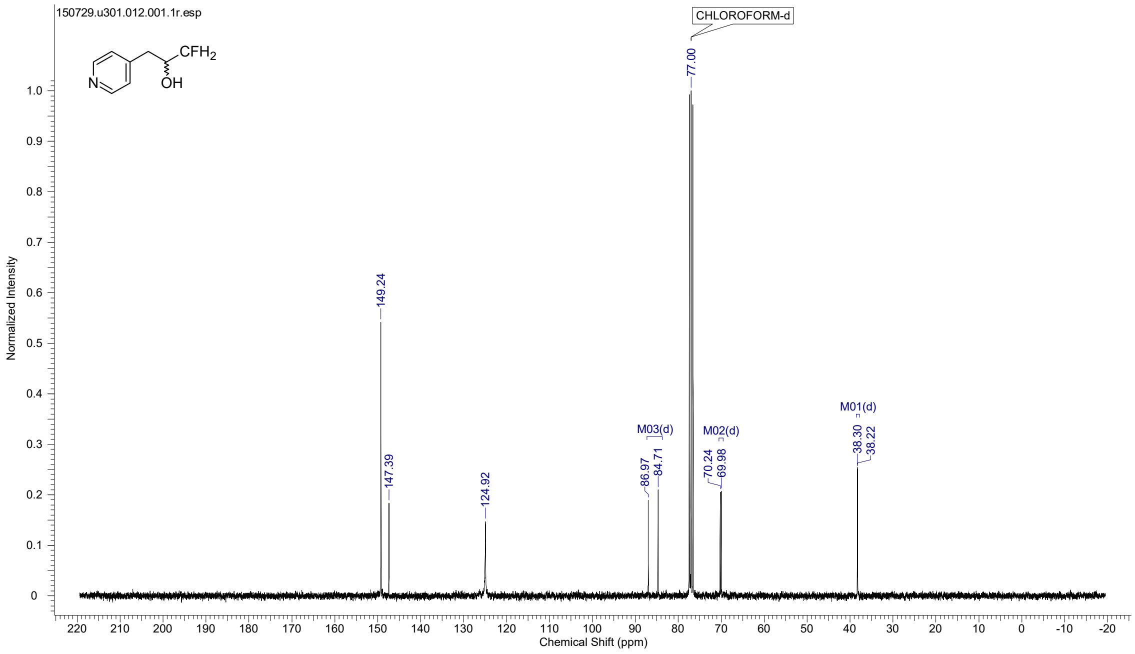

Acquisition Time (sec)	6.3439						
Date Stamp	30 Jul 2015 09:38:40						
Frequency (MHz)	250.13	*Nucleus*	1H	*Number of Transients*	16	*Origin*	spect
Original Points Count	32768	*Owner*	nmr	*Points Count*	32768	*Pulse Sequence*	zg30
Receiver Gain	203.00	*SW(cyclical) (Hz)*	5165.29	*Solvent*	CHLOROFORM-d		
Spectrum Offset (Hz)	1532.7719	*Spectrum Type*	STANDARD	*Sweep Width (Hz)*	5165.13	*Temperature (degree C)*	24.960

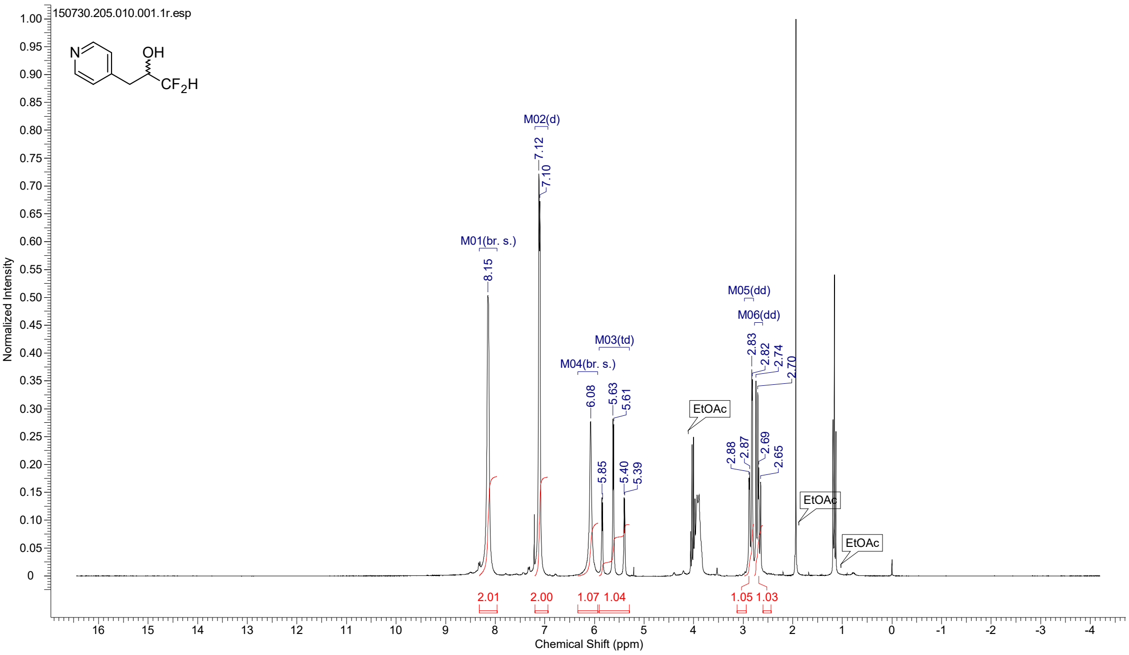

Acquisition Time (sec)	2.1845						
Date Stamp	29 Jul 2015 14:16:00						
Frequency (MHz)	62.90	*Nucleus*	13C	*Number of Transients*	1024	*Origin*	spect
Original Points Count	32768	*Owner*	nmr	*Points Count*	32768	*Pulse Sequence*	zgpg30
Receiver Gain	2050.00	*SW(cyclical) (Hz)*	15000.00	*Solvent*	CHLOROFORM-d		
Spectrum Offset (Hz)	6031.5171	*Spectrum Type*	STANDARD	*Sweep Width (Hz)*	14999.54	*Temperature (degree C)*	25.060

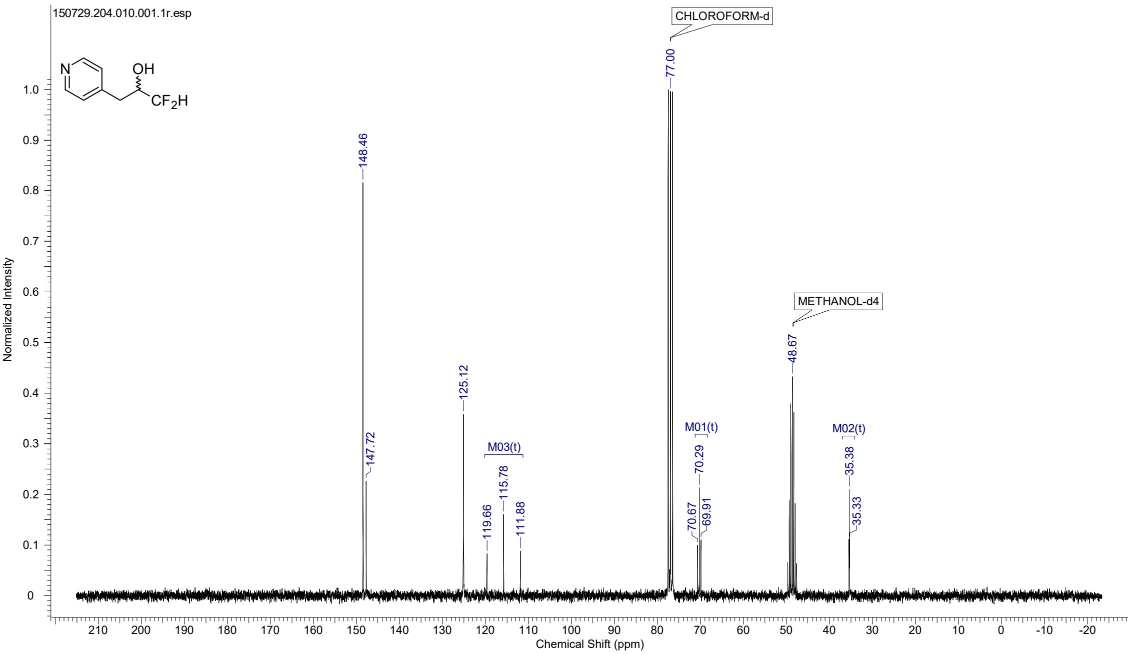

Acquisition Time (sec)	5.2953						
Date Stamp	18 Jun 2015 08:38:56						
Frequency (MHz)	300.13	Nucleus	1H	Number of Transients	16	Origin	spect
Original Points Count	32768	Owner	nmrsu	Points Count	32768	Pulse Sequence	zg30
Receiver Gain	228.00	SW(cyclical) (Hz)	6188.12	Solvent	CHLOROFORM-d		
Spectrum Offset (Hz)	1850.4254	Spectrum Type	STANDARD	Sweep Width (Hz)	6187.93	Temperature (degree C)	25.160

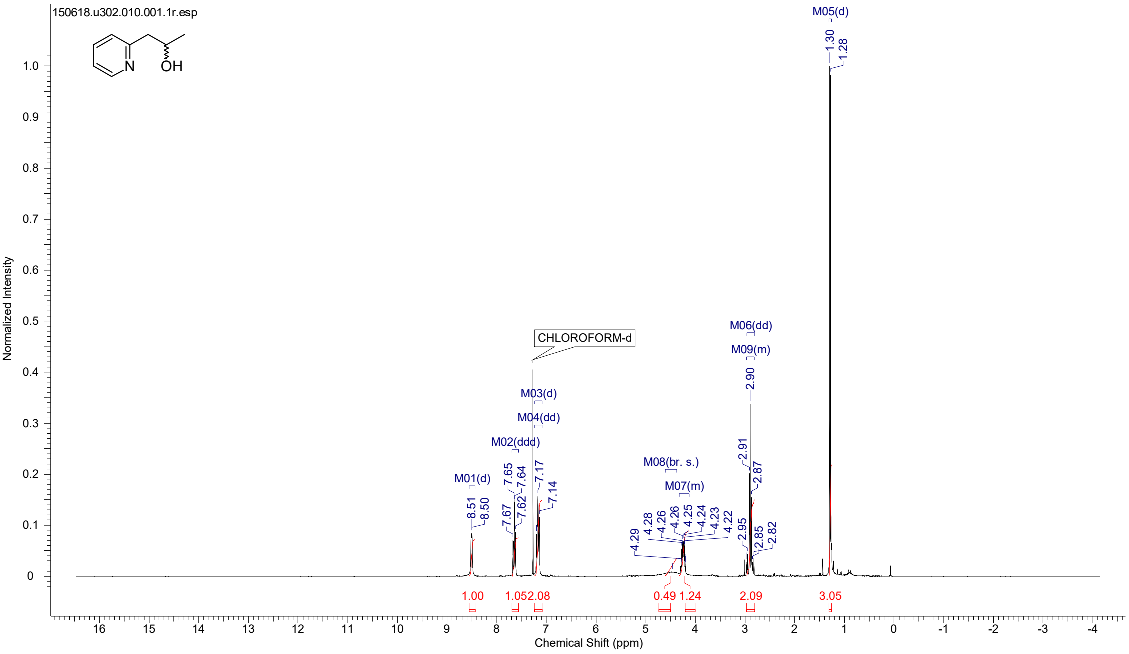

Acquisition Time (sec)	2.1845						
Date Stamp	18 Jun 2015 16:21:52						
Frequency (MHz)	62.90	Nucleus	13C	Number of Transients	1024	Origin	spect
Original Points Count	32768	Owner	nmr	Points Count	32768	Pulse Sequence	zgpg30
Receiver Gain	2050.00	SW(cyclical) (Hz)	15000.00	Solvent	CHLOROFORM-d		
Spectrum Offset (Hz)	6289.2378	Spectrum Type	STANDARD	Sweep Width (Hz)	14999.54	Temperature (degree C)	25.060

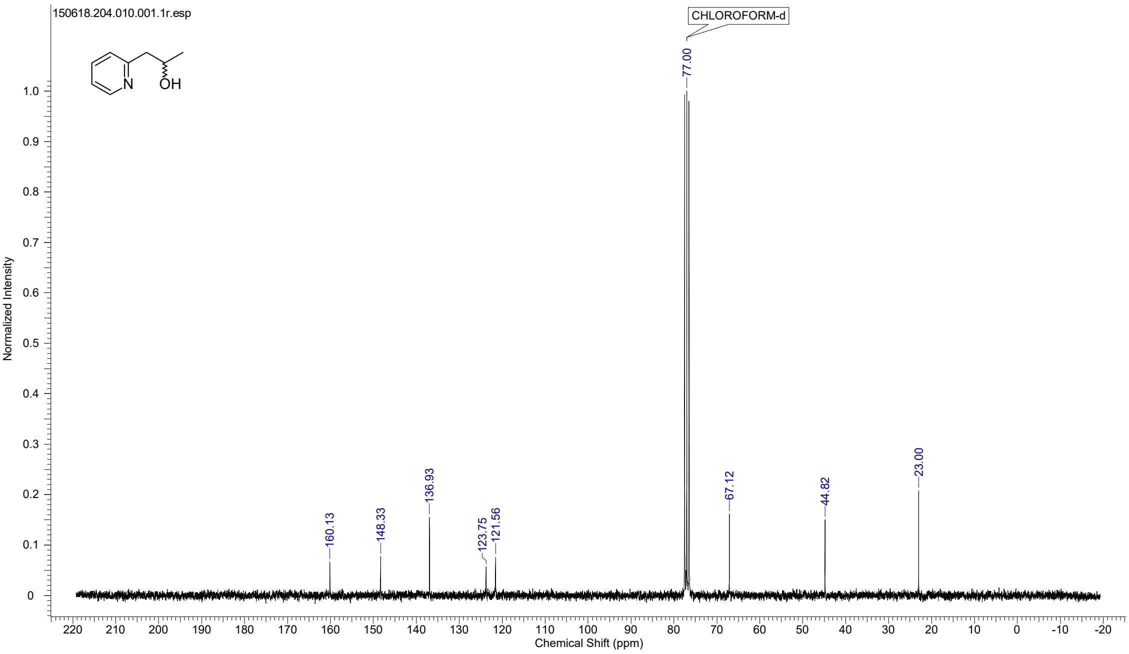

Acquisition Time (sec)	5.2953						
Date Stamp	28 Jul 2015 16:00:32						
Frequency (MHz)	300.13	***Nucleus***	1H	***Number of Transients***	16	***Origin***	spect
Original Points Count	32768	***Owner***	nmrsu	***Points Count***	32768	***Pulse Sequence***	zg30
Receiver Gain	90.50	***SW(cyclical) (Hz)***	6188.12	***Solvent***	CHLOROFORM-d		
Spectrum Offset (Hz)	1850.4254	***Spectrum Type***	STANDARD	***Sweep Width (Hz)***	6187.93	***Temperature (degree C)***	25.360

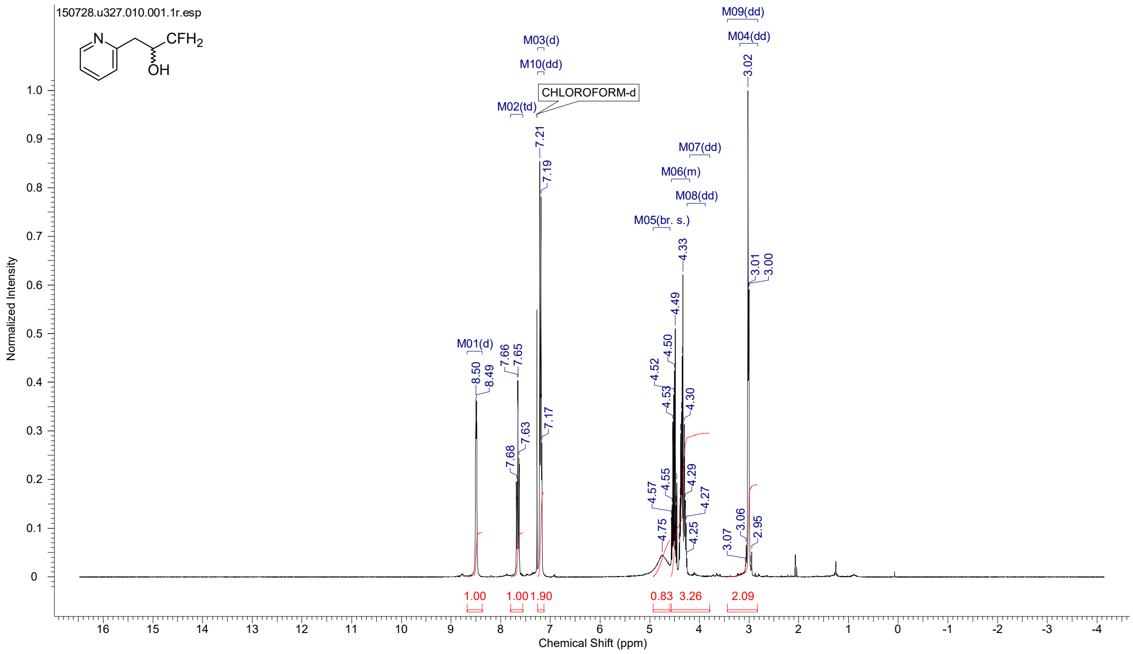

Acquisition Time (sec)	2.1845						
Date Stamp	29 Jul 2015 12:40:00						
Frequency (MHz)	62.90	***Nucleus***	13C	***Number of Transients***	1024	***Origin***	spect
Original Points Count	32768	***Owner***	nmr	***Points Count***	32768	***Pulse Sequence***	zgpg30
Receiver Gain	2050.00	***SW(cyclical) (Hz)***	15000.00	***Solvent***	CHLOROFORM-d		
Spectrum Offset (Hz)	6287.8647	***Spectrum Type***	STANDARD	***Sweep Width (Hz)***	14999.54	***Temperature (degree C)***	25.060

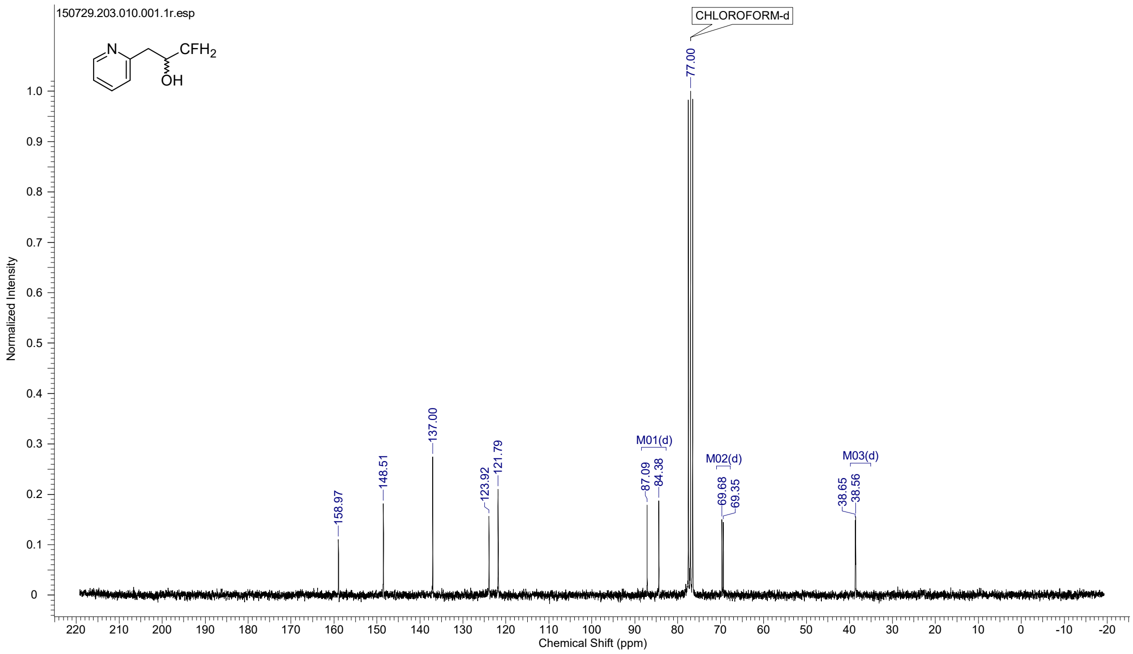

Acquisition Time (sec)	6.3439						
Date Stamp	22 Jun 2015 16:02:40						
Frequency (MHz)	250.13	*Nucleus*	1H	*Number of Transients*	16	*Origin*	spect
Original Points Count	32768	*Owner*	nmr	*Points Count*	32768	*Pulse Sequence*	zg30
Receiver Gain	456.00	*SW(cyclical) (Hz)*	5165.29	*Solvent*	CHLOROFORM-d		
Spectrum Offset (Hz)	1546.6086	*Spectrum Type*	STANDARD	*Sweep Width (Hz)*	5165.13	*Temperature (degree C)*	25.160

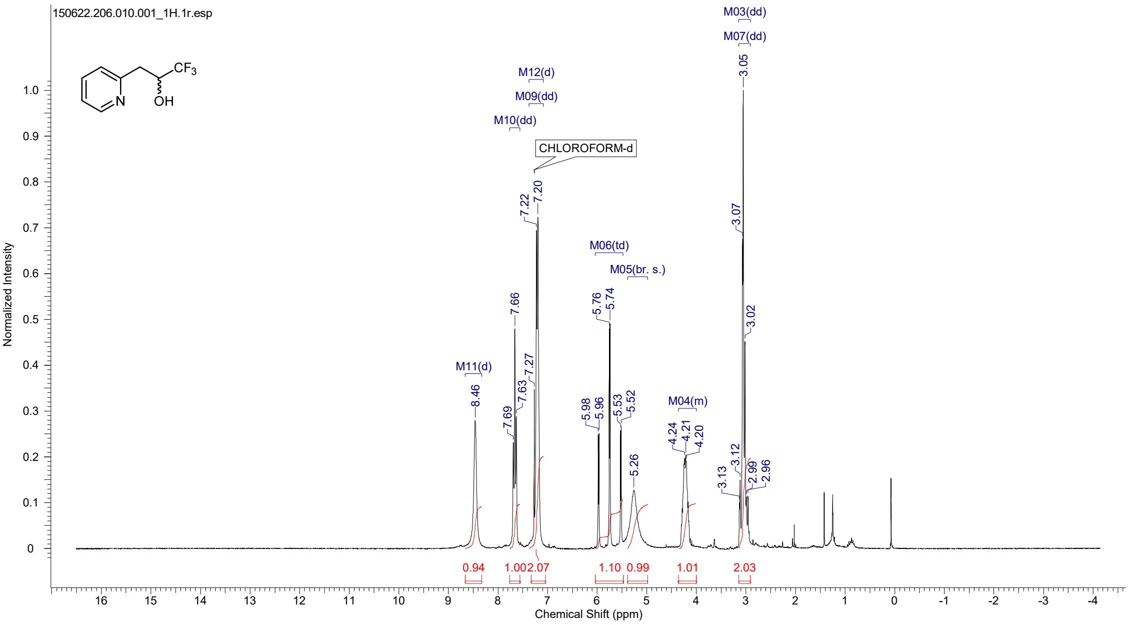

Acquisition Time (sec)	2.1845						
Date Stamp	23 Jun 2015 22:54:24						
Frequency (MHz)	62.90	*Nucleus*	13C	*Number of Transients*	1024	*Origin*	spect
Original Points Count	32768	*Owner*	nmr	*Points Count*	32768	*Pulse Sequence*	zgpg30
Receiver Gain	2050.00	*SW(cyclical) (Hz)*	15000.00	*Solvent*	CHLOROFORM-d		
Spectrum Offset (Hz)	6286.0337	*Spectrum Type*	STANDARD	*Sweep Width (Hz)*	14999.54	*Temperature (degree C)*	25.060

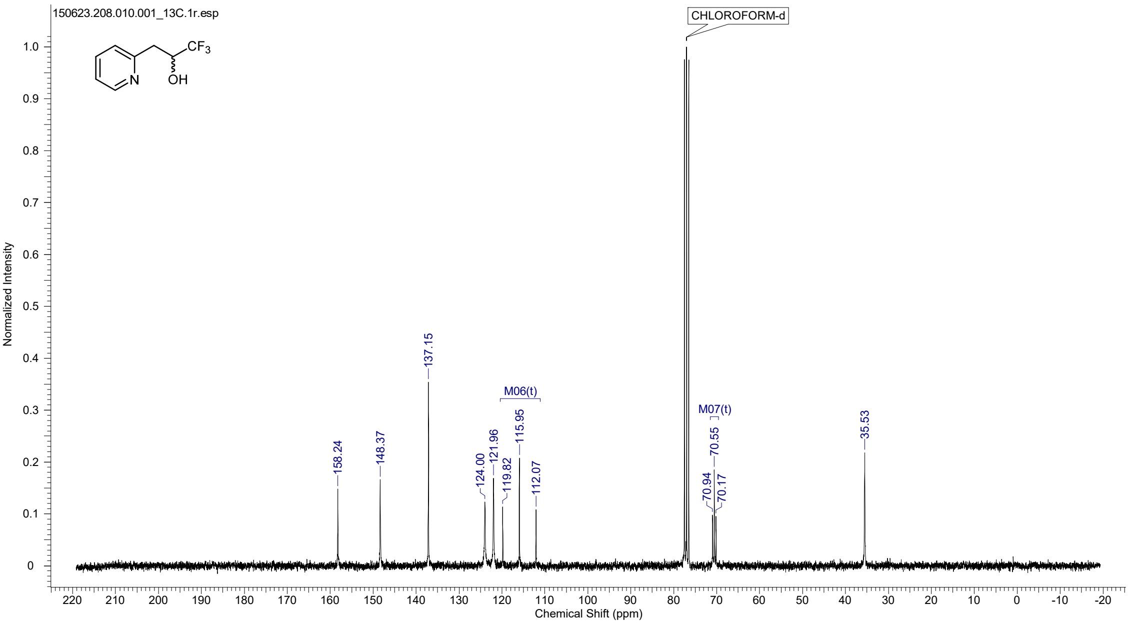

7. NMR Spectra for enantioenriched alcohols (3)

Acquisition Time (sec)	6.3439						
Date Stamp	08 May 2015 15:49:52						
Frequency (MHz)	250.13	*Nucleus*	1H	*Number of Transients*	16	*Origin*	spect
Original Points Count	32768	*Owner*	nmr	*Points Count*	32768	*Pulse Sequence*	zg30
Receiver Gain	362.00	*SW(cyclical) (Hz)*	5165.29	*Solvent*	CHLOROFORM-d		
Spectrum Offset (Hz)	1546.6085	*Spectrum Type*	STANDARD	*Sweep Width (Hz)*	5165.13	*Temperature (degree C)*	25.160

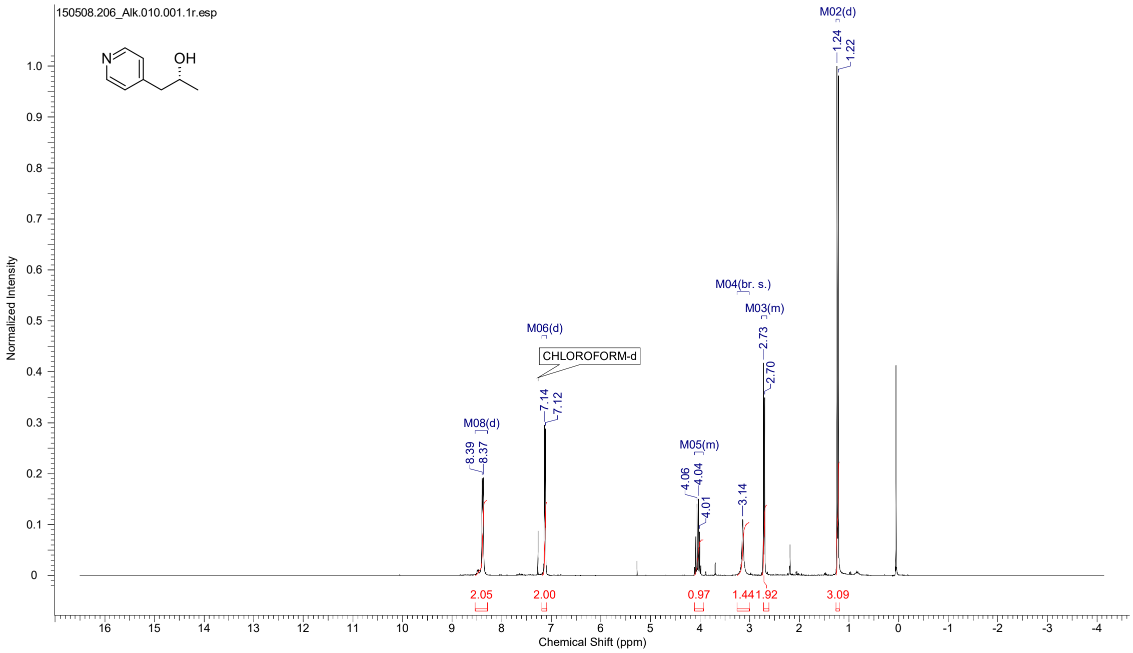

Acquisition Time (sec)	2.1845						
Date Stamp	08 May 2015 17:04:32						
Frequency (MHz)	62.90	*Nucleus*	13C	*Number of Transients*	1024	*Origin*	spect
Original Points Count	32768	*Owner*	nmr	*Points Count*	32768	*Pulse Sequence*	zgpg30
Receiver Gain	2050.00	*SW(cyclical) (Hz)*	15000.00	*Solvent*	CHLOROFORM-d		
Spectrum Offset (Hz)	6285.5806	*Spectrum Type*	STANDARD	*Sweep Width (Hz)*	14999.54	*Temperature (degree C)*	25.060

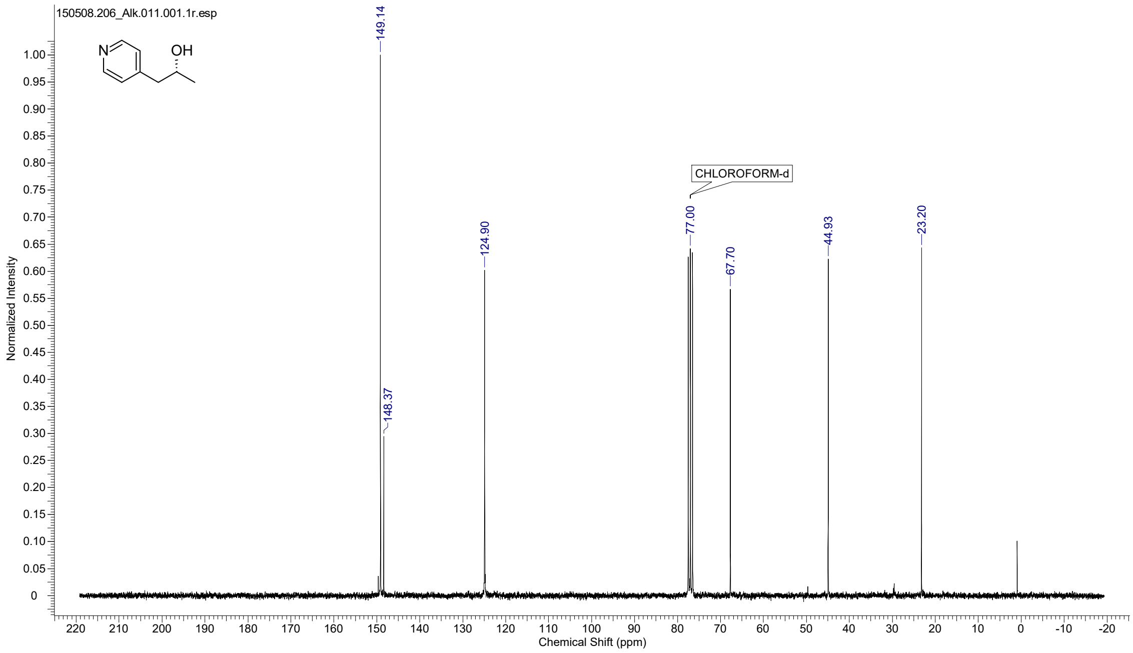

Acquisition Time (sec)	5.2953						
Date Stamp	03 Aug 2015 11:23:12						
Frequency (MHz)	300.13	*Nucleus*	1H	*Number of Transients*	16	*Origin*	spect
Original Points Count	32768	*Owner*	nmrsu	*Points Count*	32768	*Pulse Sequence*	zg30
Receiver Gain	50.80	*SW(cyclical) (Hz)*	6188.12	*Solvent*	METHANOL-d4	*Spectrum Offset (Hz)*	1808.5013
Spectrum Type	STANDARD	*Sweep Width (Hz)*	6187.93	*Temperature (degree C)*	25.160		

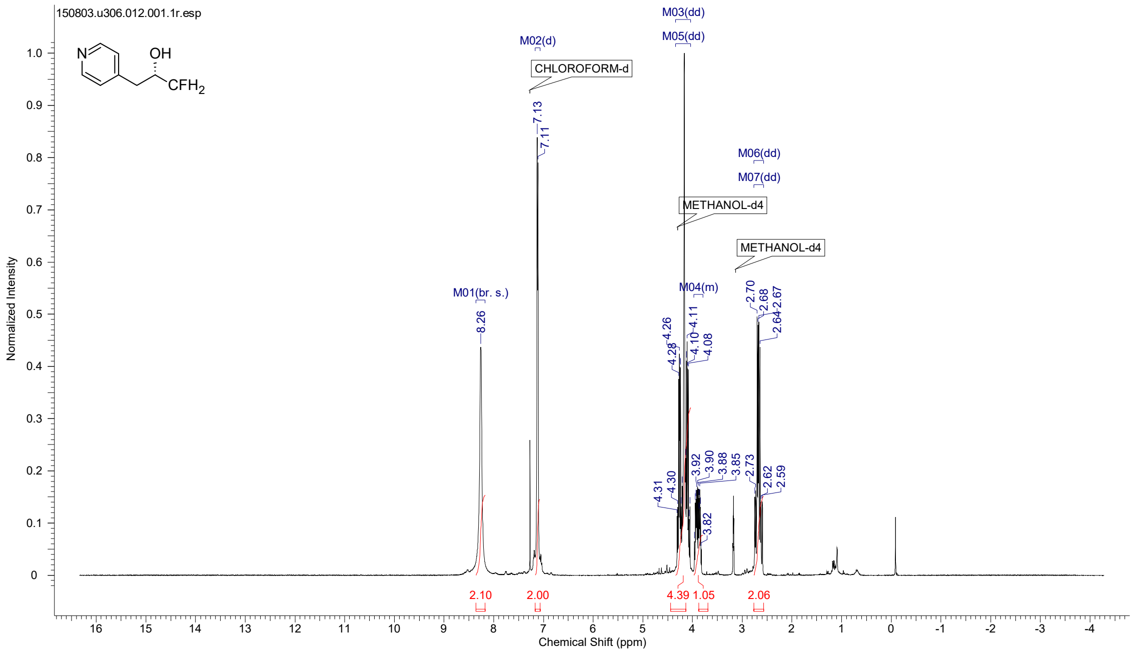

Nucleus	13C	*Number of Transients*	1024	*Origin*	spect
Owner	nmrsu	*Points Count*	32768	*Pulse Sequence*	zgpg30
SW(cyclical) (Hz)	18028.85	*Solvent*	METHANOL-d4	*Spectrum Offset (Hz)*	7525.1460
Sweep Width (Hz)	18028.29	*Temperature (degree C)*	25.860		

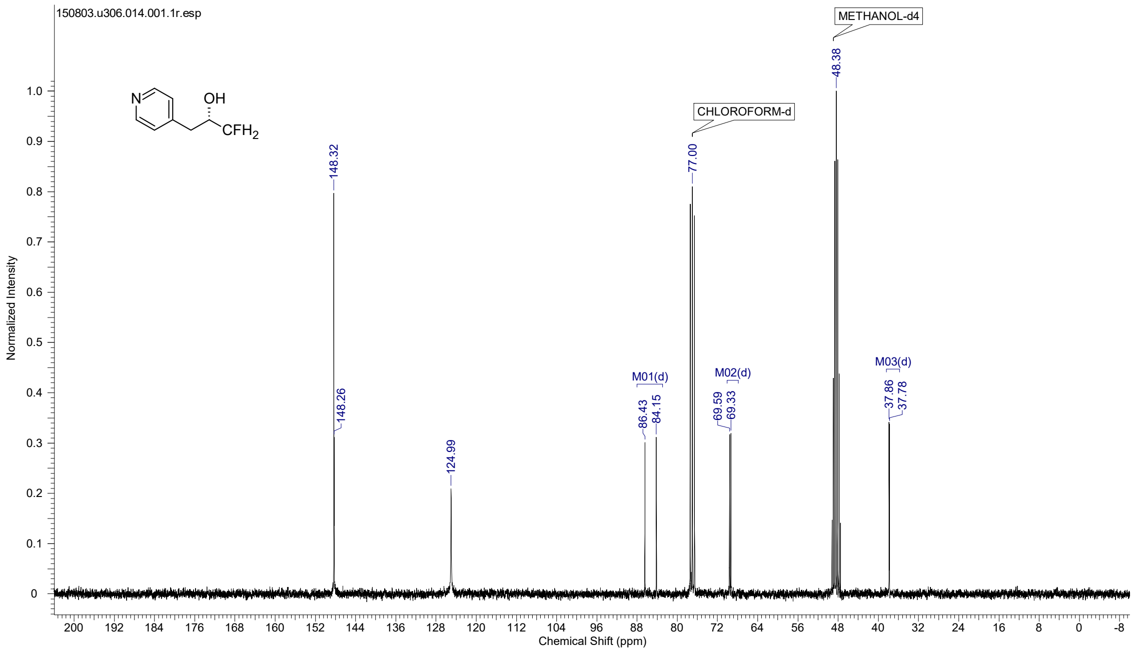

Acquisition Time (sec)	5.2953						
Date Stamp	31 Jul 2015 14:30:56						
Frequency (MHz)	300.13	***Nucleus***	1H	***Number of Transients***	16	***Origin***	spect
Original Points Count	32768	***Owner***	nmrsu	***Points Count***	32768	***Pulse Sequence***	zg30
Receiver Gain	50.80	***SW(cyclical) (Hz)***	6188.12	***Solvent***	Acetone	***Spectrum Offset (Hz)***	1849.2833
Spectrum Type	STANDARD	***Sweep Width (Hz)***	6187.93	***Temperature (degree C)***	25.160		

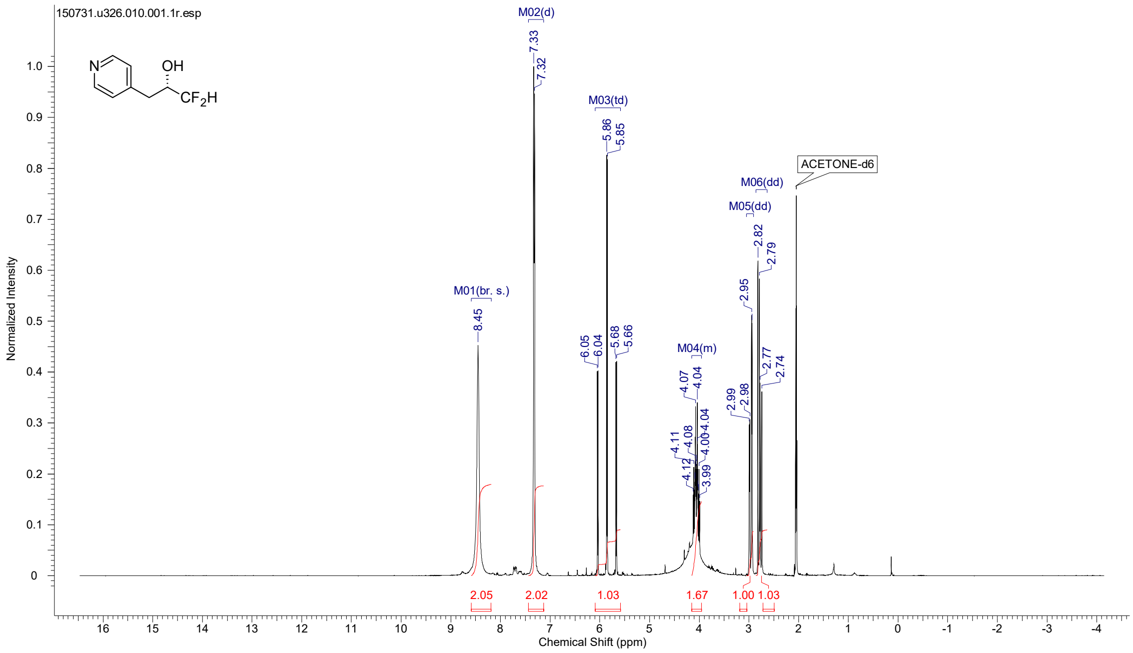

Acquisition Time (sec)	2.1845						
Date Stamp	03 Aug 2015 13:12:00						
Frequency (MHz)	62.90	***Nucleus***	13C	***Number of Transients***	1024	***Origin***	spect
Original Points Count	32768	***Owner***	nmr	***Points Count***	32768	***Pulse Sequence***	zgpg30
Receiver Gain	2050.00	***SW(cyclical) (Hz)***	15000.00	***Solvent***	Acetone	***Spectrum Offset (Hz)***	6350.2837
Spectrum Type	STANDARD	***Sweep Width (Hz)***	14999.54	***Temperature (degree C)***	25.060		

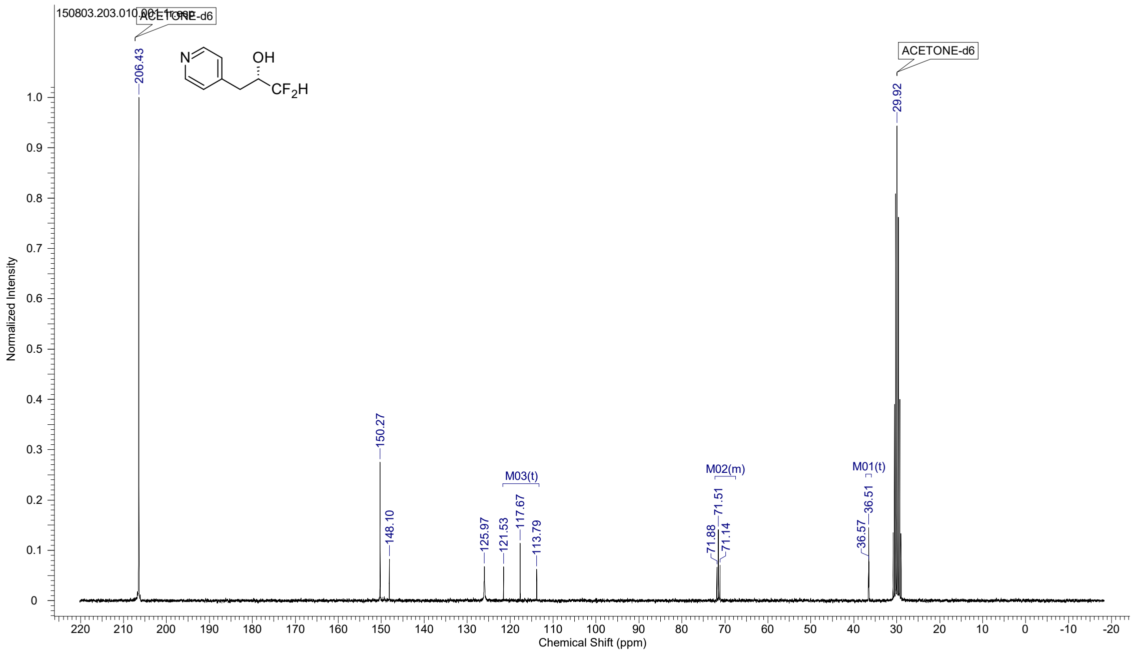

Acquisition Time (sec)	5.2953						
Date Stamp	06 Aug 2015 08:43:12						
Frequency (MHz)	300.13	*Nucleus*	1H	*Number of Transients*	16	*Origin*	spect
Original Points Count	32768	*Owner*	nmrsu	*Points Count*	32768	*Pulse Sequence*	zg30
Receiver Gain	228.00	*SW(cyclical) (Hz)*	6188.12	*Solvent*	CHLOROFORM-d		
Spectrum Offset (Hz)	1850.4257	*Spectrum Type*	STANDARD	*Sweep Width (Hz)*	6187.93	*Temperature (degree C)*	25.160

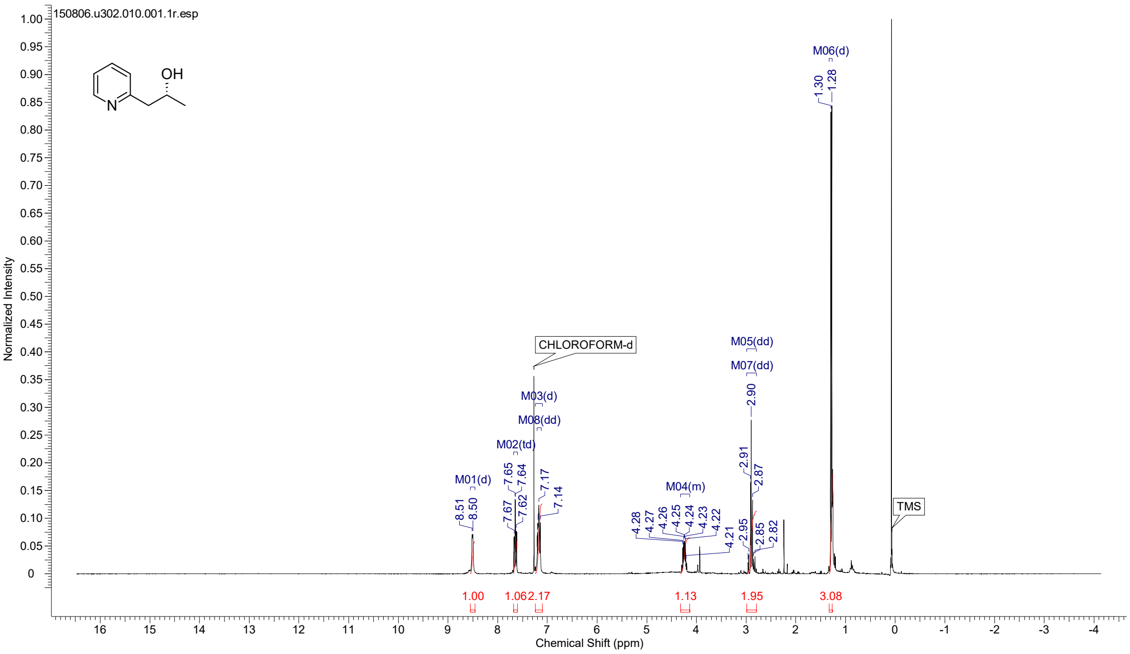

Acquisition Time (sec)	2.1845						
Date Stamp	06 Aug 2015 20:29:20						
Frequency (MHz)	62.90	*Nucleus*	13C	*Number of Transients*	1024	*Origin*	spect
Original Points Count	32768	*Owner*	nmr	*Points Count*	32768	*Pulse Sequence*	zgpg30
Receiver Gain	2050.00	*SW(cyclical) (Hz)*	15000.00	*Solvent*	CHLOROFORM-d		
Spectrum Offset (Hz)	6289.2422	*Spectrum Type*	STANDARD	*Sweep Width (Hz)*	14999.54	*Temperature (degree C)*	25.360

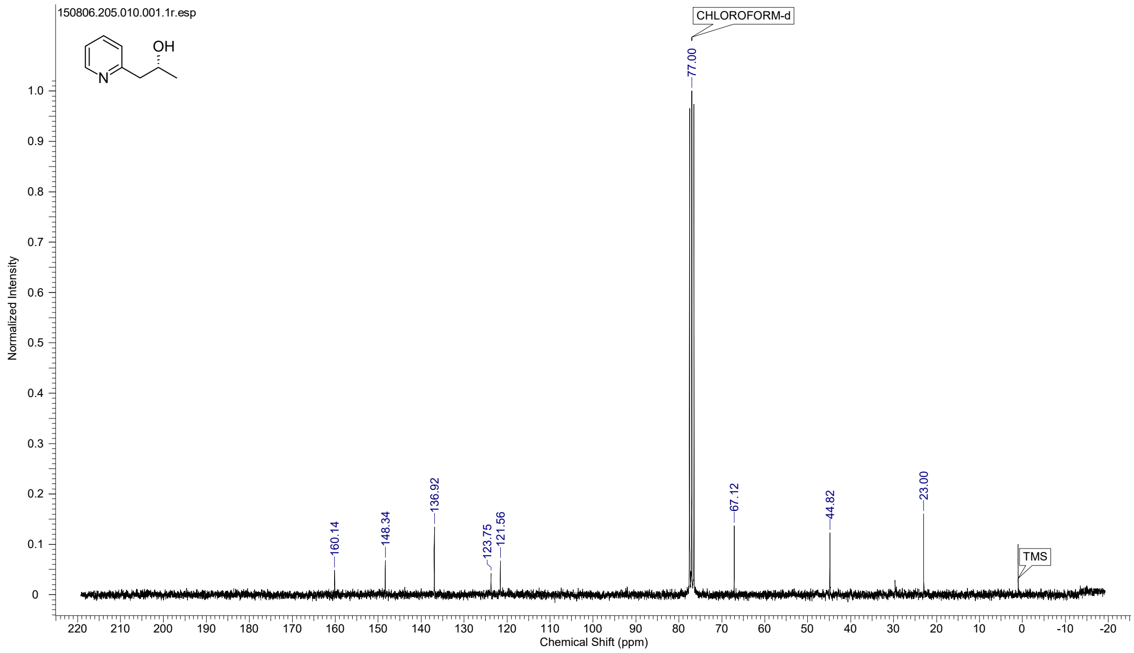

Acquisition Time (sec)	5.2953						
Date Stamp	05 Aug 2015 15:49:52						
Frequency (MHz)	300.13	Nucleus	1H	Number of Transients	16	Origin	spect
Original Points Count	32768	Owner	nmrsu	Points Count	32768	Pulse Sequence	zg30
Receiver Gain	287.00	SW(cyclical) (Hz)	6188.12	Solvent	CHLOROFORM-d		
Spectrum Offset (Hz)	1850.4255	Spectrum Type	STANDARD	Sweep Width (Hz)	6187.93	Temperature (degree C)	25.960

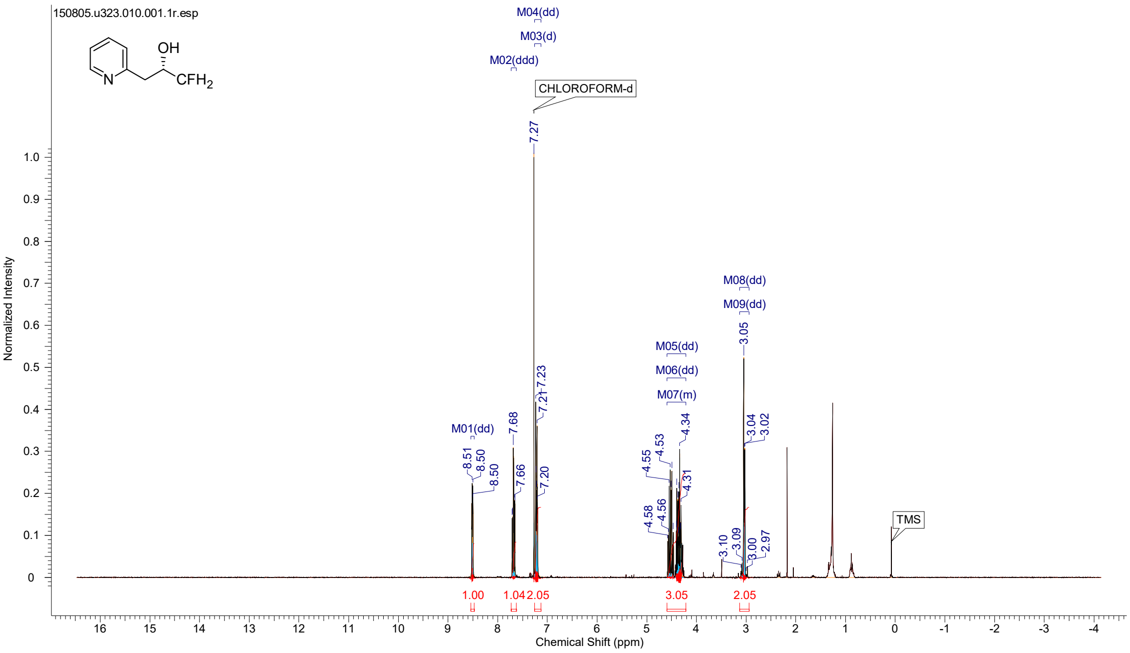

Acquisition Time (sec)	2.1845						
Date Stamp	07 Aug 2015 06:54:24						
Frequency (MHz)	62.90	Nucleus	13C	Number of Transients	1024	Origin	spect
Original Points Count	32768	Owner	nmr	Points Count	32768	Pulse Sequence	zgpg30
Receiver Gain	2050.00	SW(cyclical) (Hz)	15000.00	Solvent	CHLOROFORM-d		
Spectrum Offset (Hz)	6289.2378	Spectrum Type	STANDARD	Sweep Width (Hz)	14999.54	Temperature (degree C)	25.060

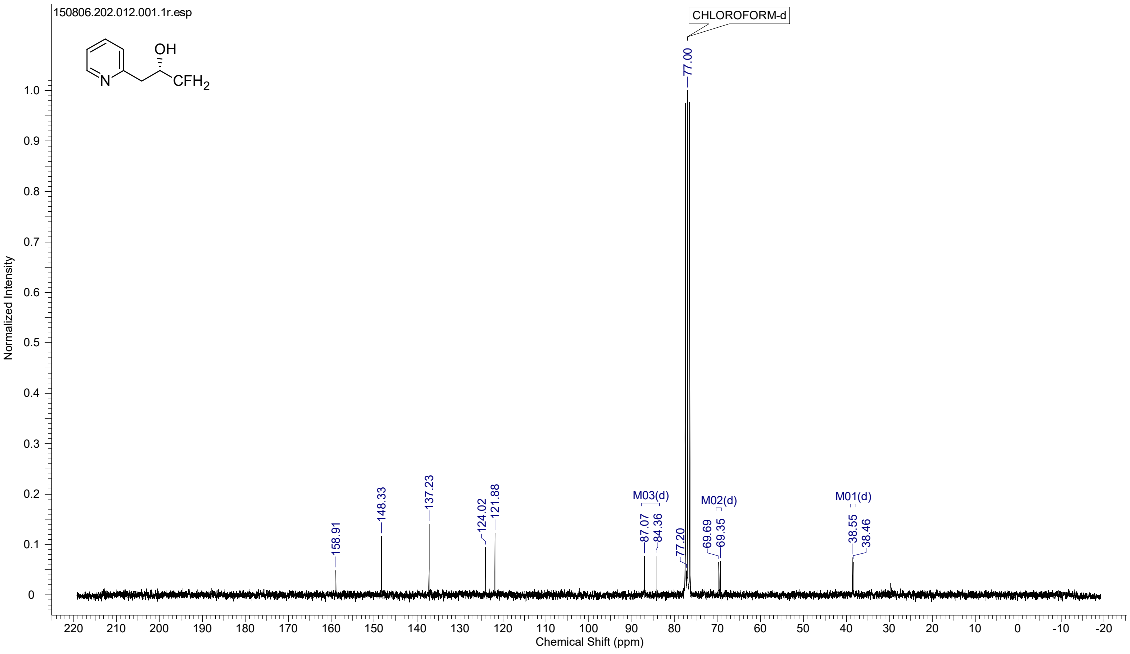

Acquisition Time (sec)	5.2953						
Date Stamp	09 Jul 2015 08:51:44						
Frequency (MHz)	300.13	*Nucleus*	1H	*Number of Transients*	16	*Origin*	spect
Original Points Count	32768	*Owner*	nmrsu	*Points Count*	32768	*Pulse Sequence*	zg30
Receiver Gain	144.00	*SW(cyclical) (Hz)*	6188.12	*Solvent*	CHLOROFORM-d		
Spectrum Offset (Hz)	1853.4469	*Spectrum Type*	STANDARD	*Sweep Width (Hz)*	6187.93	*Temperature (degree C)*	25.560

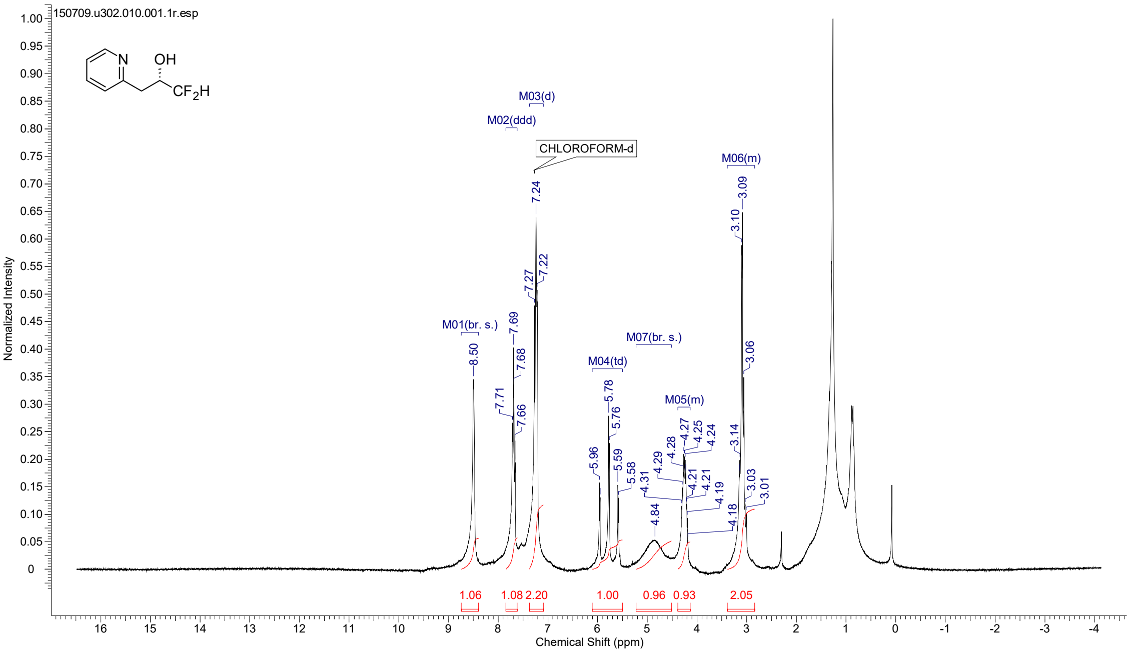

Acquisition Time (sec)	2.1845						
Date Stamp	10 Jul 2015 10:12:48						
Frequency (MHz)	62.90	*Nucleus*	13C	*Number of Transients*	1024	*Origin*	spect
Original Points Count	32768	*Owner*	nmr	*Points Count*	32768	*Pulse Sequence*	zgpg30
Receiver Gain	2050.00	*SW(cyclical) (Hz)*	15000.00	*Solvent*	CHLOROFORM-d		
Spectrum Offset (Hz)	5994.8960	*Spectrum Type*	STANDARD	*Sweep Width (Hz)*	14999.54	*Temperature (degree C)*	25.060

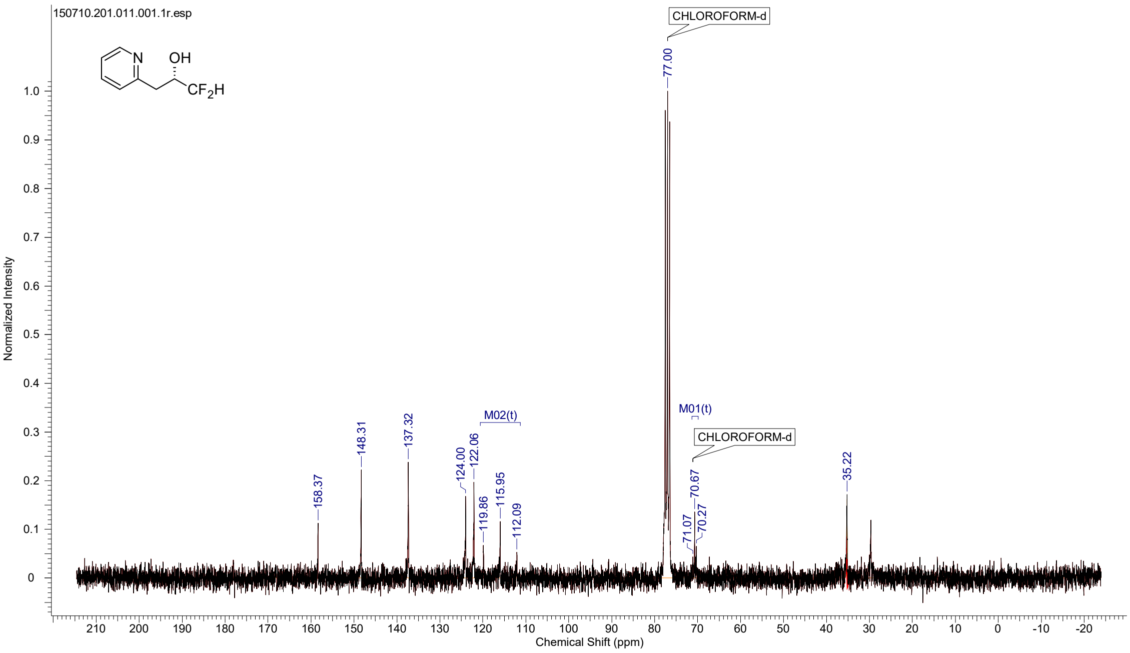

8. Gas chromatograms of chiral alcohols (3)

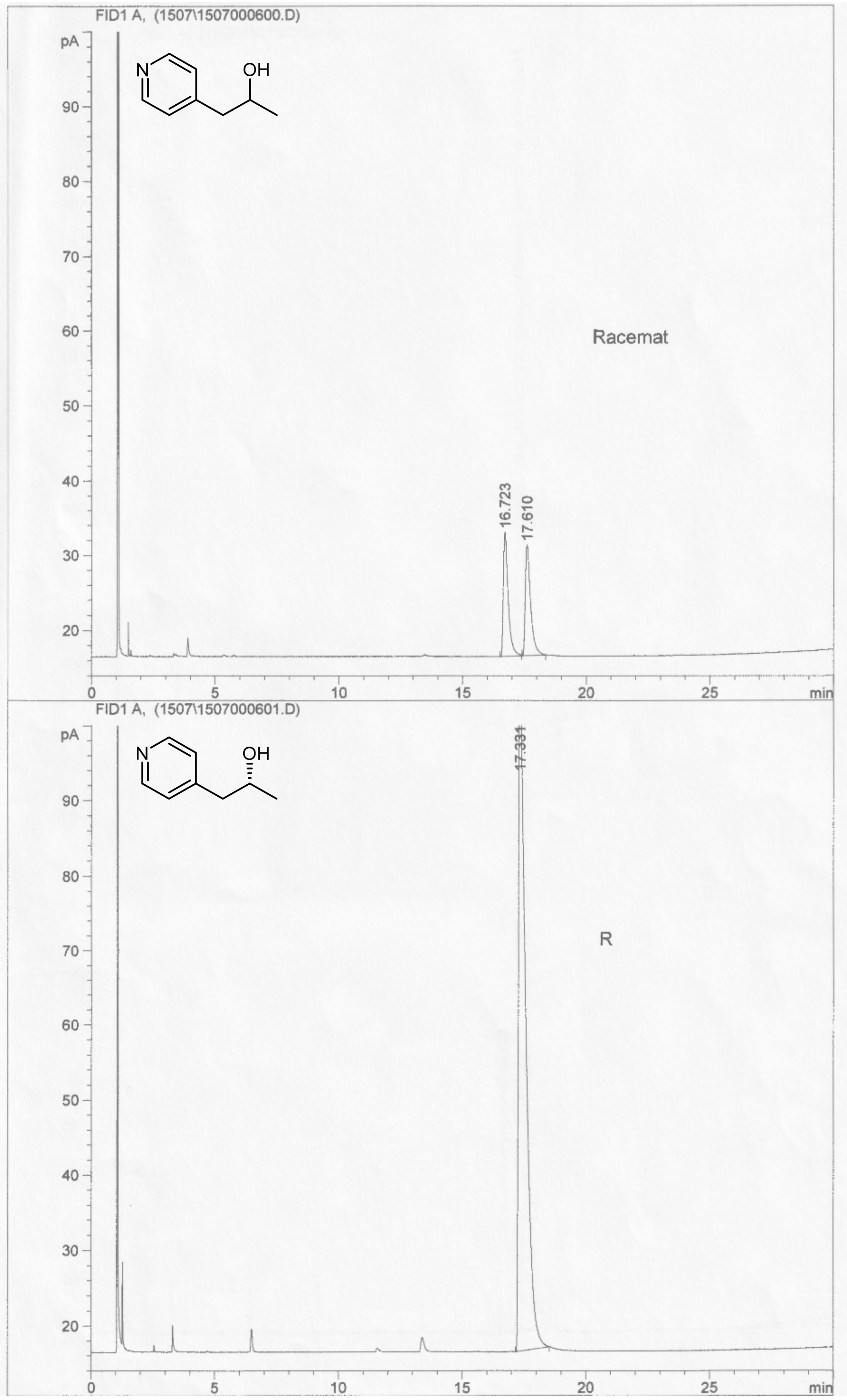

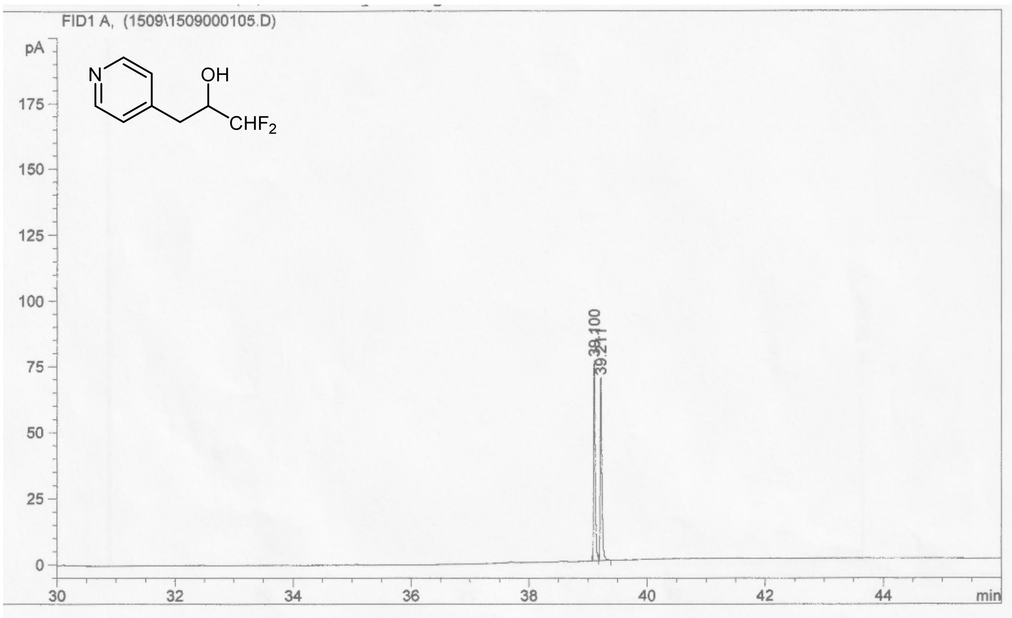
FID1 A, (1509\1509000105.D)
pA
OH
CHF2
39.100
39.211
min

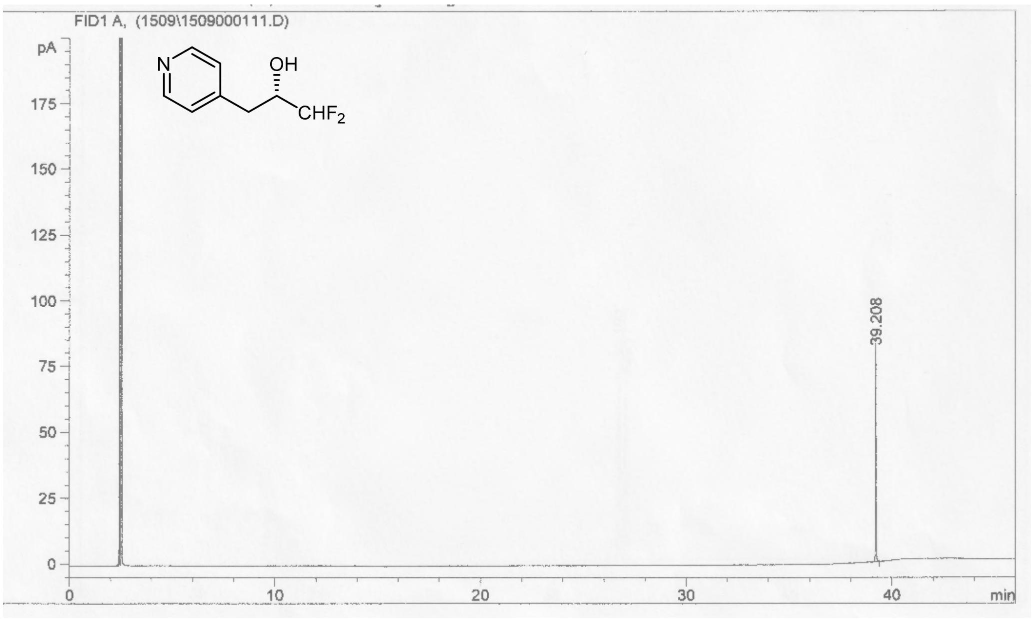
FID1 A, (1509\1509000111.D)
pA
OH
CHF2
39.208
min

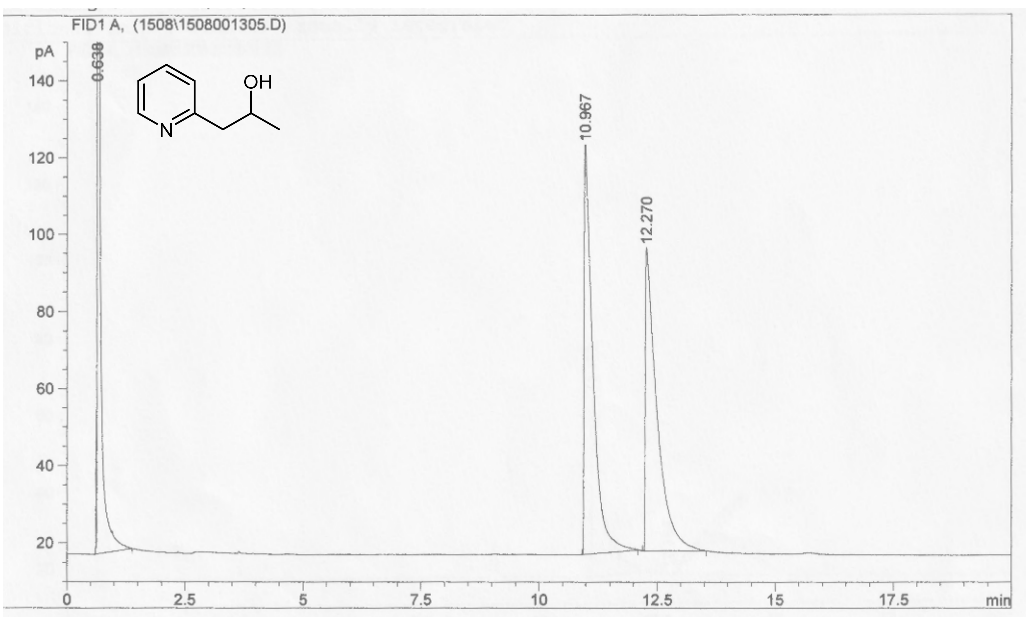
FID1 A, (1508\1508001305.D)
pA
OH
N
0.638
10.967
12.270
min

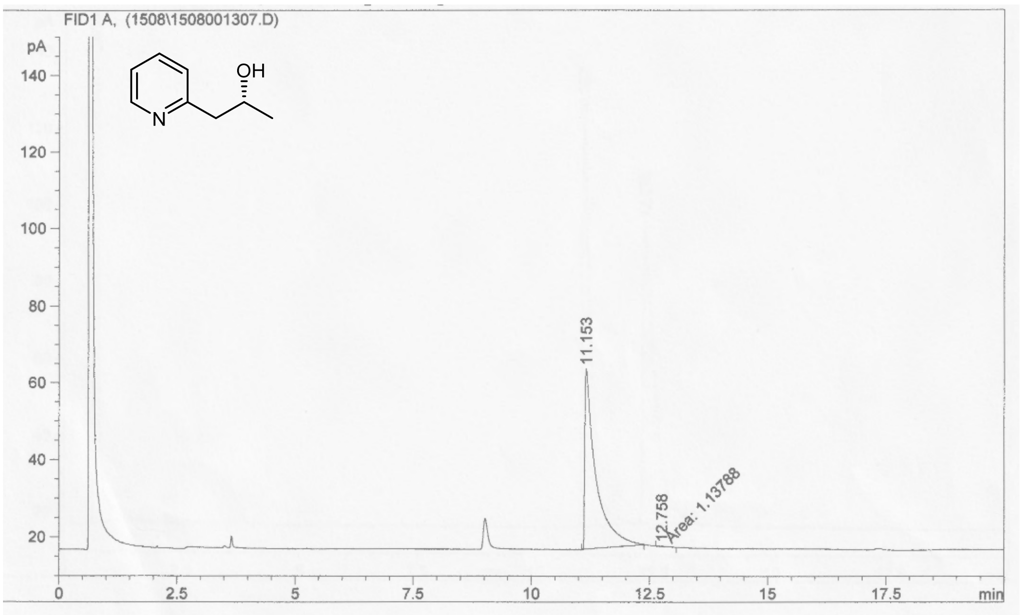
FID1 A, (1508\1508001307.D)
pA
OH
N
11.153
12.758
Area: 1.13788
min

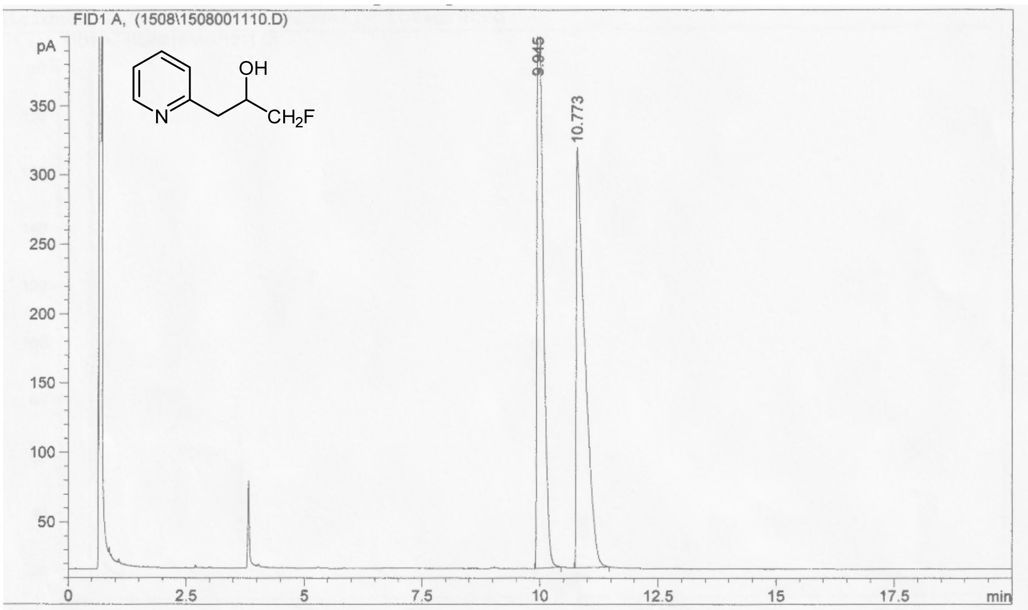
FID1 A, (1508\1508001110.D)
pA
OH
N
CH2F
9.945
10.773
350
300
250
200
150
100
50
0
2.5
5
7.5
10
12.5
15
17.5
min

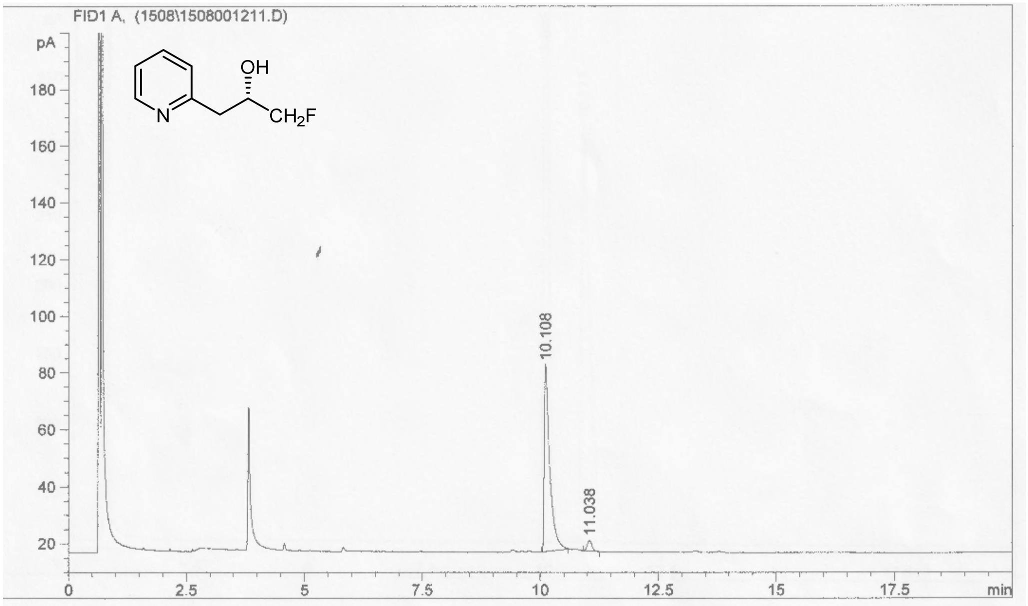
FID1 A, (1508\1508001211.D)
pA
OH
N
CH2F
10.108
11.038
180
160
140
120
100
80
60
40
20
0
2.5
5
7.5
10
12.5
15
17.5
min
